# Triggering of endoplasmic reticulum stress via ATF4-SPHK1 signaling promotes glioblastoma invasion and chemoresistance

**DOI:** 10.1038/s41419-024-06936-8

**Published:** 2024-08-01

**Authors:** Beiwu Lan, Zhoudao Zhuang, Jinnan Zhang, Yichun He, Nan Wang, Zhuoyue Deng, Lin Mei, Yan Li, Yufei Gao

**Affiliations:** 1 Department of Neurosurgery, China-Japan Union Hospital of Jilin University, Changchun, China; 2 Jilin Province Neuro-oncology Engineering Laboratory, Changchun, China; 3 Jilin Provincial Key Laboratory of Neuro-oncology, Changchun, China; 4 Department of Surgery, University of Louisville, Louisville, KY USA

**Keywords:** CNS cancer, Cell migration, Cell death

## Abstract

Despite advances in therapies, glioblastoma (GBM) recurrence is almost inevitable due to the aggressive growth behavior of GBM cells and drug resistance. Temozolomide (TMZ) is the preferred drug for GBM chemotherapy, however, development of TMZ resistance is over 50% cases in GBM patients. To investigate the mechanism of TMZ resistance and invasive characteristics of GBM, analysis of combined RNA-seq and ChIP-seq was performed in GBM cells in response to TMZ treatment. We found that the PERK/eIF2α/ATF4 signaling was significantly upregulated in the GBM cells with TMZ treatment, while blockage of ATF4 effectively inhibited cell migration and invasion. SPHK1 expression was transcriptionally upregulated by ATF4 in GBM cells in response to TMZ treatment. Blockage of ATF4-SPHK1 signaling attenuated the cellular and molecular events in terms of invasive characteristics and TMZ resistance. In conclusion, GBM cells acquired chemoresistance in response to TMZ treatment via constant ER stress. ATF4 transcriptionally upregulated SPHK1 expression to promote GBM cell aggression and TMZ resistance. The ATF4-SPHK1 signaling in the regulation of the transcription factors of EMT-related genes could be the underlying mechanism contributing to the invasion ability of GBM cells and TMZ resistance. ATF4-SPHK1-targeted therapy could be a potential strategy against TMZ resistance in GBM patients.

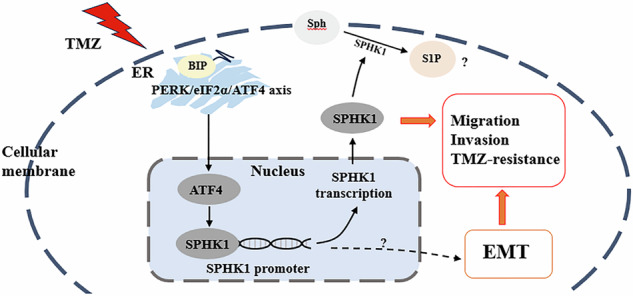

## Highlights

- GBM cells acquired resistance in response to TMZ treatment via constant ER stress.
- ATF4 transcriptionally upregulated SPHK1 expression to promote aggression and TMZ resistance in GBM cells.
- The ATF4-SPHK1 signaling in the regulation of the transcription factors of EMT-related genes could be the potential mechanism contributing to the invasion ability and TMZ resistance in GBM cells.
- ATF4-SPHK1-targeted therapy could be a potential strategy against TMZ resistance in GBM patients.

## Introduction

Despite advances in therapies, the prognosis of glioblastoma (GBM) is very poor with a median survival of 14.6 months and a 5-year survival rate <10% [1]. As the most malignant neuroepithelial tumor [2], GBM recurrence is almost inevitable due to the malignant cells’ aggressive growth and drug resistance. Because the tumor mass has indistinct boundaries and the GBM cells primarily invade into the surrounding brain parenchyma, complete resection is impossible [3]. GBM cells can rapidly recur at the primary site or in the distant regions of the brain [4], while GBM cell movement via epithelial-mesenchymal transition (EMT) is a fundamental cellular event for GBM invasion [5]. Temozolomide (TMZ) is the preferred drug for GBM chemotherapy because it can effectively penetrate the blood-brain barrier and induce cell apoptosis through its DNA methylation damage effects [6]. However, the development of TMZ resistance is quite common, with over 50% of GBM patients do not respond to the TMZ therapy [7]. Acquired resistance of GBM cells in response to TMZ therapy may be the reason for therapeutic failure. Understanding the underlying mechanisms of invasive characteristics and TMZ resistance of GBM is urgently needed.

A study showed that the stability of immunoglobulin heavy chain binding protein (BIP) also known as 78-kDa glucose-regulated protein (GRP78), an endoplasmic reticulum (ER) stress sensor, could regulate EMT to promote GBM invasion [8]. In fact, the unfolded protein response (UPR) is often noticed in GBM cells while UPR has been reported to associate with the invasive characteristics of GBM [9]. Three parallel pathways comprise the UPR signaling via activating transcription factor-6 (ATF6), inositol-requiring enzyme 1α (IRE1α), and PKR-like endoplasmic reticulum kinase (PERK). Highly expressed activating transcription factor 4 (ATF4), a major effector of the PERK pathway, has been found in various cancers [10]. Previously, it was reported that ATF4 mediated development of TMZ resistance via an adaptative manner in human GBM cells [11]. However, it is unknown how the ATF4 signaling and the ATF4-targeting genes contribute to GBM cell invasiveness and TMZ resistance. A accumulating research has uncovered the involvement of ER stress in metabolism of sphingolipids such as to sphingosine-1-phosphate (S1P) [12]. ER is the major site for de novo synthesis and transport of S1P, while sphingosine kinase 1/2 (SPHK1/2) are cytosolic or membrane-associated enzymes that catalyze the ATP-dependent phosphorylation of sphingosine to produce S1P [13]. Highly expressed SPHK1 was associated with the progression and prognosis of GBM [14]. Enhanced invasive ability via SPHK1-EGFR signaling was found in GBM cells [15]. Recent studies have shown that the ATF4 signaling is closely linked to SPHK1/2. For example, SPHK1 deficiency alleviated ER stress by affecting the phosphorylation of inositol-requiring enzyme 1α (IRE1α) and PERK-eukaryotic translation initiation factor 2α (eIF2α) as well as the protein level of ATF4 [16]. A study has shown that overexpression of SPHK1 upregulated the mesenchymal components such as Vimentin, Snail, and N-cadherin, but downregulated the epithelial protein E-cadherin to promote EMT [17]. All these findings suggested that the ATF4 and SPHK1 signaling may play crucial roles contributing to aggressiveness and TMZ resistance of GBM. Exploring the PERK/eIF2α/ATF4-SPHK1 signaling of GBM could provide new insights into the molecular mechanism(s) underlying the invasive characteristics and TMZ resistance.

In this study, the PERK/eIF2α/ATF4-SPHK1 signaling as well as the cellular and molecular events were investigated in GBM cells in response to TMZ treatment. Analysis of integrated RNA-seq and ChIP-seq was performed to determine the ATF4-SPHK1 signaling contributing to aggressiveness and TMZ resistance GBM. An animal GBM model was established to study the TMZ-resistance and the strategies for inhibition of SPHK1 and/or ATF4 in combination of TMZ therapy in mice. We demonstrated that SPHK1 expression was transcriptionally upregulated by ATF4 production in the GBM cells in response to the TMZ treatment. The ATF4-SPHK1 signaling regulated EMT could be an important mechanism to promote GBM invasion and TMZ resistance. Blockage of ATF4-SPHK1 signaling inhibited GBM cell invasion and TMZ resistance in vitro and in vivo, suggesting that TMZ therapy in combination of inhibition of SPHK1-ATF4 signaling could be a therapeutic strategy to overcome TMZ resistance in GBM patients.

## Result

### ER signaling pathways in GBM patients and GBM cells

**Fig. 1:**
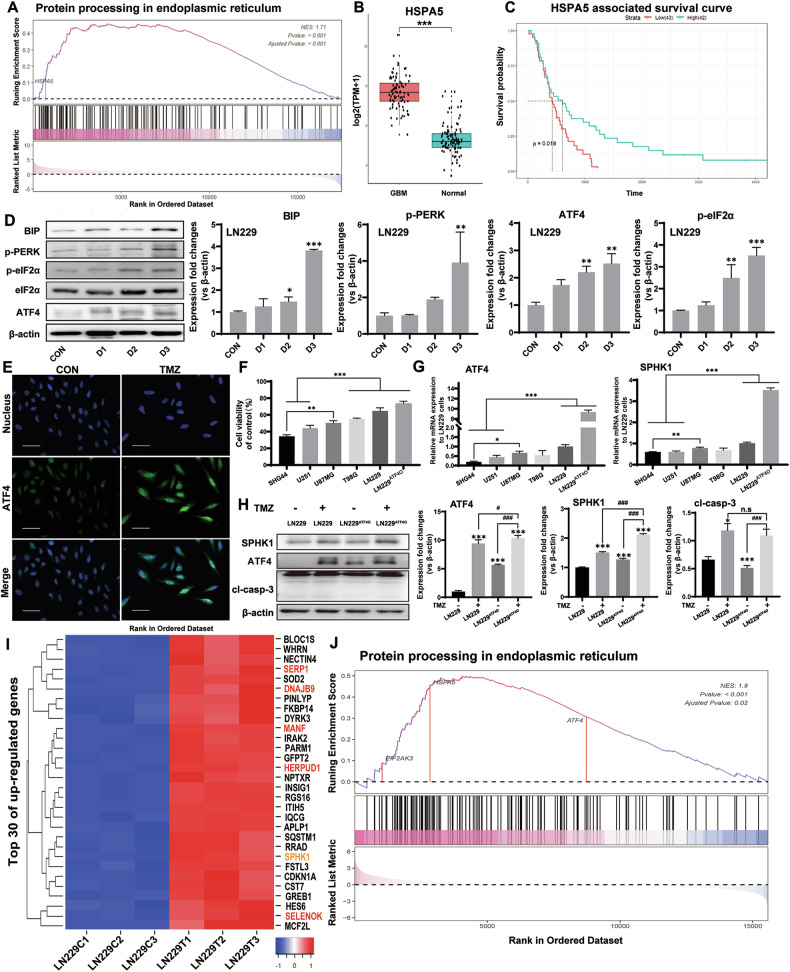
ER signaling pathways in GBM patients and GBM cells. **A** Enriched genes for protein processing in the endoplasmic reticulum by GSEA. **B** HSPA5 mRNA expressions in GBM tissues and cerebral cortex tissues. **C** The survival probability analysis between the GBM patients with HSPA5-high expression and HSPA5-low expression based on the median number of GBM samples. **D** Western blotting analysis of the expression changes of ER stress-related proteins in LN229 cells treated with TMZ for 3 days. **E** Immunofluorescence detection of ATF4 expression and subcellular localization in LN229 cells treated with TMZ for 3 days (bar = 60 μm). **F** Cell viability by MTT in SHG44, U251, U87MG, T98G, LN229, and LN229^ATF4O^ cells with TMZ treatment for 3 days. **G** qPCR to detected the mRNA levels of ATF4, SPHK1, and cleaved caspase-3 in LN229 and LN229^ATF4O^ cells with TMZ treatment for 3 days. **H** Western blotting analysis of the expression changes of SPHK1, ATF4 and cleaved caspase-3 in LN229 and LN229^ATF4O^ cells with TMZ treatment for 3 days. **I** Heatmap of the top 30 upregulated genes (based on Z-score) by RNA-seq analysis of LN229 cells treated with TMZ for 3 days. **J** GSEA analysis of protein processing in endoplasmic reticulum pathway. CON control, D day, cl-casp-3 cleaved caspase-3. (*p < 0.05, **p < 0.01, ***p < 0.001).

To investigate ER stress in GBM, an analysis was first performed using the web-based datasets of RNA-seq data, with 85 primary GBM tissues from the CGGA database and 105 normal cerebral cortex tissues from the GTEx Biobank. In comparison with the normal cerebral cortex tissues, 1238 upregulated genes and 556 downregulated genes (|log2FC| > 2 and *p*-value < 0.05) were found in GBM tissues (Fig. S1A). Enriched genes for protein processing in the endoplasmic reticulum (ER) signaling pathway were found in GBM tissues, e.g., the HSPA5 gene which encodes the binding immunoglobulin protein (BIP) in particular (Fig. 1A). As a regulator for Ca2^+^ homeostasis in ER, BIP is an essential component of translocation machinery for protein import into the ER. Further analysis indicated that HSPA5 gene was significantly upregulated in GBM tissues (Fig. 1B). The survival probability analysis indicated that GBM patients with HSPA5-high expression (versus GBM patients with HSPA5-low expression) showed a poor survival (Fig. 1C). To study ER stress pathway related to chemoresistance in GBM cells, a cell viability assay was performed to determine the time-dependent effects of TMZ treatment in three human GBM cell lines (LN229, U87MG, and T98G cells) (Fig. S1B). A similar dose-dependent effect was found in U87MG cells at 400 µM, T98G cells at 600 µM, and LN229 at 800 µM (Fig. S1C). The apoptotic cells were detected by Flow cytometry. With TMZ treatments (400 µM to treat U87MG cells and 800 µM to treat LN229 cells), the positive cells of mitochondrial membrane potential depolarization and annexin V were significantly increased, further confirming the results of cell viability (Fig. S1D, E). Western blotting analysis showed that the signaling components of BIP, phosphorylated PERK and eIF2α, and ATF4 were significantly upregulated in LN229 and U87MG cells with TMZ treatment up to 3 days (Figs. 1D and S2A). Increased ATF4 protein nuclear localization was further detected by immunofluorescent staining in the TMZ-treated GBM cells (Figs. 1E and S2B). All these results suggested that a pro-adaptive signaling by selective translation of ATF4 could contribute to TMZ resistance. TMZ-resistant cell model was further established by using the high-level drug-resistant GBM cells (LN229, U87MG, and T98G) as well as the LN229 cells transfected with pcDNA3.1-ATF4 overexpression plasmid, named ATF4 overexpression LN229 cells (LN229^ATF4O^ cells). Two more cell lines, SHG44 and U251, were selected as controls because they were widely accepted as the low-level drug-resistant glioblastoma cells [18]. All these cells were treated with TMZ at 200 µM, 400 µM, and 600 µM for 3 days. With 400 µM TMZ treatment, the U87MG, T98G, LN229, and LN229^ATF4O^ cells showed high TMZ-resistant (cell viability over 50%) compared to the SHG44 and U251 cells (cell viability under 50%) (Fig. 1F). The qPCR results showed significant increases of mRNA levels of ATF4 and SPHK1 in U87MG, LN229, and LN229^ATF4O^ cells but not in SHG44 and U251 (Fig. 1G). Interestingly, the mRNA expressions of ATF4 and SPHK1 in T98G cells were not significantly updated by 400 µM TMZ treatment, suggesting a different TMZ-resistant mechanism of T98G cells from U87MG and LN229 cells. To exclude possibility that TMZ kills cells by directly activating ATF4, we performed a study using the LN229 cells and LN229^ATF4O^ cells, significantly increased protein levels of ATF4 and SPHK1 were induced by TMZ in either LN229 cells or LN229^ATF4O^ cells, however, the LN229^ATF4O^ cells without TMZ treatment did not showed a significant increase of cleaved caspase-3 (Fig. 1H), confirming the pro-adaptive signaling for TMZ resistance by selective translation of ATF4 and its downstream target gene such as SPHK1, other than directly activating ATF4. Based on the results of cell viability and Flow cytometry, LN229 cells with 400 µM TMZ treatment for 3 days was selected for RNA-seq analysis. The results showed 831 upregulated genes and 697 downregulated genes in the TMZ-treated group compared to untreated control group (|log2FC| > 1 and *p*-value < 0.05) (Fig. S1F). Among the top 30 upregulated genes, 5 ER stress-associated genes including *DNAJB9*, *HERPUD1*, *SELENOK*, *SERP1*, and *MANF* (Fig. 1I, highlighted in red) and *SPHK1* gene (Fig. 1I, highlighted in brown) were found. Based on previous reports, these 5 genes are primarily involved in protein folding, ER-associated degradation (ERAD), and calcium homeostasis regulation within the ER [19–23]. Gene Set Enrichment Analysis (GSEA) indicated that an ER protein processing pathway was noticed among the top 4 signaling pathways (Fig. S1G), while the UPR protein processing-associated genes including HSPA5, EIF2AK3 and ATF4 were enriched and upregulated in this ER protein processing pathway (Fig. 1J). Taken together, ER stress, particularly the PERK branch of the UPR, plays a critical role in contribution to aggressive growth and TMZ resistance in GBM tumor. It is important to have of a more refined understanding of the ATF4-SPHK1 signals contributing to the aggressive behavior and TMZ resistance for therapeutic failure, resulting in a poor prognosis in GBM patients.

### PERK/eIF2α/ATF4 signaling promotes GBM cell aggression and TMZ resistance

**Fig. 2:**
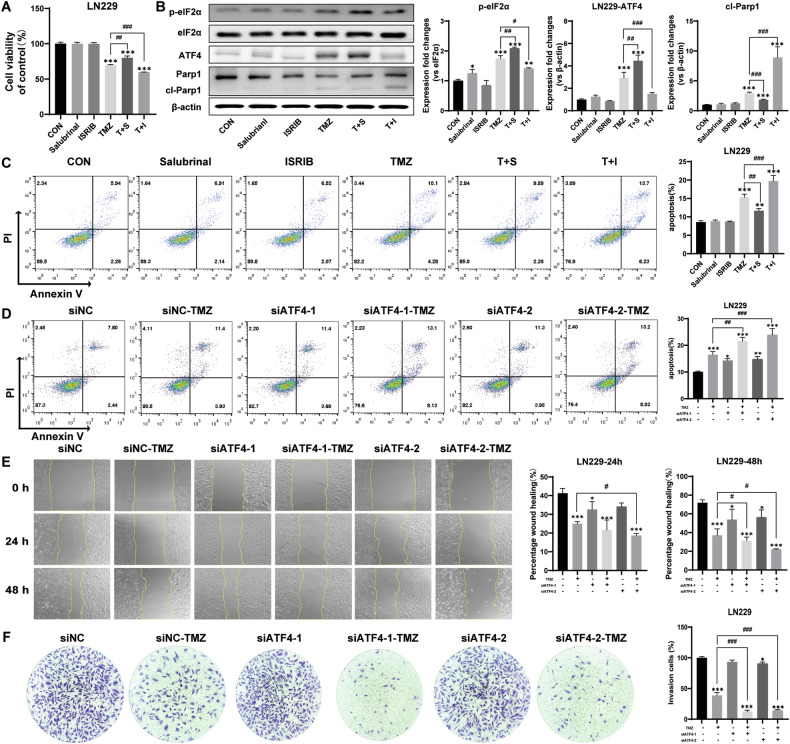
PERK/eIF2α/ATF4 signaling promotes GBM cell aggression and TMZ resistance. **A** Cell viability by MTT assay in LN229 cells with treatments of TMZ as well as salubrinal and ISRIB. **B** Western blotting analysis of the protein expression of eIF2α/ATF4 signaling and apoptotic effector in LN229 cells with treatments of TMZ as well as salubrinal and ISRIB. **C** Flow cytometry detected cell apoptosis in LN229 cells with treatments of TMZ as well as salubrinal and ISRIB. **D** Flow cytometry detected cell apoptosis in LN229-siATF4-1 cells, LN229-siATF4-2 cells, and LN229 cells with TMZ treatment. **E** Wound healing assay detected the migration of LN229-siATF4-1 cells, LN229-siATF4-2 cells, and LN229 cells treated with TMZ for 24 h and 48 h under serum-free culture conditions. **F** Trans-well assay to detect the invasion ability of LN229-siATF4-1 cells, LN229-siATF4-2 cells, and LN229 cells treated with TMZ for 12 h. T TMZ, S salubrinal, I ISRIB. (*p < 0.05, **p < 0.01, ***p < 0.001; #*p* < 0.05, ##*p* < 0.01, ###*p* < 0.001).

In fact, ATF4 has been well-accepted as a stress-induced transcription factor to selectively upregulate the downstream genes for cancer progression and drug resistance [24]. To study the eIF2α/ATF4 signaling in contribution to TMZ resistance, the cellular events including cell viability, apoptosis, migration, and invasion were investigated in LN229 and U87MG with TMZ treatment. Salubrinal was to enhance eIF2α phosphorylation. ISRIB was used to prevent the formation of stress granules triggered by eIF2α phosphorylation. As expected, the TMZ-induced cell death was significantly attenuated by salubrinal but amplified by ISRIB (Figs. 2A and S2C). Decreased/increased protein levels of p-eIF2α and ATF4 along with increased/decreased protein level of cleaved Parp1 were detected by western blotting analysis (Figs. 2B and S2D), indicating that blockage of eIF2α/ATF4 signaling caused increase of apoptotic effector and vice versa. Consistently, the TMZ induced apoptosis was significantly attenuated by salubrinal but amplified by ISRIB (Figs. 2C and  S2E). Three siRNA sequences (siATF4-1, siATF4-2, and siATF4-3) were used to silence the ATF4 gene of LN229 cells and U87MG cells. The results showed that all three siATF4 sequences could significantly down-regulate the expression of ATF4 protein in both U87MG and LN229 cells (Fig. S3A). siATF4-1 and siATF4-2 were selected for ATF4 gene silencing in LN229 cells and U87MG cells with TMZ treatment for 3 days. The results showed that ATF4 gene silencing significantly attenuated the TMZ resistance, as evidenced by decreases in GBM cell viability (Fig. S3B) and increases in apoptotic GBM cells with TMZ treatment (Figs. 2D and S3C). The cellular events of migration and invasion were further investigated by wound healing assay and trans-well invasion assay. The results showed that ATF4 gene silencing effectively inhibited cell migration and invasion in both the LN229 cells (Fig. 2E, F) and the U87MG cells (Fig. S3D, E). All these findings suggested that PERK/eIF2α/ATF4 signaling contributed to GBM cell aggression and TMZ resistance. Therefore, it is critical to further explore the ATF4-targeting genes to elucidate the mechanism underlying the aggressiveness and TMZ resistance in GBM.

### SPHK1 is among the top ATF4 target genes being enriched in cell migration pathways

**Fig. 3:**
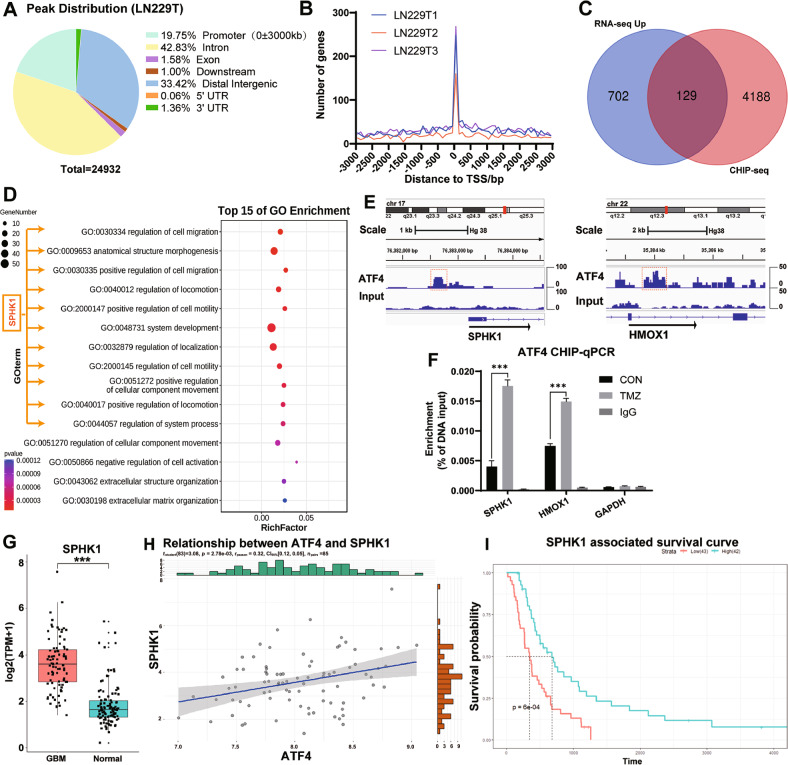
SPHK1 expression is transcriptionally upregulated by ATF4 production in GBM cells. **A** CHIP-seq analysis of ATF4 binding sites in the genome of LN229 cells after TMZ treatment for 3 days. **B** Distribution of ATF4-enriched DNA fragments around TSS ± 3000 bp. **C** Venn diagram showing the relationship between upregulated genes in RNA-seq and genes with ATF4 binding in the promoter region in CHIP-seq. **D** GO enrichment analysis of upregulated genes in the promoter region that contain ATF4 binding sites. **E** IGV analysis for ATF4 binding in the promoter region of SPHK1 and HMOX1 genes. **F** CHIP-qPCR to detect the binding capacity of ATF4 to the promoter regions of SPHK1, HMOX1, and GAPDH in LN229 cells with TMZ treatment for 3 days. **G** SPHK1 mRNA expressions in GBM tissues and cerebral cortex tissues. **H** Correlation between SPHK1 gene expression and ATF4 gene expression in GBM. **I** Survival probability analysis between the GBM patients with SPHK1-high expression and SPHK1-low expression based on the median number of GBM samples. (***p < 0.001).

As a number of target genes of ATF4 could be involved in the cell migration and chemoresistance in GBM, we performed ChIP-seq analysis using LN229 cells to explore the gene network being transcriptionally regulated by ATF4. With TMZ treatment for 3 days, 24,932 gene segments were bound by ATF4 and 4,924 gene segments were located at the promoter region, accounting for 19.75% of the total genes (Fig. 3A). The DNA segments in the promoter region were predominantly located near the transcription start site (TSS) about 3000 bp (Fig. 3B). An integrated analysis of ChIP-Seq and RNA-Seq showed a total of 129 upregulated genes with ATF4 binding at their promoter regions (Fig. 3C). The directed acyclic graph (DAG) of the GO enrichment analysis demonstrated a strong association between these genes and the biological process of positive regulation of cell migration (Fig. S4). Gene Ontology (GO) enrichment analysis indicated that these ATF4-targeting genes were mainly enriched in biological processing pathways related to regulation of cell migration, anatomical structure morphogenesis, regulation of locomotion, and positive regulation of cellular motion, while the SPHK1 gene was noticed in 11 out of 15 migration related pathways (Fig. 3D). It should be noted that SPHK1 gene and other 5 ER stress-associated genes were simultaneously upregulated among the top 30 genes in the aforementioned RNA-seq analysis, (Fig.1D, highlighted in brown). Previous studies have shown that SPHK1 can promote proliferation and invasion in GBM cells [25] while inhibition of SPHK1 can induce apoptosis, suppress growth [26], and enhance the sensitivity of GBM cells to TMZ [27]. Given these important findings, the ATF4-SPHK1 signaling was therefore chosen for further investigation. By using Integrative Genomics Viewer (IGV) to generate ChIP-seq signals, significantly binding peaks of ATF4 were found in the promoter region of SPHK1 and heme oxygenase 1 (HMOX1) (Fig. 3E), a well-known transcriptional target of ATF4 [28]. By using HMOX1 gene as a positive control and GAPDH gene as a negative control, a CHIP-qPCR assay was performed to validate the CHIP-seq results. ATF4-enriched DNA fragments of both SPHK1 promoter and HMOX1 promoter were detected and significantly enhanced by TMZ treatment (Fig. 3F). The RNA-seq analysis (CGGA database and GTEx Biobank) showed that SPHK1 gene was significantly upregulated in GBM compared to normal brain cortex tissue (Fig. 3G). A positive correlation between the gene expression levels of ATF4 and SPHK1 was found in GBM patients (Fig. 3H). Further analysis for survival probability indicated that GBM patients with SPHK1-high expression (versus GBM patients with SPHK1-low expression) showed poor survival (Fig. 3I). Taken together, all these findings suggested that SPHK1, as an ATF4-targeting gene, could play a critical role for GBM cell migration and TMZ resistance.

### Upregulated ATF4-SPHK1 signaling in GBM cell in response to TMZ

**Fig. 4:**
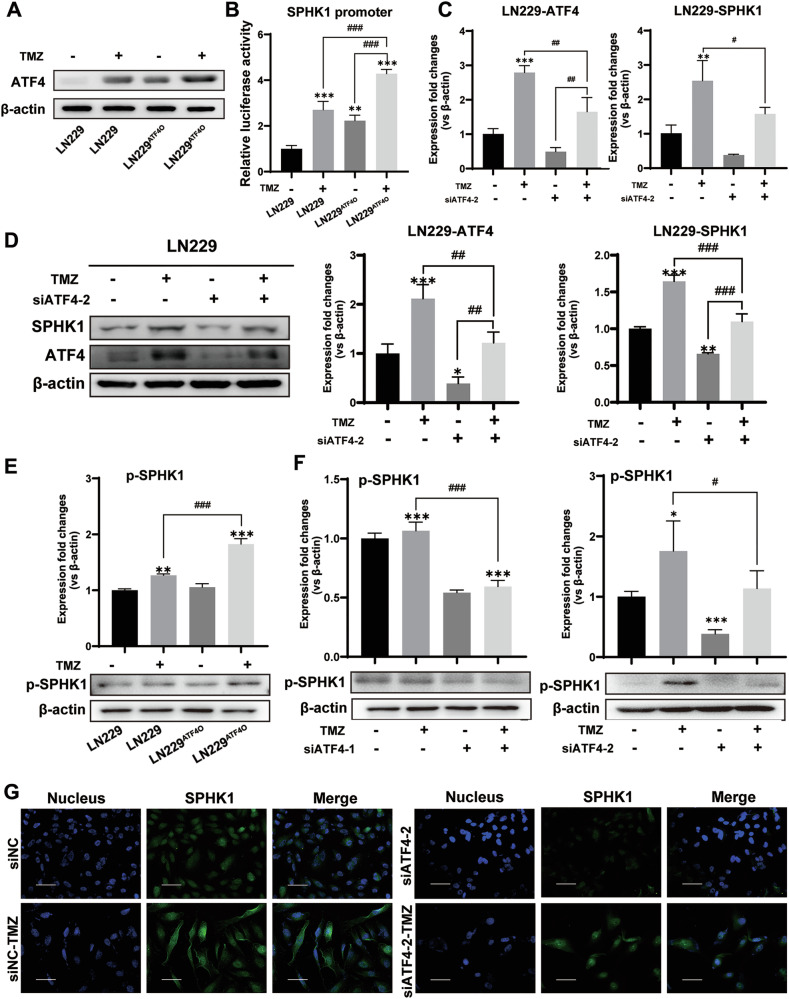
Upregulated ATF4-SPHK1 signaling in GBM cell in response to TMZ. **A** Protein levels of ATF4 by western blotting analysis in LN229 and LN229^ATF4O^ cells with TMZ treatment for 3 days. **B** Analysis of SPHK1 promoter activity using a dual-luciferase assay in LN229 and LN229^ATF4O^ cells with TMZ treatment for 3 days. **C** mRNA levels of SPHK1 and ATF4 by qPCR in LN229-siATF4-2 cells and LN229 cells with TMZ treatment for 3 days. **D** Protein levels of SPHK1 and ATF4 by western blotting analysis in LN229-siATF4-2 cells and LN229 cells with TMZ treatment. **E** Protein levels of phosphorylated SPHK1 by western blotting analysis in LN229 and LN229^ATF4O^ cells with TMZ treatment for 3 days. **F** Protein levels of phosphorylated SPHK1 by western blotting analysis in LN229-siATF4-1 cells, LN229-siATF4-2 cells, and LN229 cells with TMZ treatment for 3 days. **G** Immunofluorescent staining detection of SPHK1 expression and subcellular localization in LN229-siATF4-2 cells and LN229 cells treated with TMZ for 3 days (bar = 60 μm). (*p < 0.05, **p < 0.01, ***p < 0.001; #*p* < 0.05, ##*p* < 0.01, ###*p* < 0.001).

The LN229^ATF4O^ cells were used to study whether ATF4-SPHK1 signaling could be a reinforcing response to ATF4 production under TMZ stress. Significantly increased ATF4 protein level was found in the LN229^ATF4O^ cells compared to LN229 cells, while ATF4 protein level was further elevated in the LN229^ATF4O^ cells with TMZ treatment (Fig. 4A). A dual-luciferase reporter assay was performed in The LN229^ATF4O^ cells and LN229 cells with TMZ treatment. Significantly increased luciferase activity was found in either The LN229^ATF4O^ cells or TMZ-treated LN229 (Fig. 4B), suggesting that SPHK1 expression could be enhanced by either the overexpressed ATF4 or the TMZ-induced increase of ATF4 production. By using siATF4-2 to silence ATF4 expression, ATF4-SPHK1 signaling was further validated in both LN229 cells and U87MG cells in response to TMZ treatment. As expected, silencing ATF4 gene significantly reduced not only ATF4 mRNA level but also SPHK1 mRNA level in these two GBM cells (Figs. 4C and S5A). Consistently, the TMZ-induced increases of ATF4 and SPHK1 protein levels were attenuated in both LN229 and U87MG cells by ATF4 gene silencing (Fig. 4D and Fig. S5B). SPHK1 is primarily located in the cytoplasm and needs to be phosphorylated and translocated to the cell membrane to exert its catalytic function in the production of S1P [29]. The phosphorylated SPHK1 was therefore studied in LN229^ATF4O^ cells and LN229 cells. Significant increases of phosphorylated SPHK1 were found in the LN229^ATF4O^ cells compared to LN229 cells, while phosphorylated SPHK1 was further elevated in the LN229^ATF4O^ cells with TMZ treatment (Fig. 4E). In contrast, the phosphorylated SPHK1 levels were downregulated in the LN229 cells with ATF4 gene silencing (Fig. 4E). To elucidate the translocation of cytoplasm SPHK1 to the membrane in response to TMZ treatment, an immunofluorescent assay was performed to detect the localization of SPHK1 in both LN229-siATF4-2 cells and U87MG-siATF4-2 cells as well as the control cells (siNCs). Positive fluorescent staining and cell membrane localization of SPHK1 were detected in the siNCs with TMZ treatment, but very weak fluorescent staining was detected in either siNCs without TMZ treatment or both LN229-siATF4-2 cells and U87MG-siATF4-2 cells with/without TMZ treatment (Figs. 4G and S5C). Taken together, all these results demonstrated that SPHK1 expression was transcriptionally upregulated by ATF4 production in the GBM cells in response to the TMZ treatment.

### Targeting SPHK1 inhibits GBM cell aggression and TMZ resistance

**Fig. 5:**
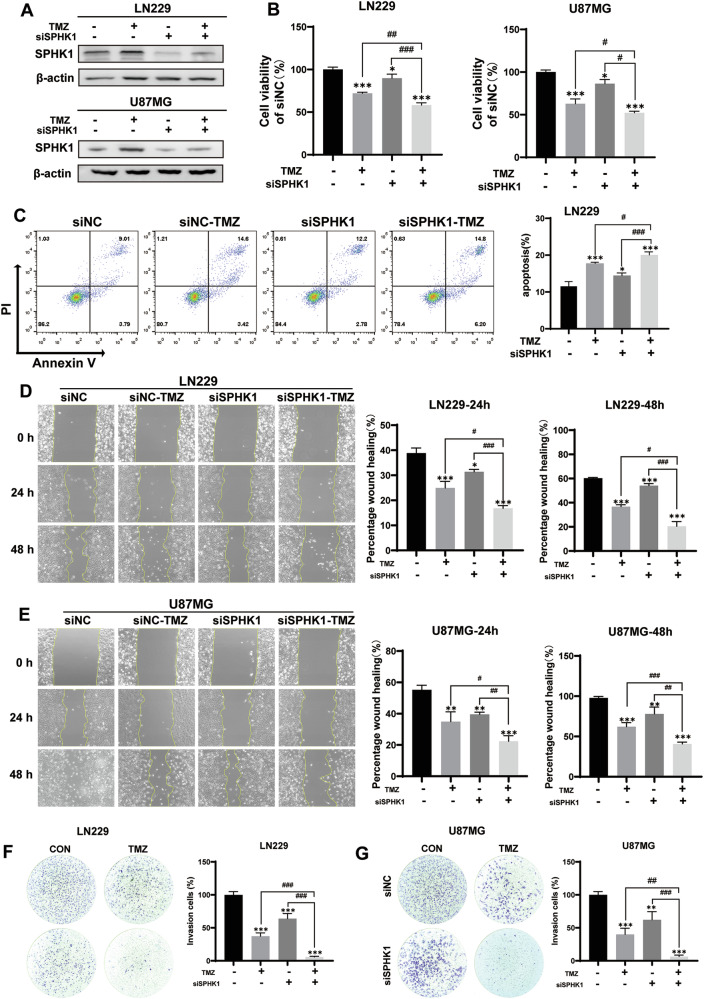
Targeting SPHK1 inhibits GBM cell aggression and TMZ resistance. **A** Western blotting analysis for silencing SPHK1 in LN229 and U87MG cells. **B** MTT assay to detect cell viability in LN229 and U87MG cells with TMZ treatment for 3 days. **C** Flow cytometry detected apoptosis in LN229 cells with TMZ treatment for 3 days. **D**, **E** Wound healing assay detected the migration of LN229 and U87MG cells treated with TMZ for 24 h and 48 h under serum-free culture conditions. **F**, **G** Trans-well assay to detect the invasion ability of LN229 and U87MG cells treated with TMZ for 12 h. (*p < 0.05, **p < 0.01, ***p < 0.001; #*p* < 0.05, ##*p* < 0.01, ###*p* < 0.001).

To further study the effect of SPHK1 on GBM cell aggression and TMZ resistance, we used a siRNA sequence to silence SPHK1 in both LN229 cells and U87MG cells. Significantly decreased SPHK1 protein levels were found in the LN229 cells and U87MG when the SPHK1 gene was silenced. The LN229-siSPHK1 cells and U87MG-siSPHK1 cells were treated with TMZ for 3 days to perform MTT assay and Flow cytometry for detection of cell viability and apoptosis. The results showed that silencing SPHK1 gene significantly decreased cell viability (Fig. 5B) and increased apoptotic index in GBM cells with TMZ treatment (Fig. 5C), suggesting that silencing SPHK1 gene in both LN229 cells and U87MG cells increased the efficacy of TMZ killing and sensitivity of TMZ treatment. The cellular events of migration and invasion were further investigated by wound healing assay and trans-well invasion assay. The results showed that silencing SPHK1 gene effectively inhibited cell migration (Fig. 5D, E) and invasion (Fig. 5F, G) in both U87MG and LN229 cells with TMZ treatment. All these results suggested that SPHK1 played a critical role for GBM cell to acquire the ability of migration/envision and TMZ resistance. Targeting SPHK1 could be a promising strategy to improve the therapeutic efficacy of TMZ in GBM patients.

### ATF4-SPHK1 signaling regulated EMT in GBM

**Fig. 6:**
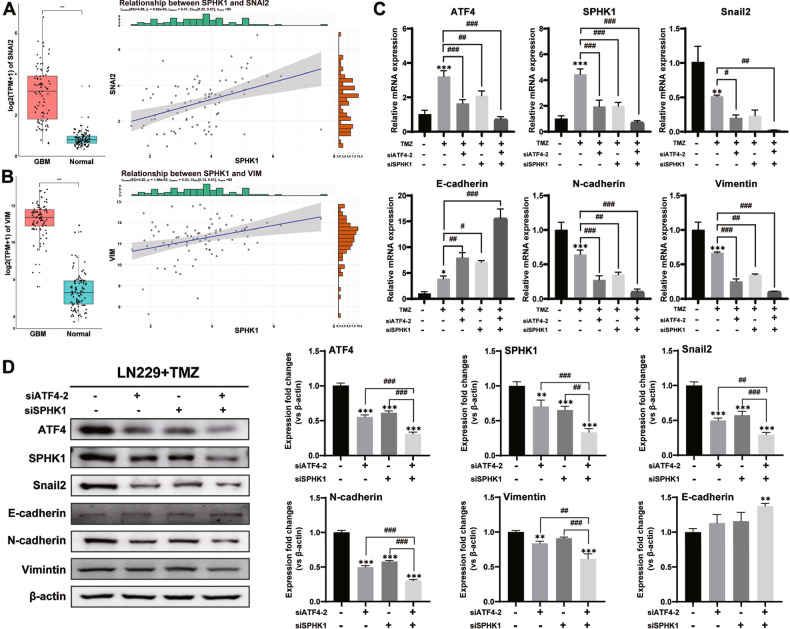
ATF4-SPHK1 signaling regulated EMT in GBM. **A**, **B** SNAI2 and VIM mRNA expressions in GBM tissues and cerebral cortex tissues and correlation between SPHK1 gene expression and SNAI2/VIM mRNA gene expressions in GBM tissues. **C** mRNA levels of SPHK1, ATF4, Snail2, N-cadherin, vimentin, and E-cadherin by qPCR in LN229 cells, LN229-siATF4-2 cells, LN229-siSPHK1 cells, and LN229-siATF4-2/siSPHK1 cells with TMZ treatment for 3 days. **D** Protein levels of SPHK1, ATF4 Snail2, N-cadherin, vimentin, and E-cadherin by western blotting analysis in LN229 cells, LN229-siATF4-2 cells, LN229-siSPHK1 cells, and LN229-siATF4-2/siSPHK1 cells with TMZ treatment for 3 days. (*p < 0.05, **p < 0.01, ***p < 0.001; #*p* < 0.05, ##*p* < 0.01, ###*p* < 0.001).

EMT has been widely accepted as a cellular event contributing to tumor invasion and chemotherapy resistance [30]. The EMT-related genes in various human cancers (breast cancer, gastric cancer, colon cancer, lung cancer) have been found to be associated with the PERK-UPR pathway, rather than the other two pathways of UPR [31]. As EMT is regulated by several transcription factors such as the protein products of the SNAI gene family, which inhibits the expression of epithelial genes [32], we speculate that ATF4-SPHK1 signaling promotes GBM invasion and TMZ resistance via regulating the EMT event. To investigate the SPHK1 signaling related to ER stress and EMT in GBM, the RNA-seq data from web-based dataset (CGGA325) were further analyzed. Based on the median number of GBM samples, two groups were assigned to represent the SPHK1 expression levels (high, low). Analysis of DEGs indicated 265 upregulated genes and 101 downregulated genes (|log2FC| > 2 and *p*-value < 0.05) in SPHK1-high group in comparison with the SPHK1-low group (Fig. S6A). Single-gene GSEA indicated that the high expression of SPHK1 was linked to pathways of protein processing in endoplasmic reticulum and EMT signaling. The enriched genes of SNAI2, Vimentin (VIM), and CDH2 were noticed in EMT signaling pathway (Fig. S6B, C). Further analysis indicated that significantly upregulated expressions of SNAI2 and VIM in GBM patients, while a positive correlation was found between SPHK1 and SNAI2/VIM in GBM patients (Fig. 6A, B). Two aforementioned siRNAs (siSPHK1 and siATF4-2) were used to investigate whether blockage of ATF4-SPHK1 signaling could affect the EMT events. The qPCR results indicated that silencing either ATF4/SPHK1 or both significantly downregulated the mRNA expressions of the transcription factor Snail2 and the expressions of mesenchymal-related genes (Vimentin and N-cadherin) but upregulated mRNA expression of epidermal-related gene E-cadherin in the LN229 cells (Fig. 6C). The protein expressions of the transcription factor Snail2 and the EMT components by western blotting analysis were found in consistent to the mRNA expressions (Figs. S7A and 6D). These results demonstrated that blockage of ATF4-SPHK1 signaling attenuated the deleterious molecular event of EMT, confirming the critical role of ATF4-SPHK1 signaling in GBM aggressive growth and GBM TMZ resistance.

### Blockage of ATF4-SPHK1 signaling suppressed tumor growth in mice with TMZ therapy

**Fig. 7:**
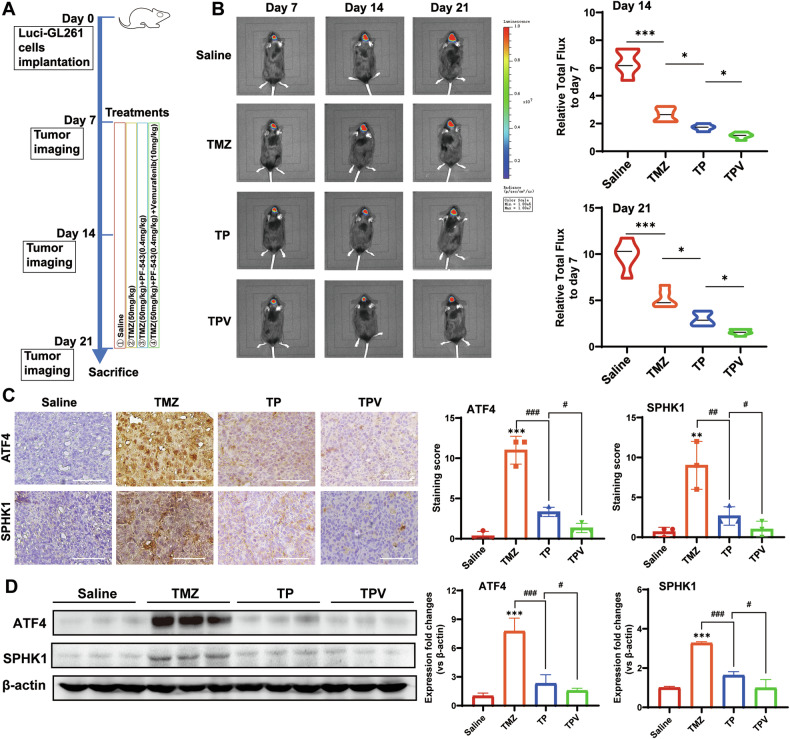
Blockage of ATF4-SPHK1 signaling suppressed tumor growth in mice with TMZ therapy. **A** Workflow of glioma orthotopic mouse model and treatments (*n* = 6). **B** Representative bioluminescence images (BLI) at day 7, day 14, and day 21 and the relative flux value (versus day 7) as the indexes of tumor growth in 4 groups of mice. **C** Representative images of IHC staining for ATF4 and SPHK1 and IHC scoring as the indexes of protein expression in 4 groups of mice (bar = 90 μm). **D** Protein levels of ATF4 and SPHK1 by western blotting analysis in 4 groups of mice model group. TP: TMZ + PF-543; TPV: TMZ + PF-543 + Vemurafenib. (*p < 0.05, **p < 0.01, ***p < 0.001; #*p* < 0.05, ##*p* < 0.01, ###*p* < 0.001).

In the established GBM mouse model, localization of tumor mass was verified by bioluminescence imaging in mouse brain 7 days after implantation of Luci-GL261 cells, indicating successful establishment of the in vivo GBM model (Fig. 7A). Decreased bodyweight was found in the treatment arms versus saline controls, but no statistical significance (Fig. S7B). Macroscopic observation was performed to further confirm the tumor formation. As shown in Fig. 7A, six mice in each group were treated with TMZ, TMZ + PF-543, TMZ + PF-543 + Vemurafenib, and saline, respectively. The combination of TMZ and PF-543 as well as combination of TMZ and PF-543 and vemurafenib showed significantly improved tumor suppression, compared to TMZ treatment only. Notably, TMZ + PF-543 + Vemurafenib treatment arm showed the highest efficacy for tumor suppression compared to the other two treatment arms and saline controls (Fig. 7B). Immunohistochemical staining of ATF4 and SPHK1 was further studied in the micro-sections of tumor tissues. The increased indexes of ATF4 and SPHK1 expression in TMZ mice were significantly attenuated in the mice with TMZ + PF-543 treatment or with TMZ + PF-543 + Vemurafenib treatment (Fig. 7C). The result of immunohistochemical staining was further confirmed by western blotting analysis (Fig. 7D). Taken together, TMZ therapy in combination with inhibition of ATF4-SPHK1 signaling could improve the therapeutic efficacy for tumor suppression in the TMZ-resistant GBM mice.

## Discussion

Emerging evidence implies the important role of ER stress-mediated aggressiveness and TMZ resistance. In this study, we found that ATF4 transcriptionally upregulated SPHK1 expression while the ATF4-SPHK1 signaling played a critical role in the regulation of EMT-related genes. Inhibition of ATF4 prevented the migration and invasion abilities of GBM cells but enhanced cell apoptosis by TMZ treatment. Our data demonstrated that GBM cells acquired resistance in response to TMZ treatment via constant ER stress. We reported, for the first time, the ATF4-SPHK1 signaling could be the potential mechanism contributing to the invasion ability of GBM cells and TMZ resistance.

ER stress has been widely accepted as one of the most important hallmarks in a variety of cancers [33]. Due to rapid growth with a large amount of protein synthesis, hypoxia condition, and exposure to chemotherapeutic drugs, UPR was often noticed in GBM cells with aggressiveness and high-level resistance to chemotherapy [9]. In this study, analysis of RNA-seq data of GBM from CGGA database showed that HSPA5 gene (encoding BIP), the master regulator of the ER stress response, was highly upregulated in GBM patients. Significantly increased protein levels of BIP, phosphorylated PERK/eIF2α, and ATF4 were found in the TMZ-resistant GBM cells. Consistent with previous studies showing that knockdown of BIP expression could inhibit GBM cell proliferation and increase TMZ sensitivity [34], our data further supported that inhibition of eIF2α phosphorylation or silencing ATF4 expression effectively inhibited cell viability, cell migration and invasion, and promoted cell apoptosis in the TMZ-resistant GBM cells. As a stress-induced transcription factor, ATF4 controls the expression of a wide range of adaptive genes which allow cancer cells to endure periods of stress [35]. Previous studies indicated that ATF4 signaling was involved in the regulation of tumor cell migration and invasion. For example, a study showed that ATF4 regulated the Wnt/β-catenin signaling pathway to promote the proliferation and invasion of lung cancer cells [36]. In another study, ATF4 upregulated the expression of LAMP3 to promote cell migration in breast cancer cells under hypoxic conditions [37]. In agreement with these previous studies, our data showed that TMZ treatment caused upregulation of PERK/eIF2α/ATF4 signaling in GBM cells, while inhibition of ATF4 in TMZ-resistant GBM cells not only prevented the migration and invasion abilities but also enhanced cell apoptosis. We speculated that TMZ could induce a pro-adaptive PERK/eIF2α/ATF4 signaling by selective translation of ATF4 to transcriptionally regulate the genes for cell migration and chemoresistance in GBM cells. Therefore, we further analyzed the ATF4-targeting genes involved in the cell migration and chemoresistance in GBM cells. GO enrichment analysis indicated that these ATF4-targeting genes were enriched in the cell migration pathways, while the SPHK1 gene was noticed in 11 out of 15 migration-related pathways.

SPHK1 has been reported to have a significant impact on the carcinogenetic processes including progression, migration, and invasion [38]. A recent work reports that SPHK1 is upregulated in GBM patients and positively correlated with poor prognosis [25]. Overexpression of SPHK1 promotes proliferation and invasion in GBM cells [25]. Targeting SPHK1 by SPHK1 inhibitors (SK1-I or SK1-II) can induce apoptosis, suppress the growth of GBM cells and GBM xenografts [26], and enhance the sensitivity of GBM cells to TMZ and Gy X-ray [27]. In this study, we further demonstrated that SPHK1 expression was transcriptionally upregulated by ATF4 in the GBM cells in response to the TMZ treatment. Silencing ATF4/SPHK1 signaling re-established sensitivity of GBM cells to TMZ treatment, as evidenced by induction of apoptosis and inhibition of migration and invasion in the GBM cells. It is well known that EMT is the key molecular event for tumor cells to become invasive and drug resistant. Analysis of the RNA-seq data showed that the genes of SNAI2, VIM, and CDH2 were enriched in EMT signaling pathway and SNAI2 and VIM were significantly upregulated in GBM patients. Silencing ATF4/SPHK1 signaling down-regulated the expression of Snail2, N-cadherin, and Vimentin, and upregulate the expression of E-cadherin in GBM cells. All these data support that ATF4-SPHK1 signaling is strongly associated with EMT contributing to the invasion ability and TMZ resistance of GBM cells. Our findings are supported by the following previous studies: (1) the SPHK1 /S1P signaling promotes EMT in breast cancer and colon cancer [32, 39]; (2) EMT cells constitutively activate the PERK–eIF2α–ATF4 signaling for them to invade/metastasize and form tumor-spheres [31], (3) knockdown of SPHK1 inhibits the phosphorylation of IRE1α and PERK, reduces ATF4 expression and ATF6 activation, alleviates ER stress in mice [16], and **4**) expression of the EMT transcription factor Snail2 is depended on ATF4 production in the process of EMT caused by glutamine deficiency in pancreatic cancer [40]. In the animal study, it is further demonstrated that inhibition of SPHK1 by PF-543 (a SPHK1 inhibitor) and/or inhibition of ATF4 by vemurafenib (a BRAF inhibitor to reduce ATF4 production) can inhibit the growth of TMZ-resistant GBM tumor. The major limitation of this study is lack of a transgenic model to elucidate the molecular mechanisms. In this regard, the remaining to be studied in future is to clarify the important issues e.g. the ATF4-SPHK1 axis regulations of EMT-related genes contributing to TMZ-resistant GBM tumor.

In conclusion, GBM cells acquired chemoresistance in response to TMZ treatment via constant ER stress. ATF4 transcriptionally upregulated SPHK1 expression to promote GBM cell aggression and TMZ resistance. The ATF4-SPHK1 signaling in regulation of the transcription factors of EMT-related genes could be the potential mechanism contributing to the invasion ability of GBM cells and TMZ resistance. ATF4-SPHK1-targeted therapy could be a potentially effective strategy against TMZ resistance in GBM patients.

## Materials and methods

### Cell culture and treatments

All cell lines were identified by STR DNA profiling and proved to be mycoplasma-free by Myco-Lumi™ Luminescent Mycoplasma Detection Kit (Beyotime Institute of Biotechnology, Shanghai, China, #C0298M). The human GBM cell lines SHG44, U251, U87MG, LN229, T98G cells and mouse glioma cell line Luci-GL261 cells were purchased from Gugangzhou Cellcook Biotech Co., Ltd (Guangzhou, China). All cells were cultured in DMEM high glucose medium (Gibco Life Technologies, Carlsbad, CA, USA) containing 10% fetal bovine serum (Invitrogen), 100 IU/mL streptomycin and 100 IU/mL penicillin under 5% CO_2_ at 37 °C. The GBM cells were treated with to TMZ (MedChemExpress, Monmouth Junction, NJ, USA, #HY-17364) at respective concentrations and time points. The GBM cells were exposed to 5 μM Salubrinal (MedChemExpress, #HY-15486) and 400 nM ISRIB (MedChemExpress, #HY-12495).

### Bioinformatics analysis

For gene expression between GBM and normal brain tissue, gene transcriptome data of GBM (CGGA325) was downloaded from Chinese Glioma Genome Atlas (CGGA) database (http://www.cgga.org.cn/) [41] and gene transcriptome data of cerebral cortex was downloaded from Genotype-Tissue Expression (GTEx) Biobank (https://gtexportal.org/home/). Differential expression genes were characterized by using limma (v3.54.2) with expression difference fold |log2(FoldChange)| > 2 and *p*-value < 0.05. For survival analysis, the samples of GBM (CGGA325) in CGGA database were sorted according to the expression of targeted genes. Then the samples were divided into high expression group (42) and low expression group (43) based on median number. Survival analysis was performed by using survival (v3.5-5) and survminer (0.4.9).

### RNA-seq and analysis

Total RNA was obtained and reverse transcribed into cDNA. The conduction of sequencing was on Illumina HiSeq platform by Personal Biotechnology company (Shanghai, China). Aligning reads of each sample to the human genome (Homo sapiens. GRCh38) was performed. Differential expression genes were characterized by using the DESeq2 with expression difference fold |log2FoldChange| > 1 and *p*-value < 0.05. RNA-seq data was upload to the NCBI (BioProject: PRJNA1122393).

### Chromatin immunoprecipitation

Chip was completed by using the ChIP Assay Kit (Beyotime, #P2078). After treated with TMZ for 3 day, LN229 cells were crosslinked in culture medium with 1% formaldehyde for 10 min at 37 °C. Then, the cells were incubated with 0.125 M glycine to terminate crosslink. After washed with PBS containing 1 mM PMSF for three times, cells were resuspended in lysis buffer and sonicated to fragment DNA. After centrifugation, the supernatant was immuno-precipitated with anti-ATF4 or IgG (as a negative control) antibodies. DNA was pulled down with Protein A + G beads and purified from DNA-protein complex for sequencing and chromatin immunoprecipitation (CHIP)-qPCR.

### CHIP-seq and analysis

After CHIP DNA was purified, the conduction of sequencing was on Illumina HiSeq platform by Personal Biotechnology company (Shanghai, China). Reads were mapping to human (Homo sapiens. GRCh38) genome and carried out by Bowtie2 software, and unique mapping reads were selected for the downstream analysis. Homer2 was used to call peaks, and CHIP seeker was used for peak annotation. Promoters were defined as 3 kb from TSS. CHIP-seq data was upload to the NCBI (BioProject: PRJNA1122481).

### Dual-luciferase reporter assay

pcDNA3.1-ATF4 overexpression plasmid, pGL3-SPHK1 promoter (76382516- 76382941) -firefly luciferase reporter plasmid and Renilla luciferase reporter plasmid (RL-TK) were purchased from GenePharma Bio Co. Ltd. (Shanghai, China). These plasmids were transfected to LN229 cells using TurboFect Transfetion Reagent (R0531, Thermo Fisher Scientific, MA, USA). After being treated with TMZ for 3 days, Dual-Luciferase Reporter Assay was performed as the manufacturer of Dual-Lumi™ II Luciferase Assay Kit (Beyotime Institute of Biotechnology, Shanghai, China). Fluorescence intensity was analyzed by CLARIOstar microplate reader (BMG Labtech).

### Establishment of GBM mouse model and treatments

Eight-week-old female C57/BL6 mice (the Beijing Vital River Laboratory Animal Technology Co. Ltd., Beijing, China) were used to establish the GBM mouse model. In brief, under anesthesia with 3% isoflurane, a 0.5 cm incision was made to expose the skull. Using a stereotaxic instrument with a 1.0 mm diameter cranial drill, a 1.0 mm diameter hole was drilled into the dura 1.0 mm anterior fontanelle and 2.5 mm to the right side of the midline. Luci-GL261 cells were injected at 1.5 × 10^5^ cells into the right caudate nucleus of the mouse using a precision syringe (1013086, Hamilton, New York,USA). The bone hole was closed with bone wax, and the scalp was closed using a 4.0 Vicryl suture. The mice were randomly assigned into four treatment groups: (1) Saline control; (2) TMZ treatment; (3) TMZ + PF-543; and (4) TMZ + PF-543 + Vemurafenib. TMZ at 50 ml/kg and Vemurafenib (MedChemExpress, #HY-12057) at 10 mg/kg were administered by gavage. PF-543 (MedChemExpress, #HY-15425) hydrochloride at 0.4 mg/kg was administered intraperitoneally. An equal volume of normal saline was used as a control. Seven days post-op, the mice were treated with their respective reagents. The tumor growth was monitored on day 7, day 14, and day 21 by bioluminescence imaging (IVIS system, UAS). For bioluminescence imaging, the mice were treated with 100 μl 15 mg/ml d-luciferin (PerkinElmer, Waltham, MA, USA) i.p. in PBS to obtain luciferase images. The ratio of relative flux value (versus day 7) in each mouse was used as the tumor growth rates. This study was conducted according to the experimental practices and principles approved by the Animal Welfare and Research Ethics Committee at Jilin University.

### Statistical analysis

All experiments were performed three times independently. All data are presented as means ± standard deviation (SD). Student’s *t*-test was used to conduct two experimental group comparisons, and analysis of variance (ANOVA) was used for multiple comparisons. All analytical graphs were plotted by GraphPad Prism 8 and RStudio (R version 4.2.3). *P* < 0.05 was considered statistically significant.

### Supplementary information

Supplemental file

Full and uncropped western blots

## Data Availability

All data are available from the corresponding author on reasonable request.
