# Supplementary material for: Triggering of endoplasmic reticulum stress via ATF4-SPHK1 signaling promotes glioblastoma invasion and chemoresistance
Source: Cell Death Dis. 2024 Aug 1;15(8):552. doi: 10.1038/s41419-024-06936-8 (PMC11294582; doi:10.1038/s41419-024-06936-8)
Supplement: Supplementary file 1 — Supplemental file [file 41419_2024_6936_MOESM1_ESM.docx]

**Triggering of Endoplasmic Reticulum Stress via ATF4-SPHK1 Signaling Promotes Glioblastoma Invasion and Chemoresistance**

**Supplementary materials**

Table 1. List of siRNA sequences.

| **siRNA** | **sequences** |
| --- | --- |
| siATF4-1 | AUCAUGGCAACGUAAGCAGTT |
| siATF4-2 | UUCUAUCAAAUCUUUCAGGTT |
| siATF4-3 | UUUCAGGUACUGGAUCUCCTT |
| siSPHK1 | GGCTGAAATCTCCTTCACG |

Table 2. List of antibodies

| **Antibodies** | **Source** | **Identifer** |
| --- | --- | --- |
| β-actin | ProteinTech Group | 23660-1-AP |
| BIP | ProteinTech Group | 11587-1-AP |
| Caspase-3 | ProteinTech Group | 66470-2-Ig |
| Bcl-2 | ProteinTech Group | 12789-1-AP |
| Bax | ProteinTech Group | 50599-2-Ig |
| Parp1 | ProteinTech Group | 13371-1-AP |
| SPHK1 | ProteinTech Group | 10670-1-AP |
| p-SPHK1 | ProteinTech Group | 19561-1-AP |
| eIF2α | Cell Signaling Technology | 9722 |
| ATF4 | Cell Signaling Technology | 11815 |
| N-cadherin | Cell Signaling Technology | 13116 |
| E-cadherin | Cell Signaling Technology | 96743 |
| Vimentin | Cell Signaling Technology | 5741 |
| Anti-mouse secondary antibody | Cell Signaling Technology | 7076 |
| Anti-mouse secondary antibody | Cell Signaling Technology | 7076 |
| p-eIF2α | Affinity Biosciences | AF3087 |
| p-PERK | Affinity Biosciences | DF7576 |
| γ-H2AX | Abcam | ab81299 |
| Anti-Rabbit fluorescent secondary antibody | Thermo | A11034 |

Table 3. List of primer sequences

| **primer** | **sequences** |
| --- | --- |
| ATF4 | Forward- ATGACCGAAATGAGCTTCCTG |
|  | Reverse- GCTGGAGAACCCATGAGGT |
| SPHK1 | Forward- GCTCTGGTGGTCATGTCTGG |
|  | Reverse- CACAGCAATAGCGTGCAGT |
| Snail2 | Forward- AACAGTATGTGCCTTGGGGG |
|  | Reverse- AAAAGGCACTTGGAAGGGGT |
| E-cadherin | Forward- GCTGGACCGAGAGAGTTTCC |
|  | Reverse- CAAAATCCAAGCCCGTGGTG |
| N-cadherin | Forward- CCCTGCTTTCATTCTGACATACC |
|  | Reverse- CTGCCACTTGCCACTTTTCC |
| Vimentin | Forward- AGAGGAAGCCGAAAACACCC |
|  | Reverse- TCAAGGTCAAGACGTGCCAG |

Table 4. List of CHIP-qPCR primer sequences

| **primer** | **sequences** |
| --- | --- |
| Pro-SPHK1 | Forward- CCAGGTGGGCTTTATCACCA |
|  | Reverse- CCGAGATCTCAGCCTTCGAG |
| Pro-HMOX1 | Forward- CACCACCTTCATCCCAGTCC |
|  | Reverse- AGCATGTCTCATGTTGGAAGT |
| GAPDH | Forward- TACTAGCGGTTTTACGGGCG |
|  | Reverse- TCGAACAGGAGGAGCAGAGAGCGA |

Table 5. CHIP-qPCR calculation method

| **term** | **calculation method** |
| --- | --- |
| delt-CT | IP-CT – (Input-CT – log_2_100) |
| enrichment (%Input) | 2^^delt-CT^ |

Table 6. ER stress associated gene expression

| **Gene name** | **Fold Change** | **pvalue** | **Function** |
| --- | --- | --- | --- |
| DNAJB9 (DnaJ heat shock protein family (Hsp40) member B9) | 11.32 | 6.92E-137 | Facilitate protein folding |
| HERPUD1 (Homocysteine inducible ER protein with ubiquitin like domain 1) | 3.03 | 2.09E-116 | Participate in the ERAD process |
| SELENOK (Selenoprotein K) | 3.1 | 1.13E-83 | Participate in the ERAD process and maintain ER Ca2+ homeostasis |
| SERP1 (Stress associated endoplasmic reticulum protein 1) | 2.47 | 2E-83 | Facilitate protein folding |
| MANF (mesencephalic astrocyte derived neurotrophic factor) | 2.43 | 1.55E-74 | Maintain ER Ca2+ homeostasis |

**Supplementary methods**

Transfection of siRNA and plasmid

Small interfering RNA (si)-ATF4, siSPHK1 and pcDNA3.1-ATF4 plasmid were purchased from GenePharma Bio Co. Ltd. (Shanghai, China). The siRNA sequences are listed in Table 1. Cells were seeded in 6-well plates at a density of 1.5 × 10^5^ cells per well. After subculture for 24 hours, siRNA (sequences see Table 1) at 10 nM or pcDNA3.1-ATF4 overexpression plasmid at 2.5 μg was transfected to the respective GBM cells using 6 μl TurboFect Transfetion Reagent (Thermo Fisher Scientific, MA, USA, #R0531).

Western blotting analysis

Cells were lysed using RIPA buffer supplement with proteasome inhibitors. After sonicated and centrifuged, lysate protein concentrations were measured using a Bradford protein assay kit (Beyotime Institute of Biotechnology, Shanghai, China, #P0006). Protein samples were separated by 12% SDS-PAGE gels and transferred to PVDF membranes. After blocked with 5% skim milk for 1.5 hours, the membranes were immunoblotted with the primary antibodies overnight at 4℃. Next day, the membranes incubated with the secondary antibodies for 1.5 hours at room temperature. The membranes were visualized using Syngene Bio Imaging (Synoptics, Cambridge, UK) and detected by the electrochemiluminescence detection method. ImageJ software was used to semi-quantify protein expression. The antibodies are listed in Table 2.

Immunofluorescence

Cells were seeded on coverslips in 24-well plates at a density of 2 × 10^4^ cells per well. After treatments, cells were fixed with 4% paraformaldehyde for 20 minutes and permeabilized with 0.1% (v/v) Triton-PBS for 8 minutes at room temperature. Then, cells were blocked with 0.2 % BSA-PBS for 1 hour and incubated with the primary antibody at 4℃ overnight. Next day, cells were incubated with fluorescent secondary antibody (1:500) for 1 hour and Hoechst-33342 (Invitrogen, #H1399) for 8 minutes at room temperature. After washing three times with PBS, immunofluorescence images were captured by Hybrid Microscope (Discover ECHO).

RT-qPCR assay

Total RNA was extracted using Trizol reagent and cDNA was synthesized using a Hifair® II 1st Strand cDNA Synthesis Kit (Yeasen Biotechnology Co., Ltd, Shanghai, China, #11123ES60). RT-qPCR was performed using Hieff® qPCR SYBR Green Master Mix (Yeasen, #11202ES08) by three-steps method. Data was analyzed using delt-delt-CT method and quantified with β-actin (Sangon Biotech, Shanghai, China). The primer sequences are presented in Table S2.

Flow cytometry

For apoptosis assay, cells were seeded in 6-well plates at a density of 1.5 × 10^5^ cells per well. After treatment, apoptosis assay was performed using Apoptosis Detection Kit (Sungene Biotech Co., Ltd, Tianjin, China, #AO2001-11P-H) according to the manufacturer's protocol. After digestion and centrifugation, 1 × 10^5^ cells were resuspended in 1.5 mL centrifuge tubes using 100 μL 1 × Binding Buffer. Added 5 μL of Annexin V-APC to the tubes and incubated for 10 mins at room temperature protected from light. Then added 5 μL PI solution to the tubes and incubated for 5 mins in room temperature protected from light. Finally, added PBS to 500 μL and analized using Guava® easyCyte flow cytometer (Merck KGaA).

For mitochondrial membrane potential assay, cells were seeded in 6-well plates at a density of 1.5 × 10^5^ cells per well. After treatment, mitochondrial membrane potential assay was performed using JC-1 kit (Beyotime Biotechnology) according to the manufacturer's protocol. After digestion and centrifugation, 1 × 10^5^ cells were resuspended in 1.5 mL centrifuge tubes using 500 μL culture medium. Added 500 μL 1 × JC-1 staining solution to the tubes and incubated at 37ºC for 20min. Then cells were washed for three times and resuspended using 200 μL JC-1 buffer. Finally, cells were analyzed using Guava® easyCyte flow cytometer (Merck KGaA).

Wound healing assay

Cells were seeded in 24‑well plates at 1 × 10^5^ cells per well. The wound was produced using a 200 µl pipette tip. After washing with PBS three times, FBS‑free medium was added to the wells. The wound was photographed using a phase‑contrast microscope every 24 hours. ImageJ software was used to calculate the wound areas.

Trans-well invasion assay

After treatments, cells were isolated, centrifuged and resuspended in serum-free medium. The cells concentration was adjusted to 5 × 10^5^ cells/mL. 100 μL of cell suspension was added to the upper chamber which were coated with Matrigel (BD, USA, #356234) and 600 μL of culture medium containing 10% FBS was added to the lower chamber. After 12 hours, cells in the lower chamber were wash with PBS and fixed with 4% paraformaldehyde for 20 min, then stained with 0.1% crystal violet for 15 min at room temperature. After washed with ddH_2_O, images of the cells were captured using Hybrid Microscope (Discover ECHO) and analyzed by ImageJ.

Immunohistochemical staining

Tumor tissues were harvested and fixed in formalin for paraffin embedding. The tissues sections (5 μm) were then subjected for immunohistochemical staining (anti-ATF4 dilution ratio 1:500, anti-SPHK1 dilution ratio 1:400). IHC images were captured by Hybrid Microscope (Discover ECHO) and IHC results were evaluated using a semiquantitative approach. Based on the staining intensity, the overall score of is four categories: negative (0), weak (1), moderate (2), and strong (3). Based on the percentage of positive cells, the overall scoring is, 0: ≤ 25%, 1: 26%-50%, 2: 51%-75%, and 3: >75%. The two categories are multiplied to get the final score.

**Supplementary Figures**


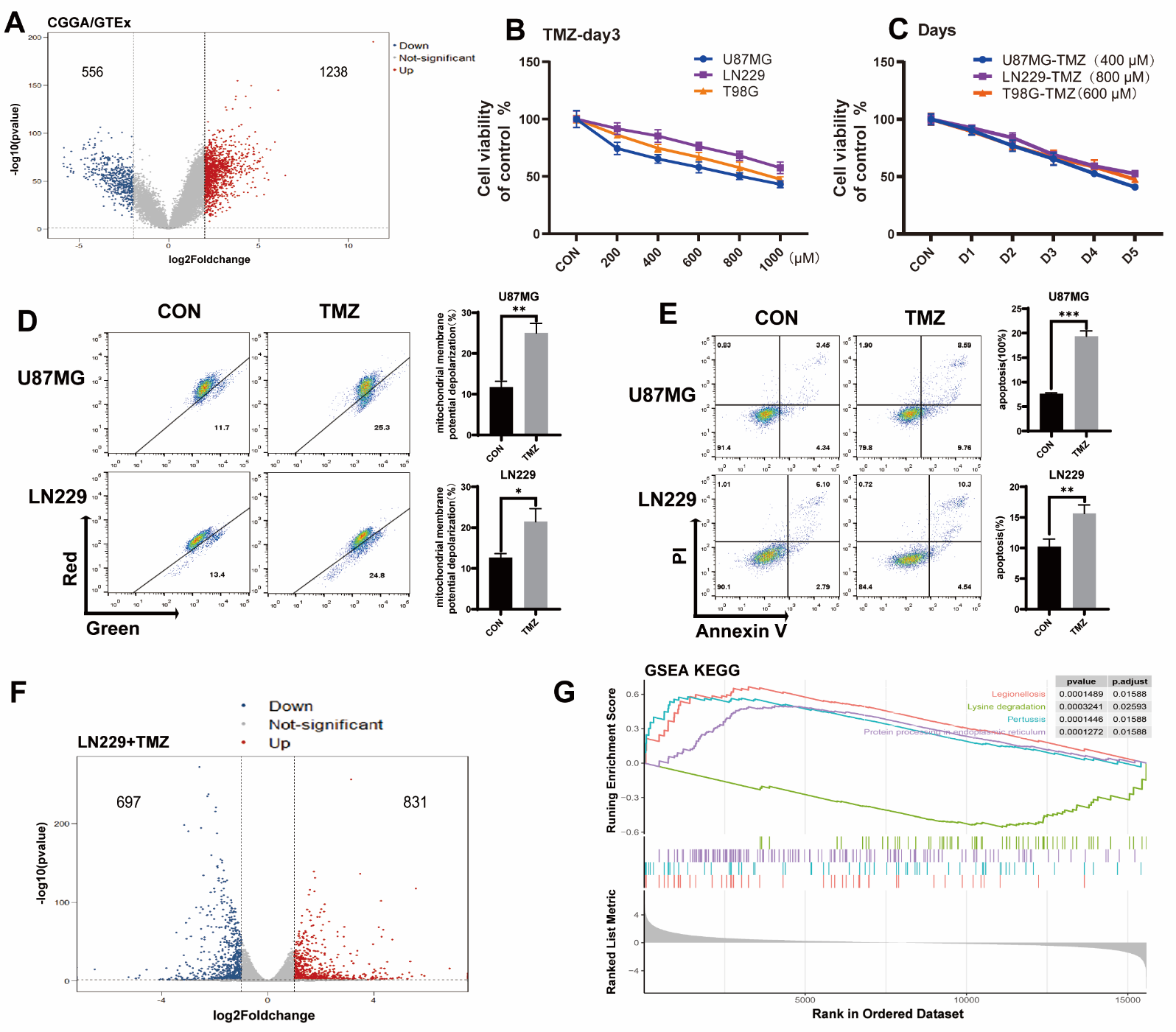


**Figure S1.** **A**: Volcano plot of the differential gene expression between GBM tissues (CGGA database) and normal tissues (GTEx Biobank). **B-C**: Cell viability detected by MTT assay in U87MG, LN229, and T98G cells treated with TMZ at different concentrations or at different time points. **D-E:** Detection of the changes in mitochondrial membrane potential and apoptosis by Flow cytometry in U87MG and LN229 cells treated with TMZ for 3 days. **F:** Volcano plot of the differential gene expression between untreated LN229 cells and the LN229 cells treated with TMZ for 3 days. **G:** GSEA analysis of the top 4 significantly altered signaling pathways (based on p.adjust) in LN229 cells treated with TMZ for 3 days. (*: *P* < 0.05, **: *P* < 0.01, ***: *P* < 0.001)


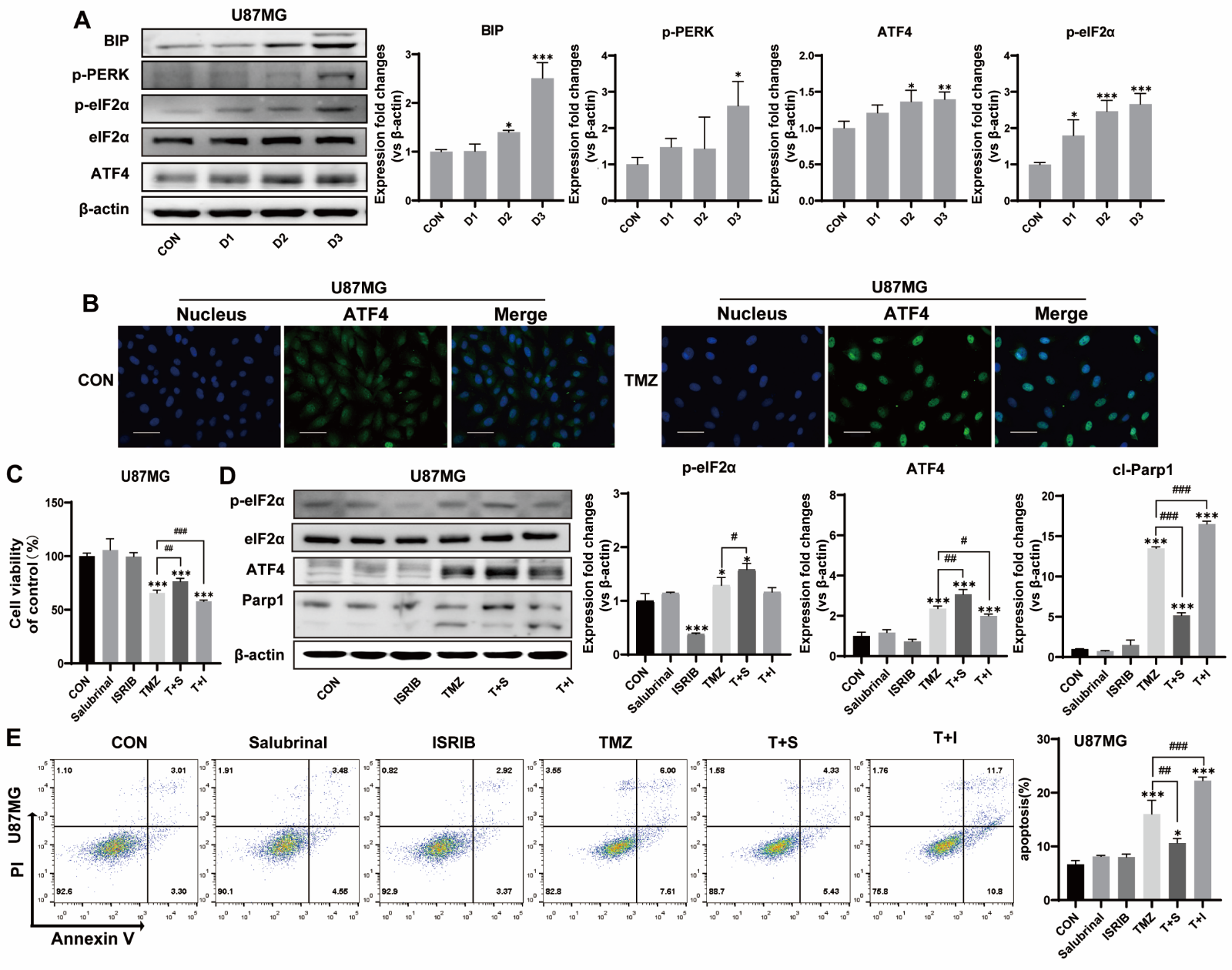


**Figure S2. A:** The protein levels of the major components in PERK pathway detected by Western blotting analysis in U87MG cells with TMZ treatment for 3 days. **B:** ATF4 expression and subcellular localization detected by immunofluorescence assay in U87MG cells with TMZ treatment for 3 days. **C:** MTT assay detected the cell viability in U87MG treated with TMZ for 3 days. Salubrinal (5 μM) and ISRIB (400 nM) were used. **D:** The protein levels of the major components in PERK pathway and apoptotic protein Parp1 detected by Western blotting analysis in U87MG cells. Salubrinal (5 μM) and ISRIB (400 nM) were used. **D:** Flow cytometry detected apoptosis in U87MG cells treated with TMZ for 3 days. Salubrinal (5 μM) and ISRIB (400 nM) were used. (*: *P* < 0.05, **: *P* < 0.01, ***: *P* < 0.001; #: *P* < 0.05, ##: *P* < 0.01, ###: *P* < 0.001)

**
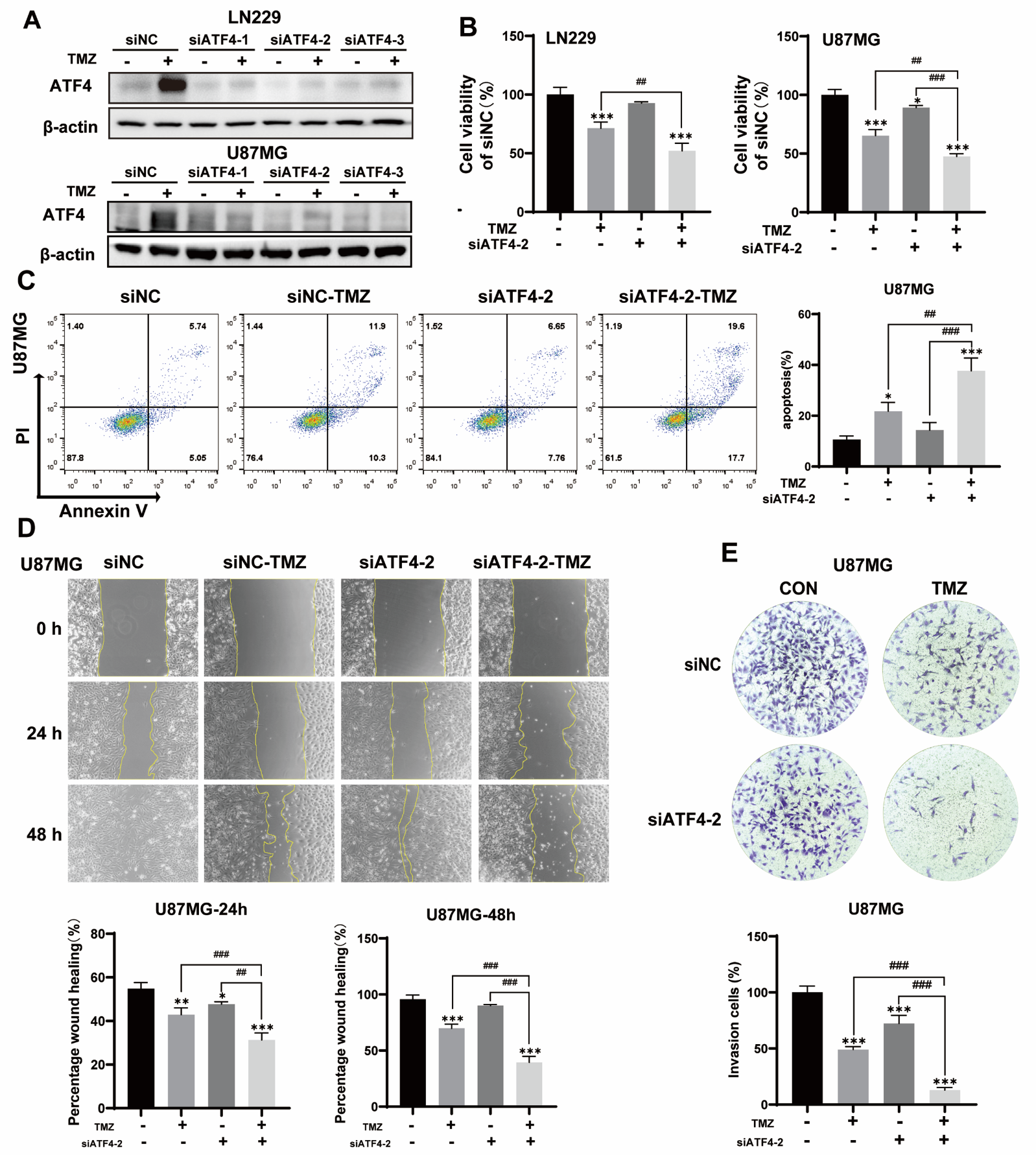
**

**Figure S3. A:** Western blotting analysis detected the ATF4 protein expression in U87MG and LN229 cells using siATF4-1, siATF4-2, and siATF4-3 to silence ATF4 expression. **B:** MTT assay detected cell viability in U87MG-siATF4-2 cells and LN229-siATF4-2 cells with TMZ treatment for 3 days. **C:** Flow cytometry detected cell apoptosis in U87MG-siATF4-2 cells with TMZ treatment for 3 days. **D:** Wound healing assay detected the migration of U87MG-siATF4-2 cells with TMZ treatment for 24 hours and 48 hours under serum-free culture conditions. **E:** Trans-well invasion assay detected the cell invasion of U87MG-siATF4-2 cells with TMZ treatment for 12 hours. (*: *P* < 0.05, **: *P* < 0.01, ***: *P* < 0.001; ##: *P* < 0.01, ###: *P* < 0.001)


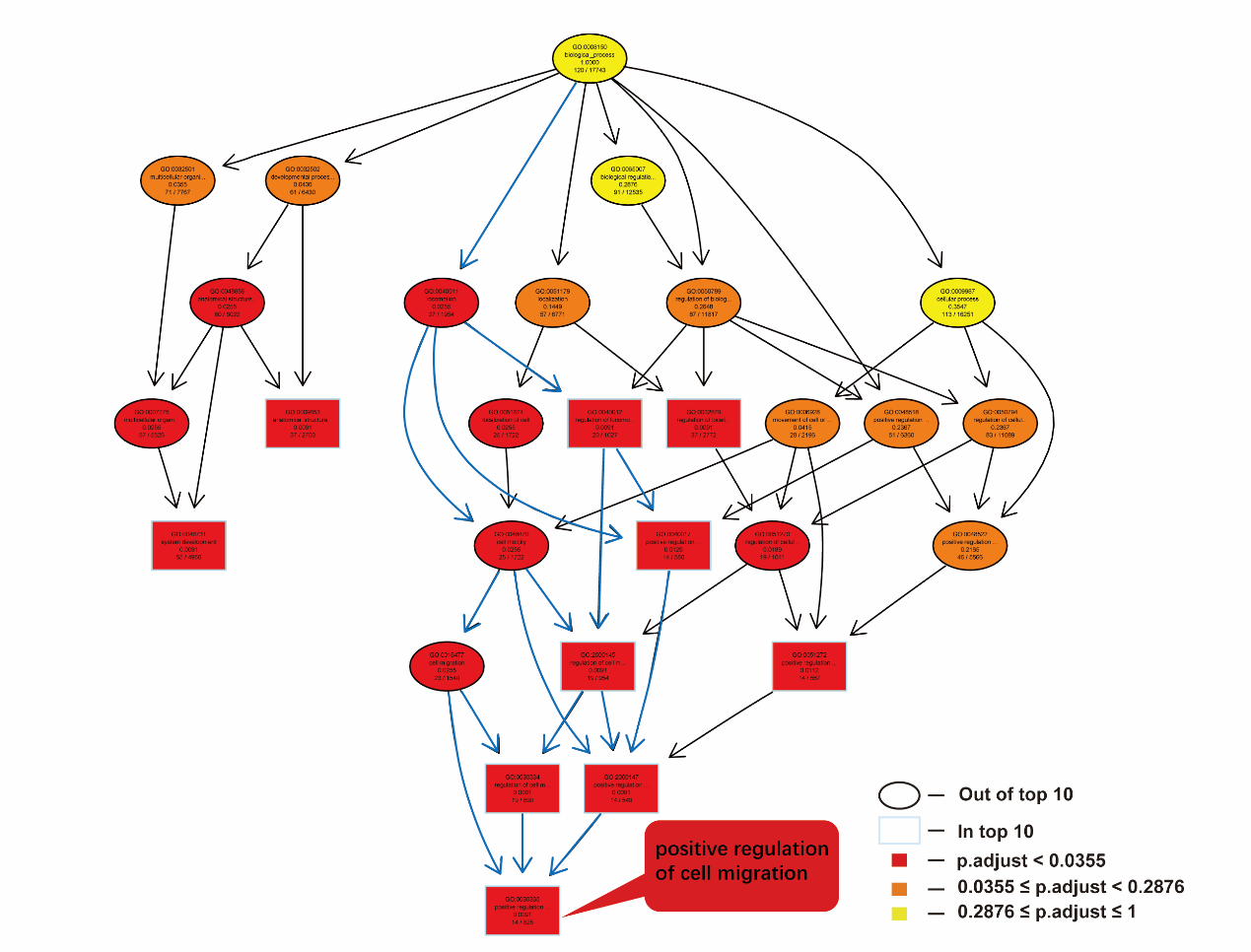


**Figure S4.** The GO enrichment analysis directed acyclic graph (DAG) of the ATF4 binding genes to the promoter regions.


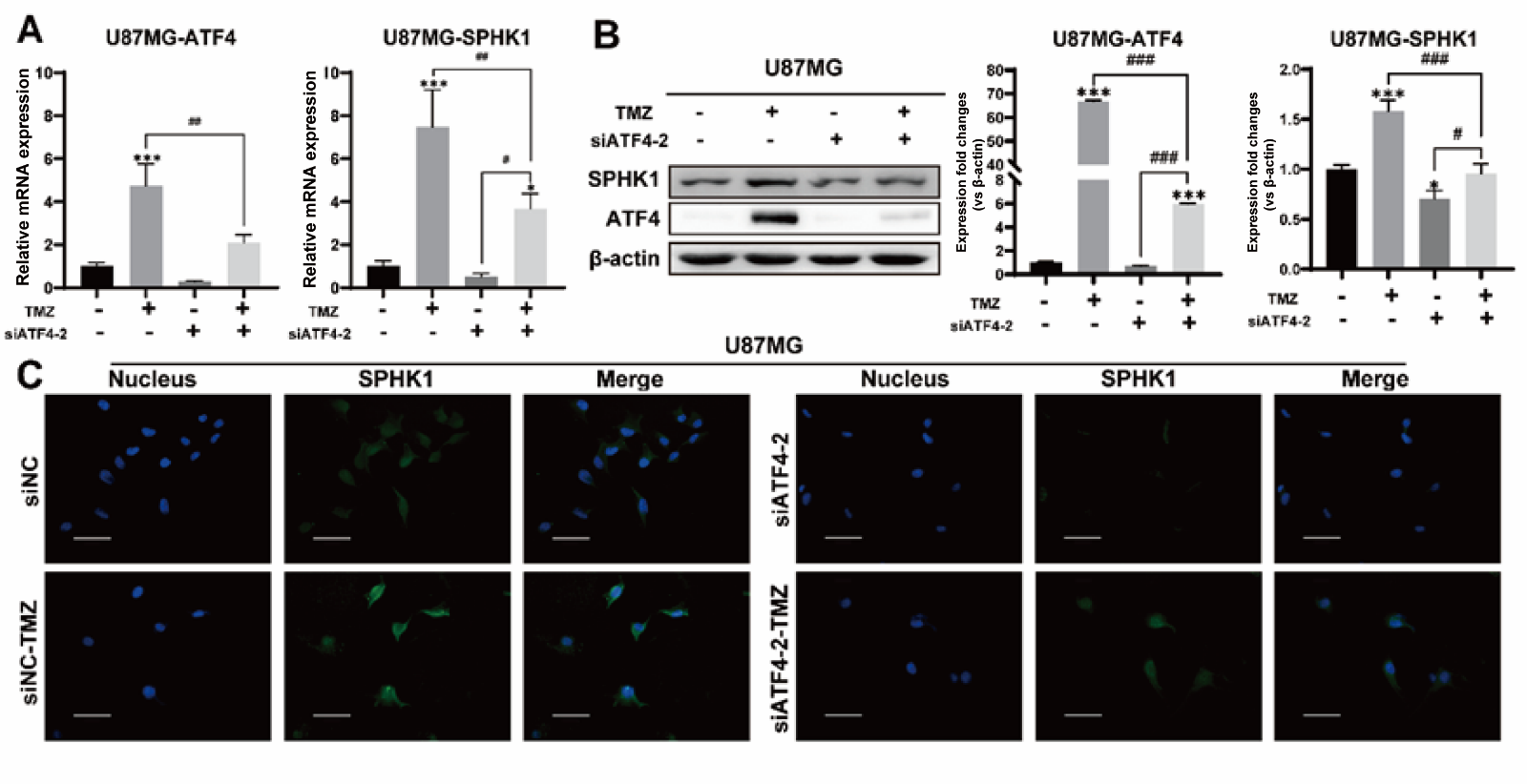


**Figure S5.** **A:** The mRNA levels of SPHK1 and ATF4 by qPCR in U87MG-siATF4-2 cells and U87MG cells with TMZ treatment for 3 days. **B:** Protein levels of SPHK1 and ATF4 by Western blotting analysis in U87MG-siATF4-2 cells and U87MG cells with TMZ treatment for 3 days. **C:** Immunofluorescent staining detection of SPHK1 expression and subcellular localization. in U87MG-siATF4-2 cells and U87MG cells with TMZ treatment for 3 days. (*: *p* < 0.05, ***: *p* < 0.001; #: *p* < 0.05, ##: *p* < 0.01, ###: *p* < 0.001)


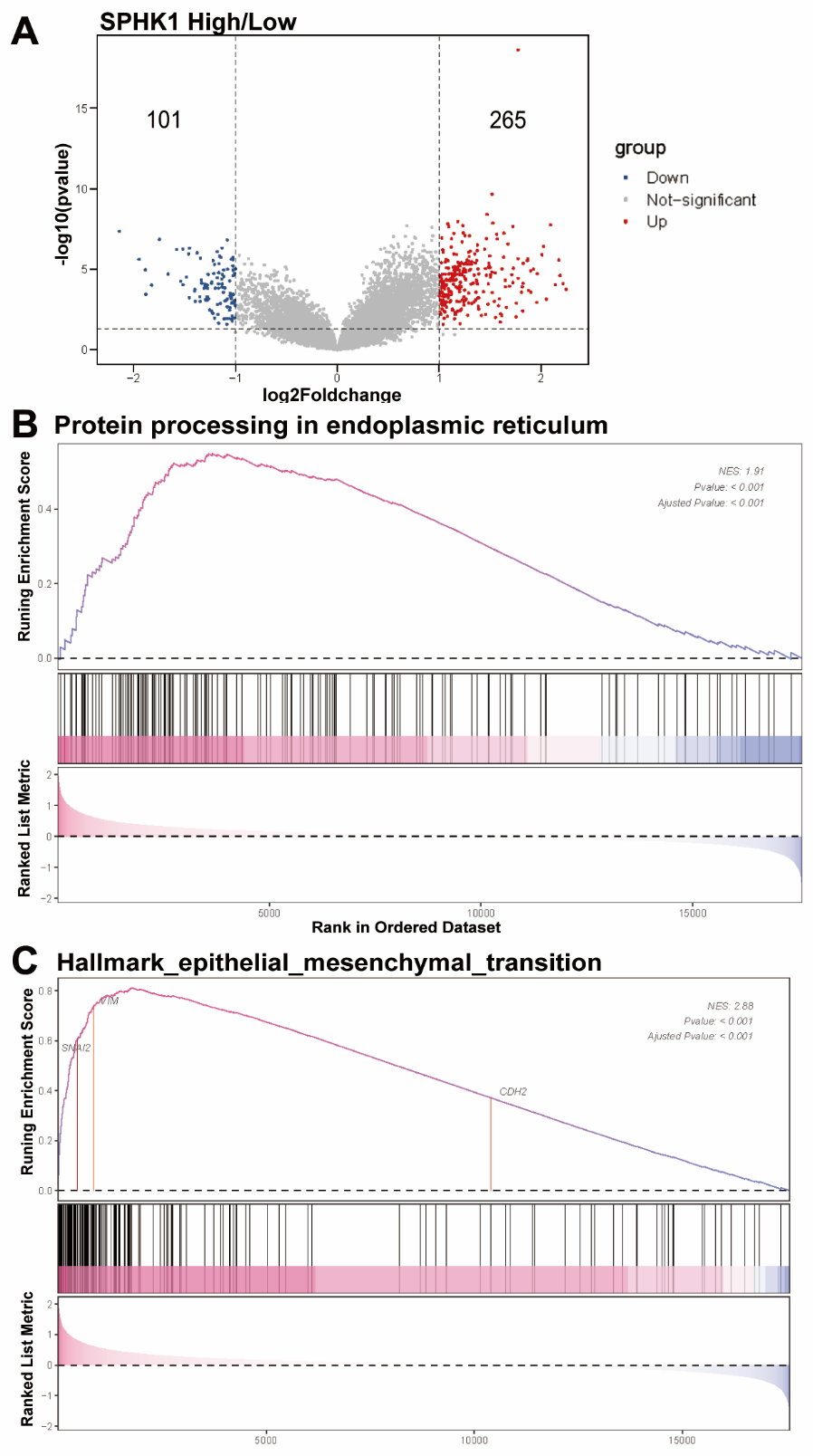


**Figure S6.** **A**: Volcano plot of the differential gene expression of RNA-seq data from web-based dataset (CGGA325) between SPHK1 expression levels (high, low) based on the median number of GBM samples. **B:** Single-gene GSEA of RNA-seq data from web-based dataset (CGGA325) linking to the pathway of protein processing in endoplasmic reticulum based on SPHK1 expression. **C:** Single-gene GSEA of RNA-seq data from web-based dataset (CGGA325) linking to the EMT pathway based on SPHK1 expression.


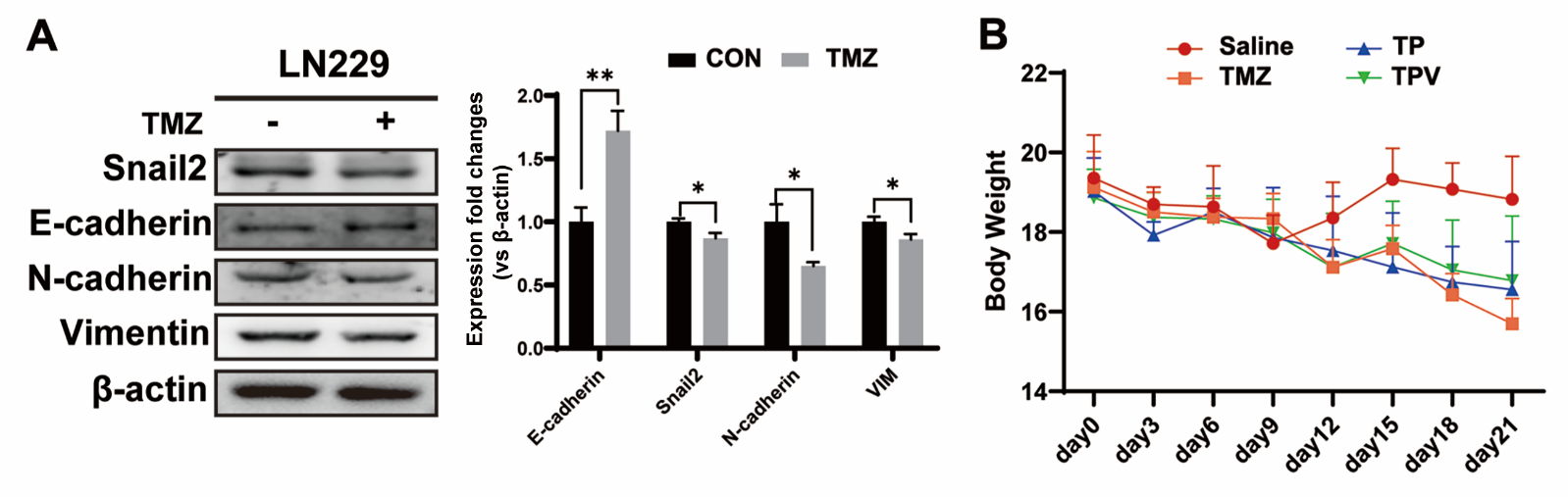


**Figure S7.** **A**: Protein levels of EMT associated proteins by Western blotting analysis in LN229 cells with TMZ treatment for 3 days. **B**: Effect of glioma development and TMZ, TMZ+PF-543 (TP), TMZ+PF-543+ Vemurafenib (TPV) administration on mice body weight. (*: *p* < 0.05, ***: *p* < 0.001)
