# Supplementary material for: Triggering of endoplasmic reticulum stress via ATF4-SPHK1 signaling promotes glioblastoma invasion and chemoresistance
Source: Cell Death Dis. 2024 Aug 1;15(8):552. doi: 10.1038/s41419-024-06936-8 (PMC11294582; doi:10.1038/s41419-024-06936-8)

**Fig1.D**

**BIP**

**
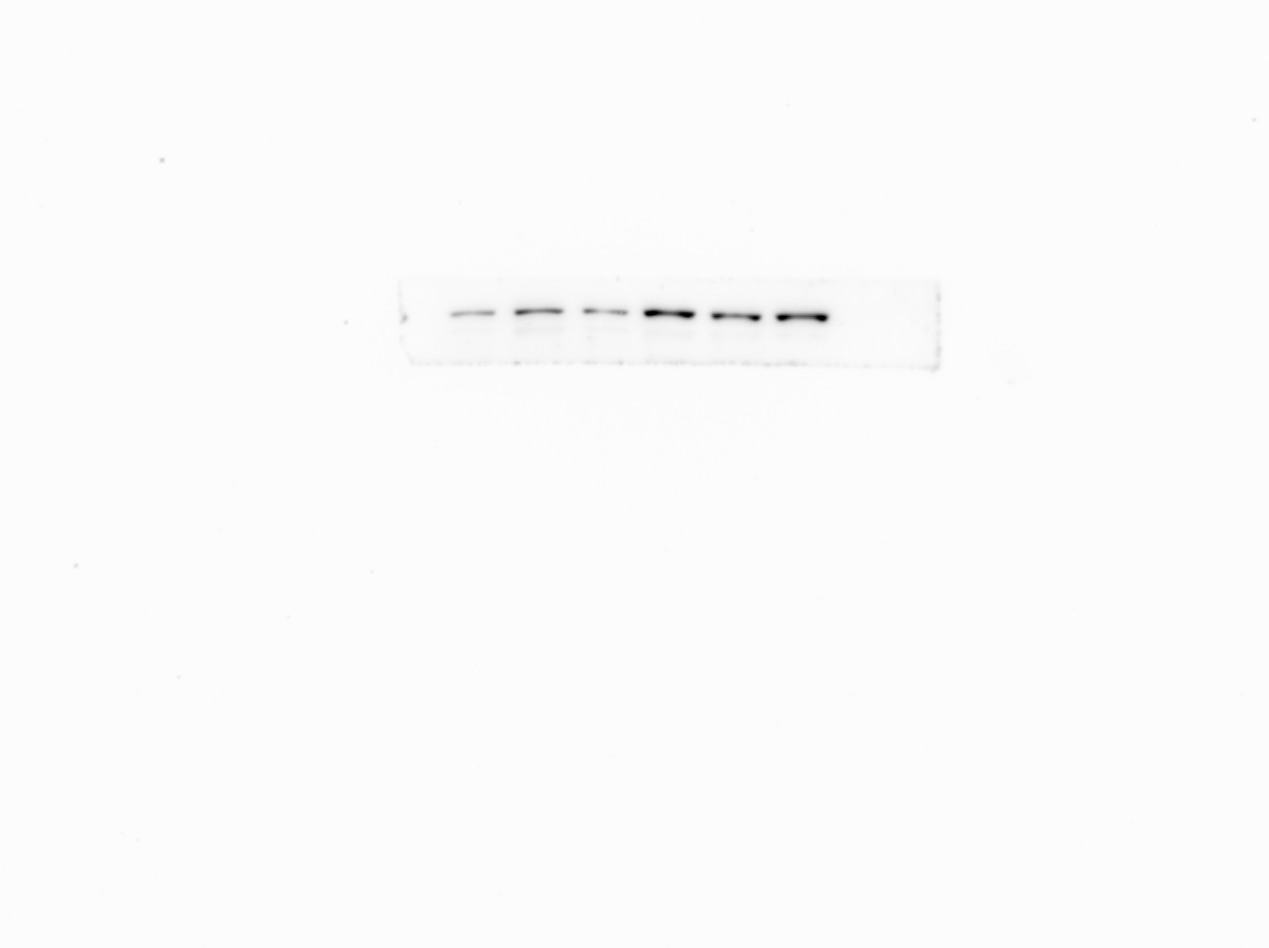
**

**P-PERK**

**
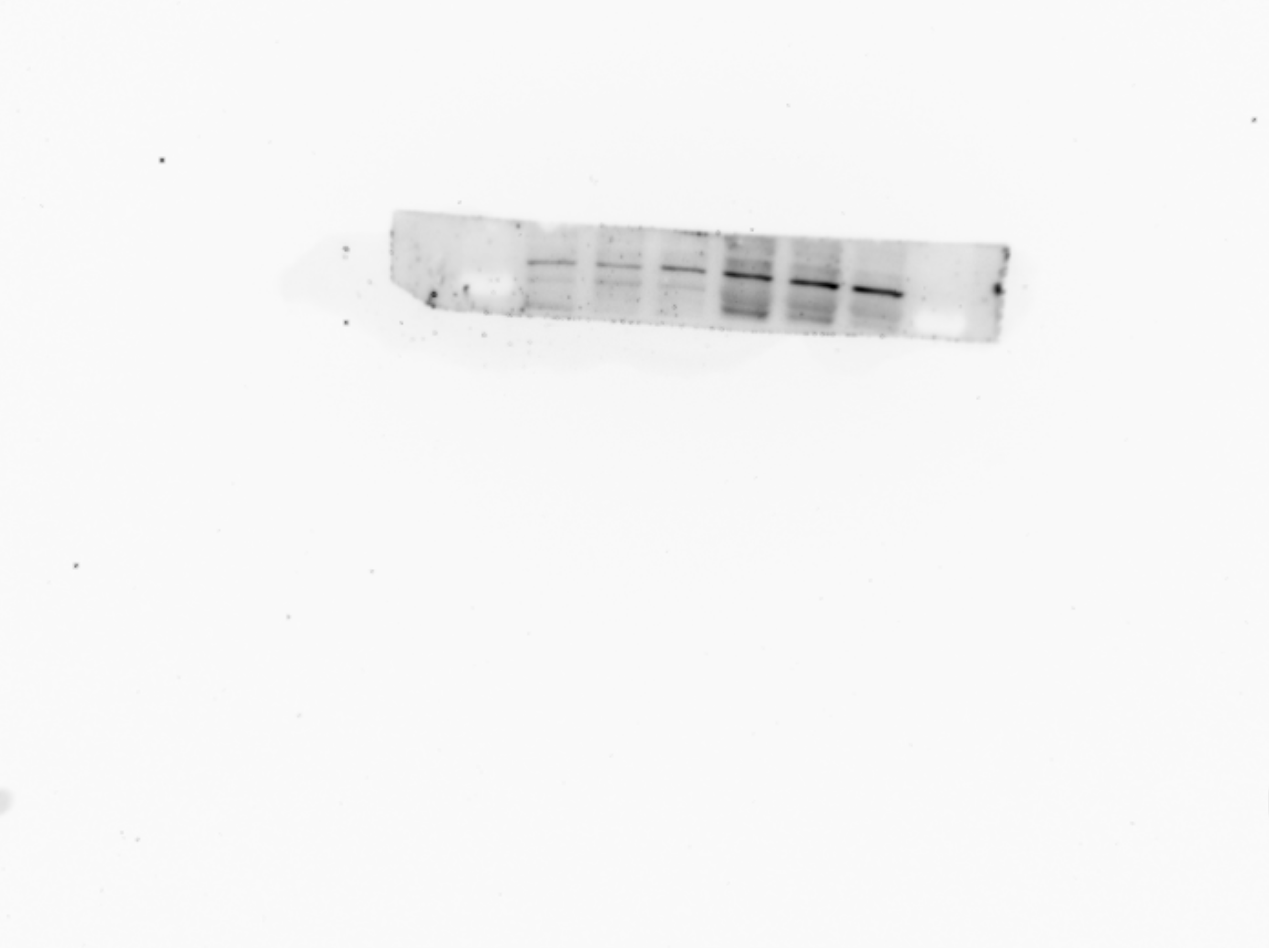
**

**p-eIF2α**

**
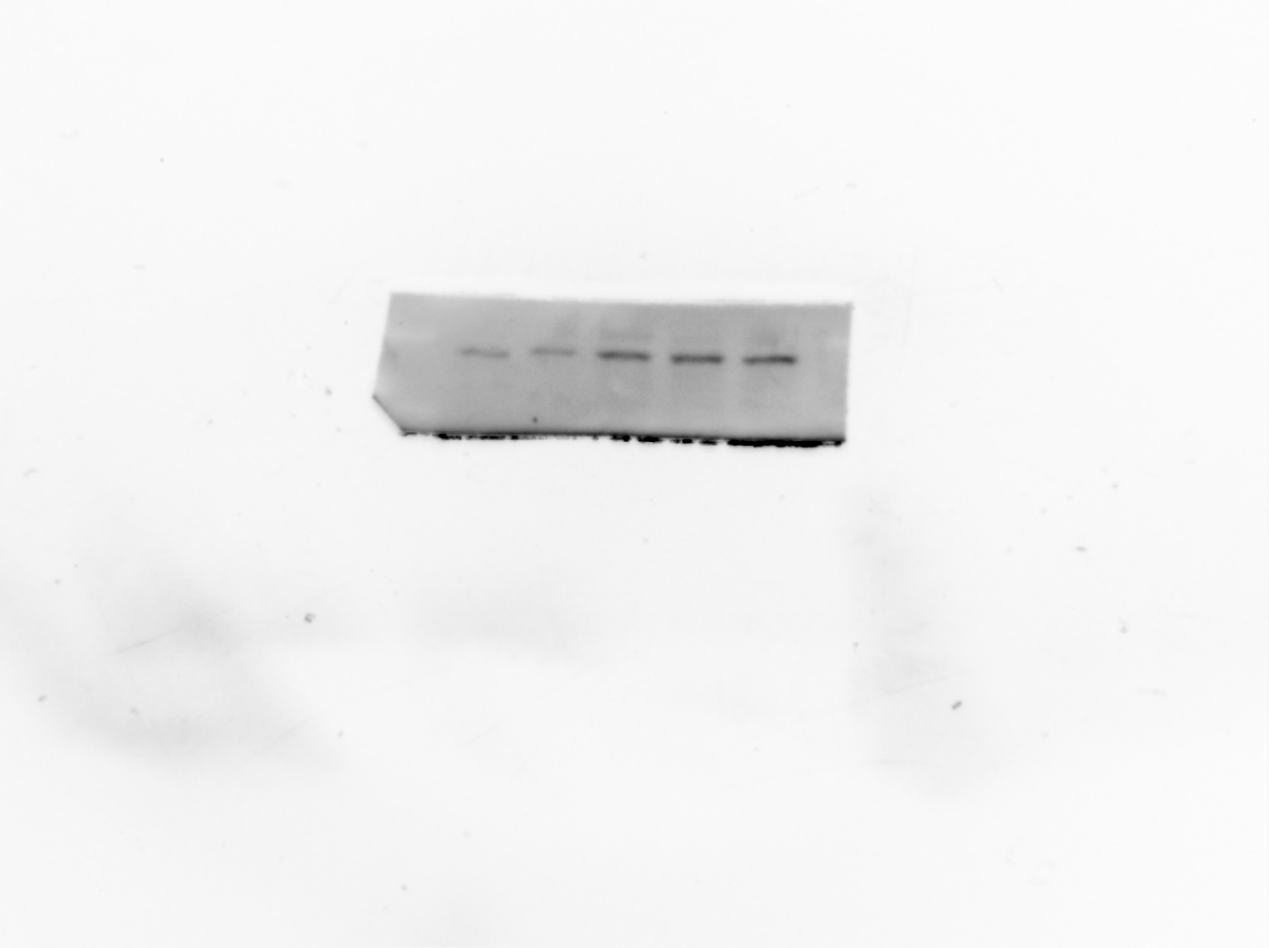
**

**eIF2α**

**
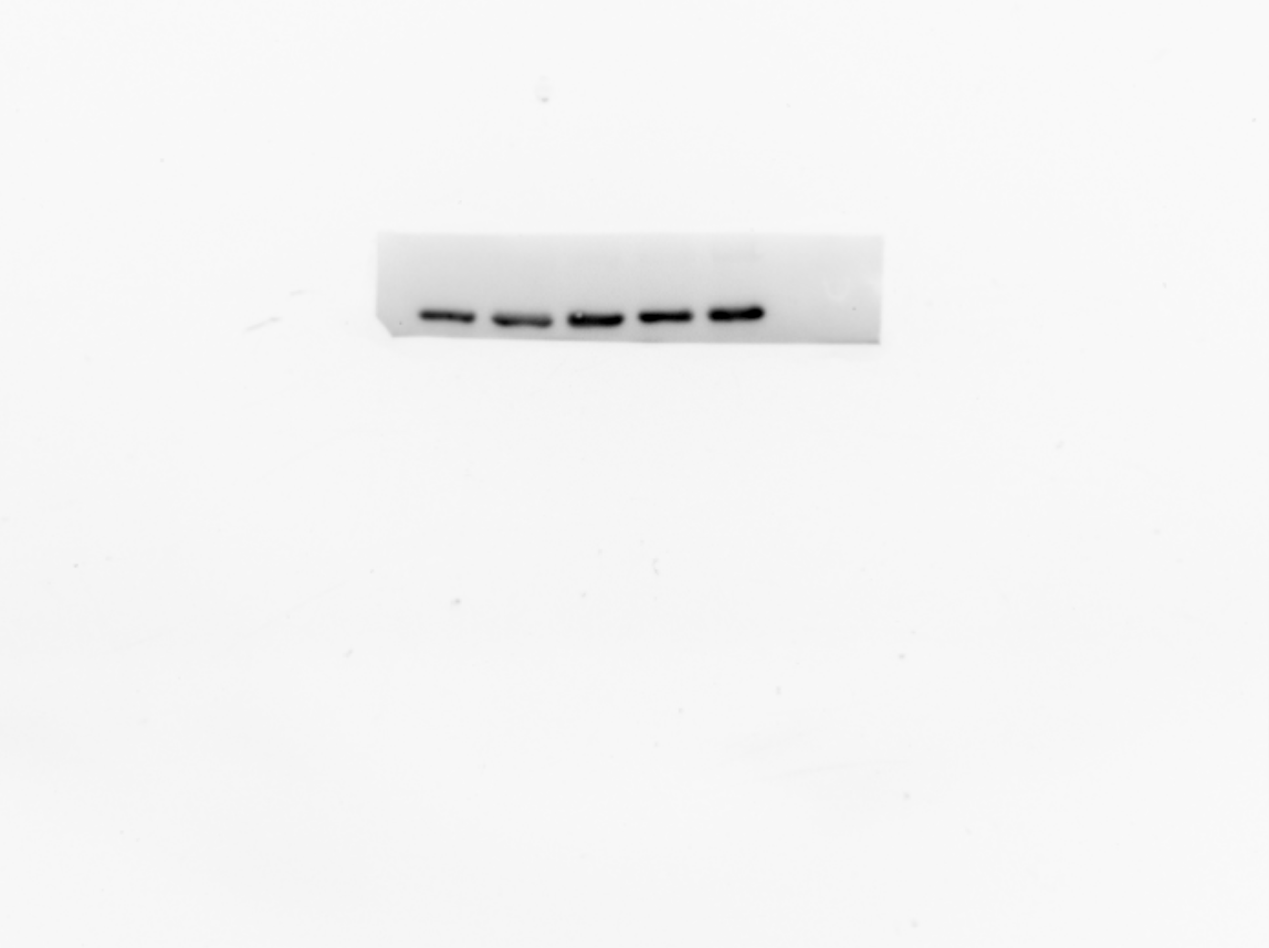
**

**β-Actin**

**
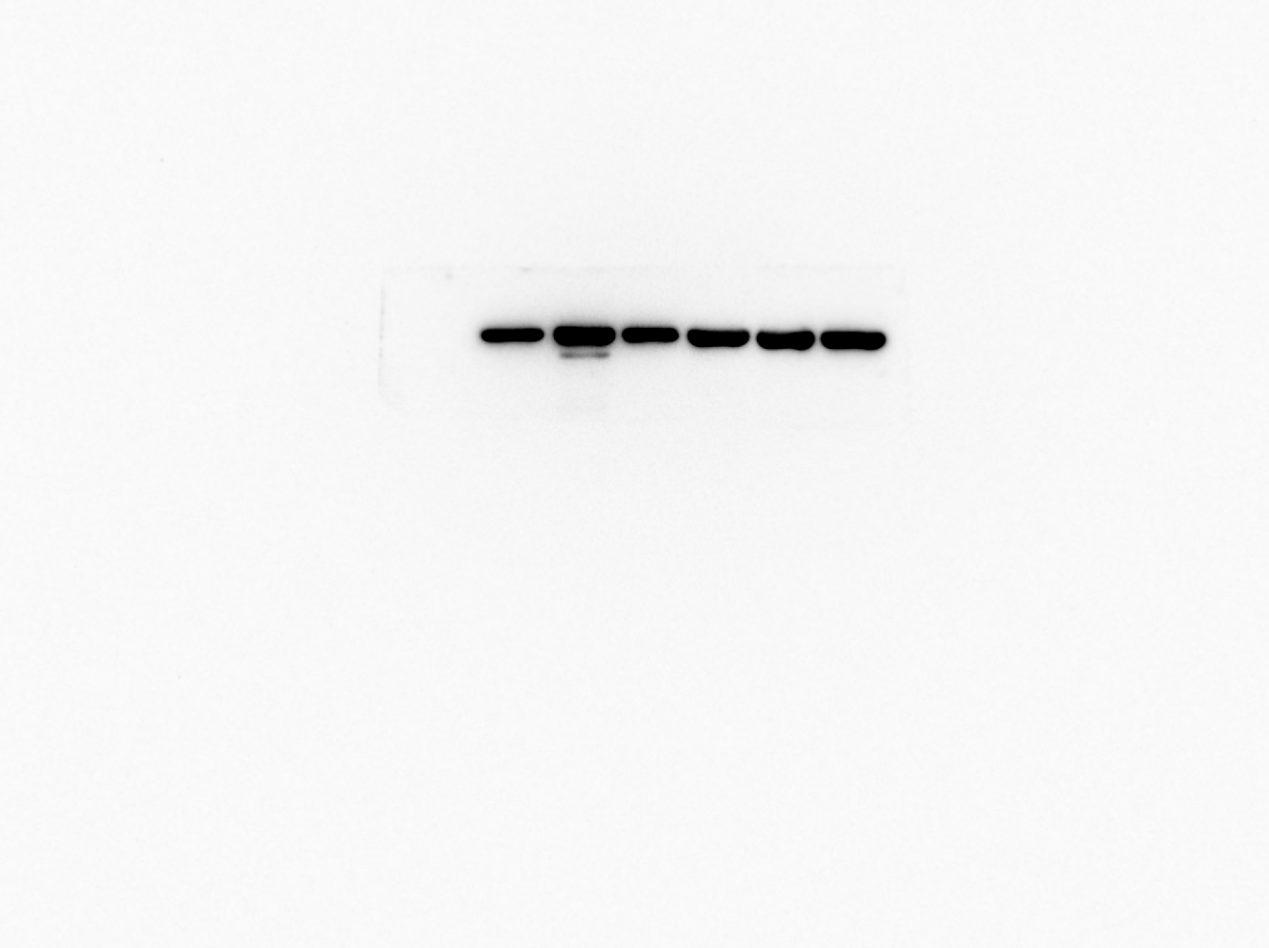
**

**Fig1.H**

**SPHK1**

**

**

**ATF4**

**
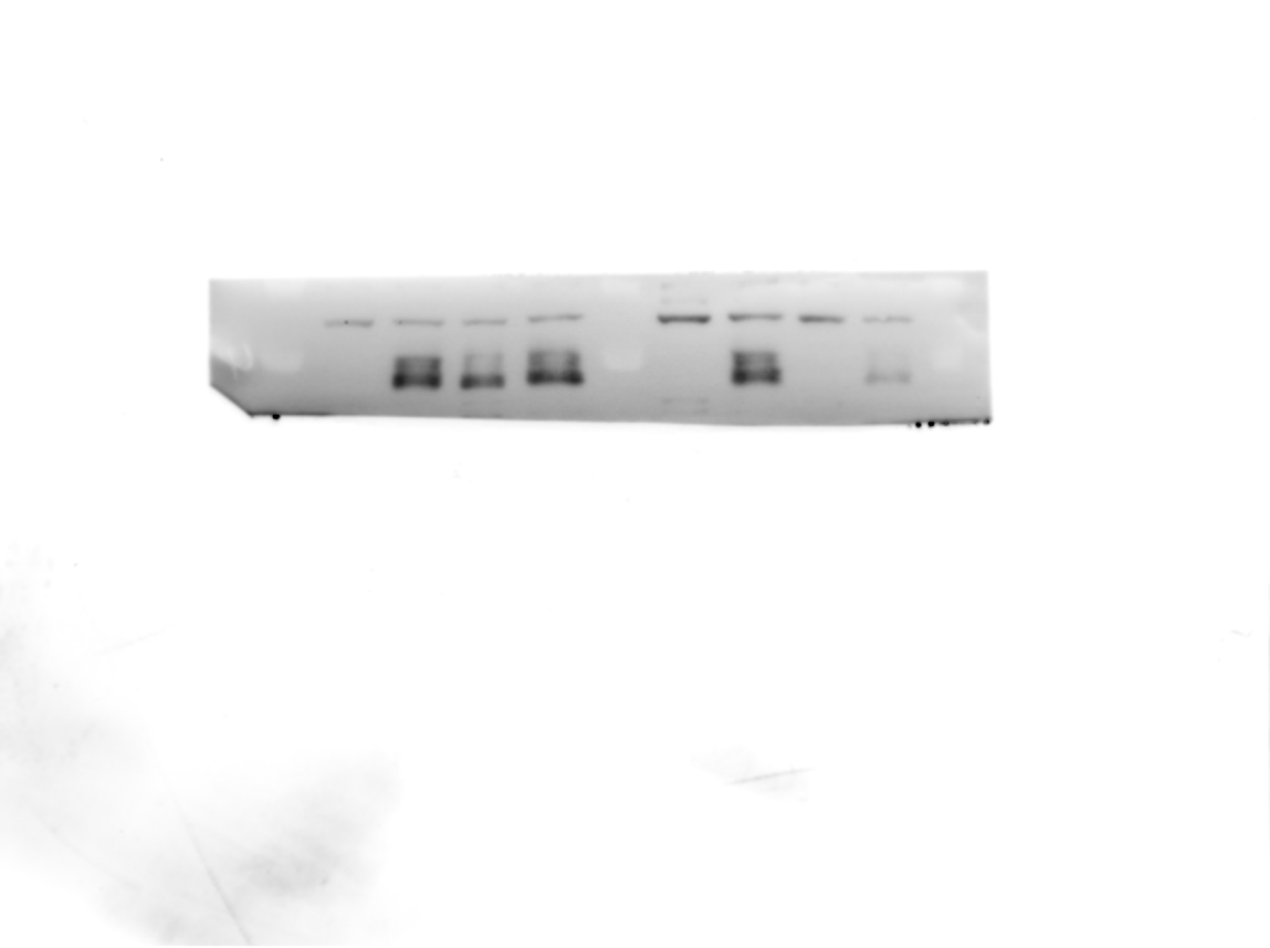
**

**Caspase-3**

**

**

**β-Actin**

**
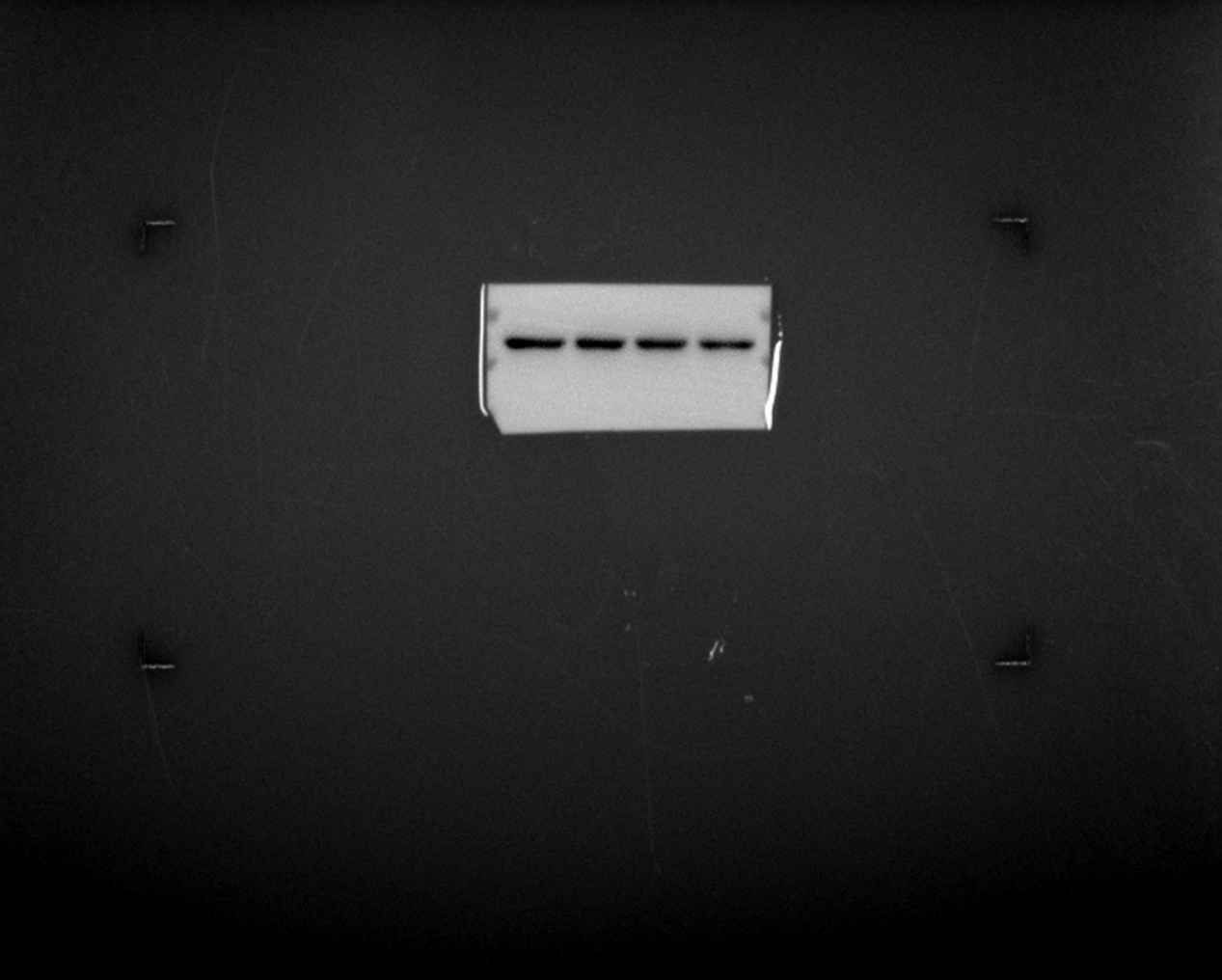
**

**Fig2.B**

**p-eIF2α**

**
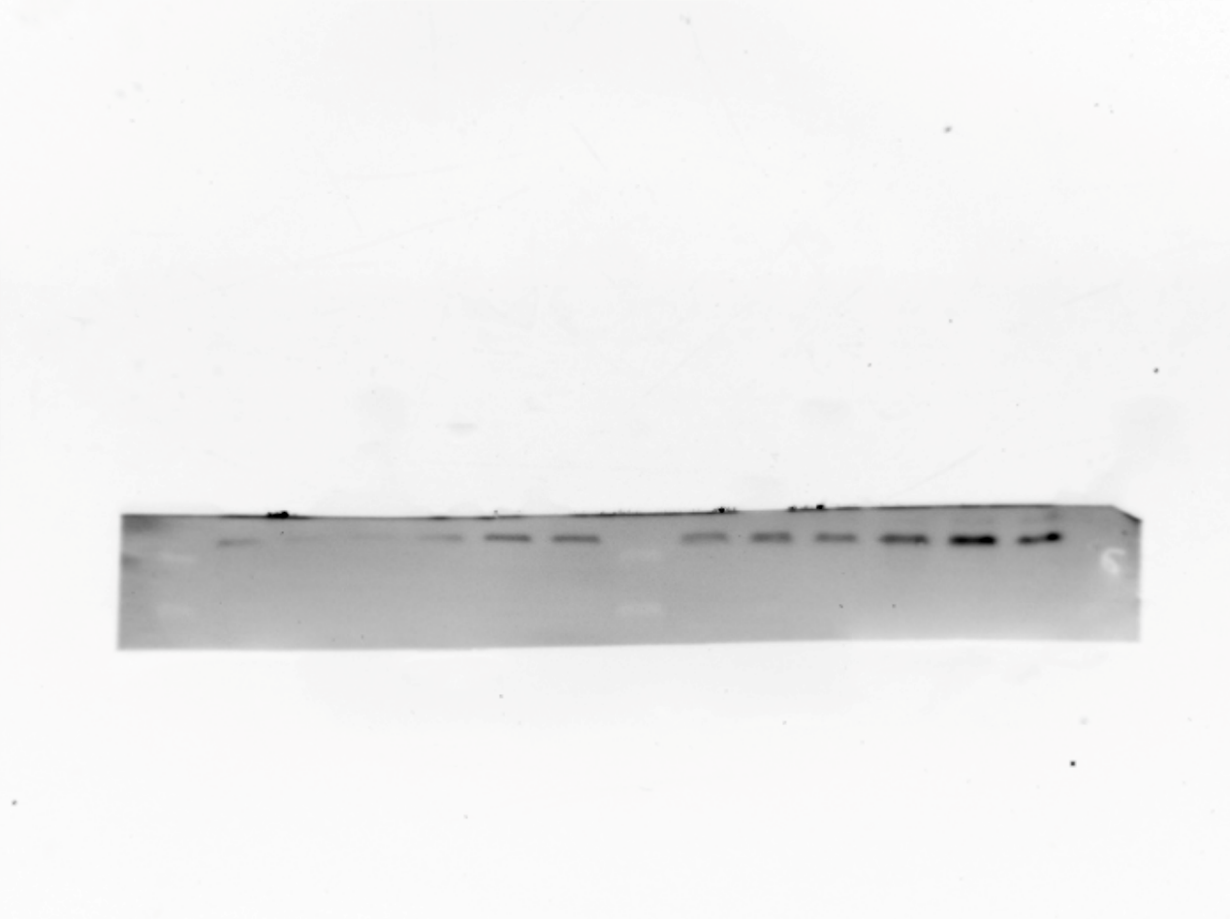
**

**eIF2α**

**
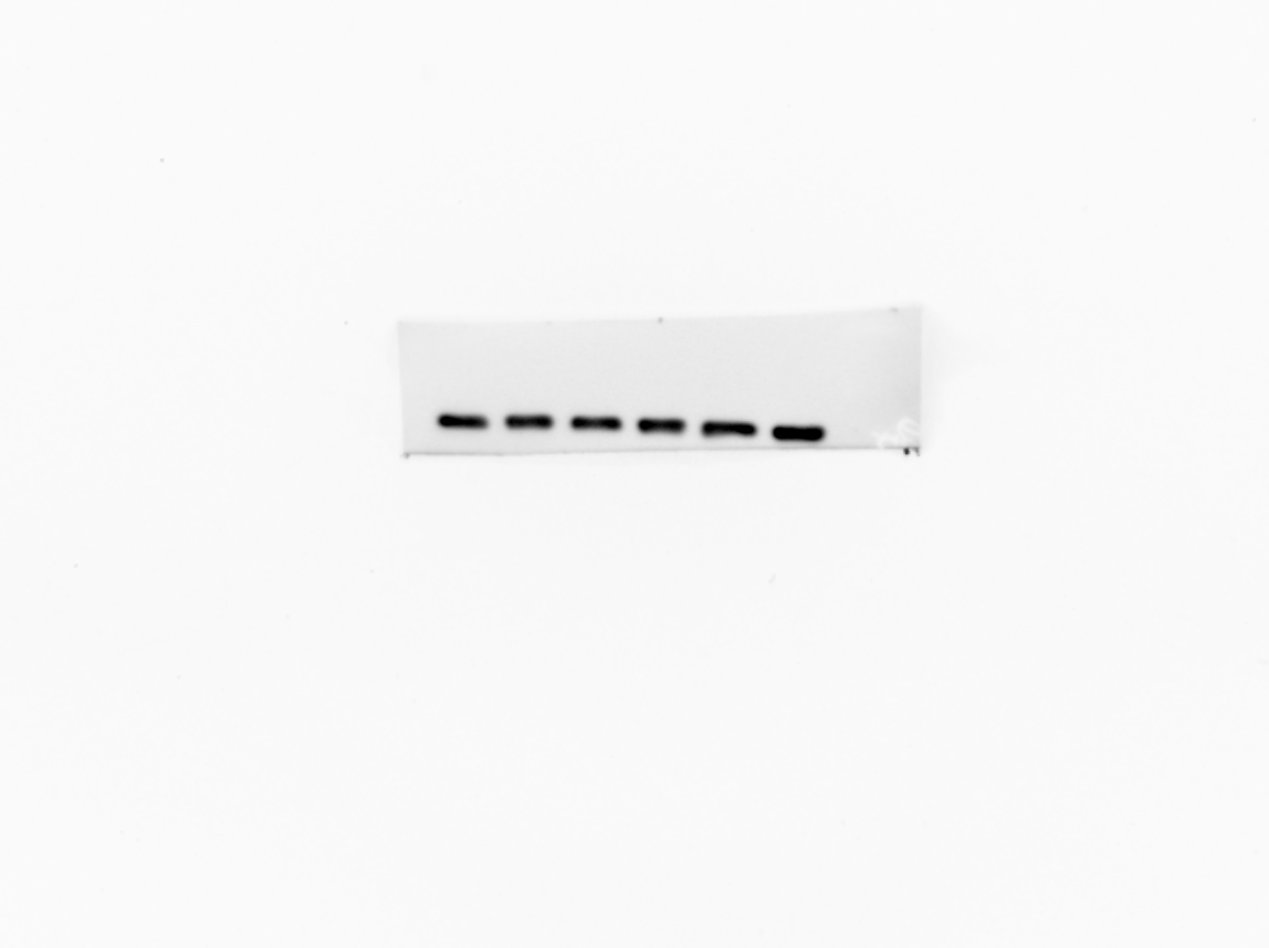
**

**Parp1**

**
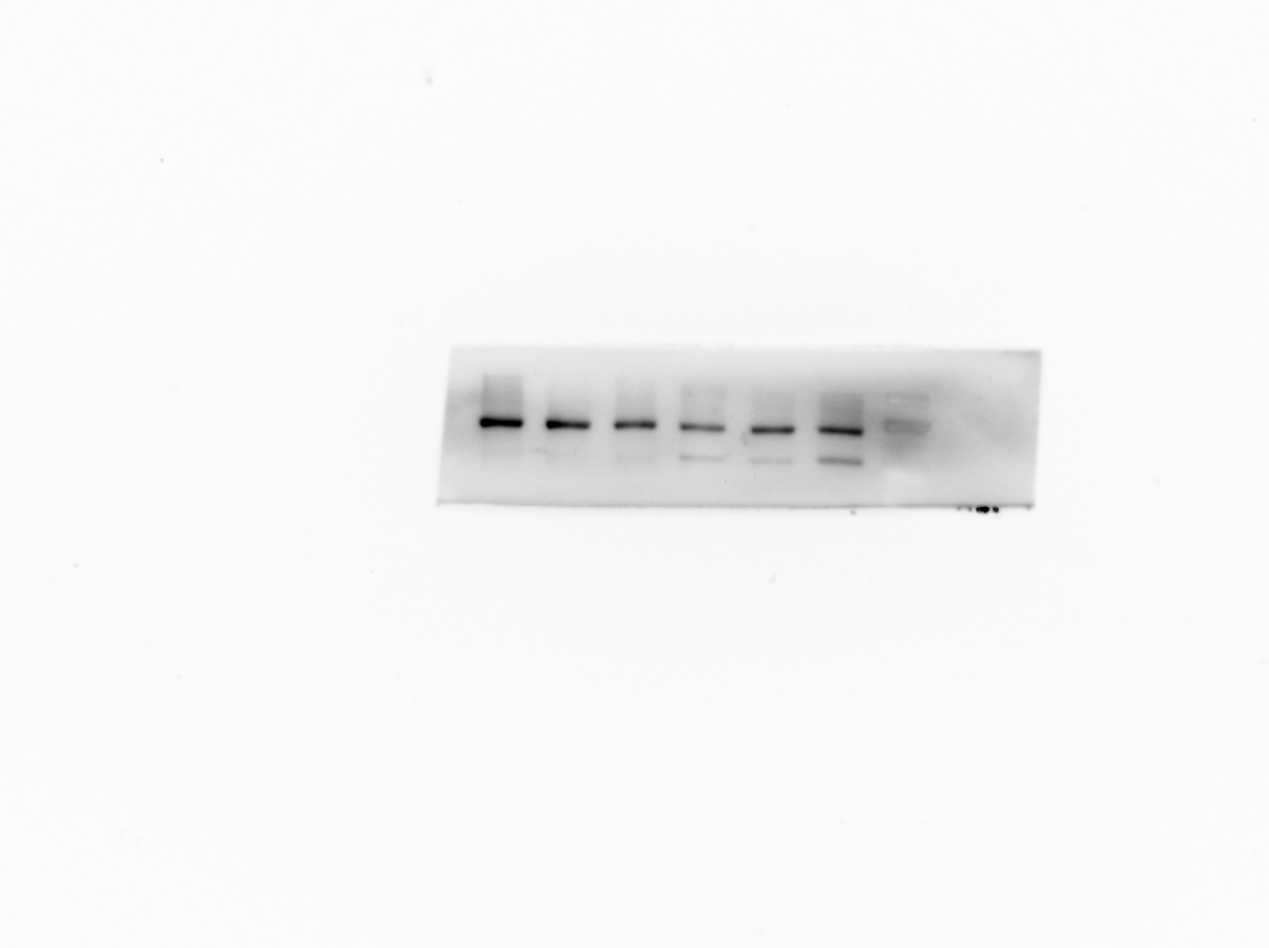
**

**β-Actin**

**
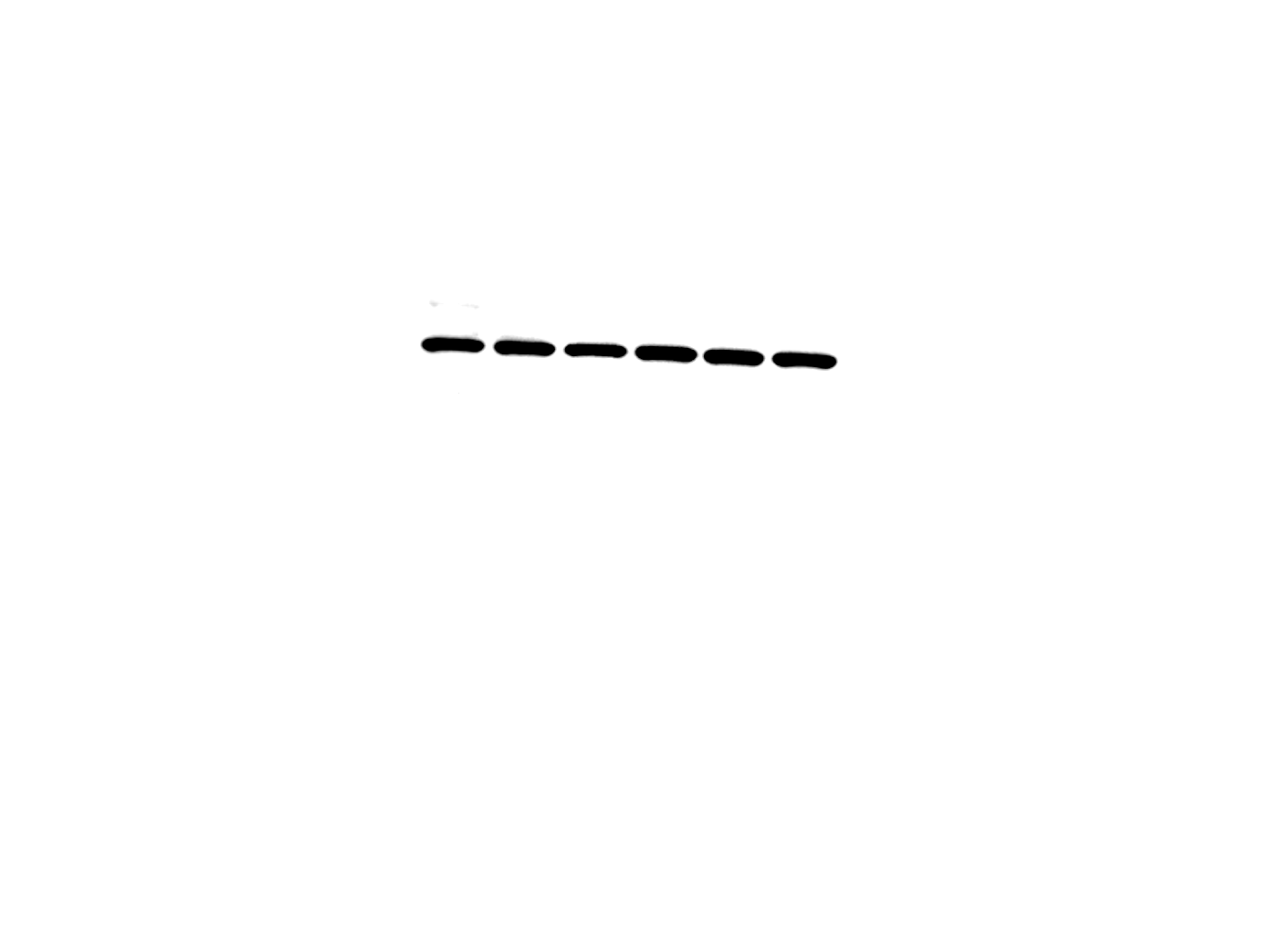
**

**Fig4.A**

**ATF4**

**
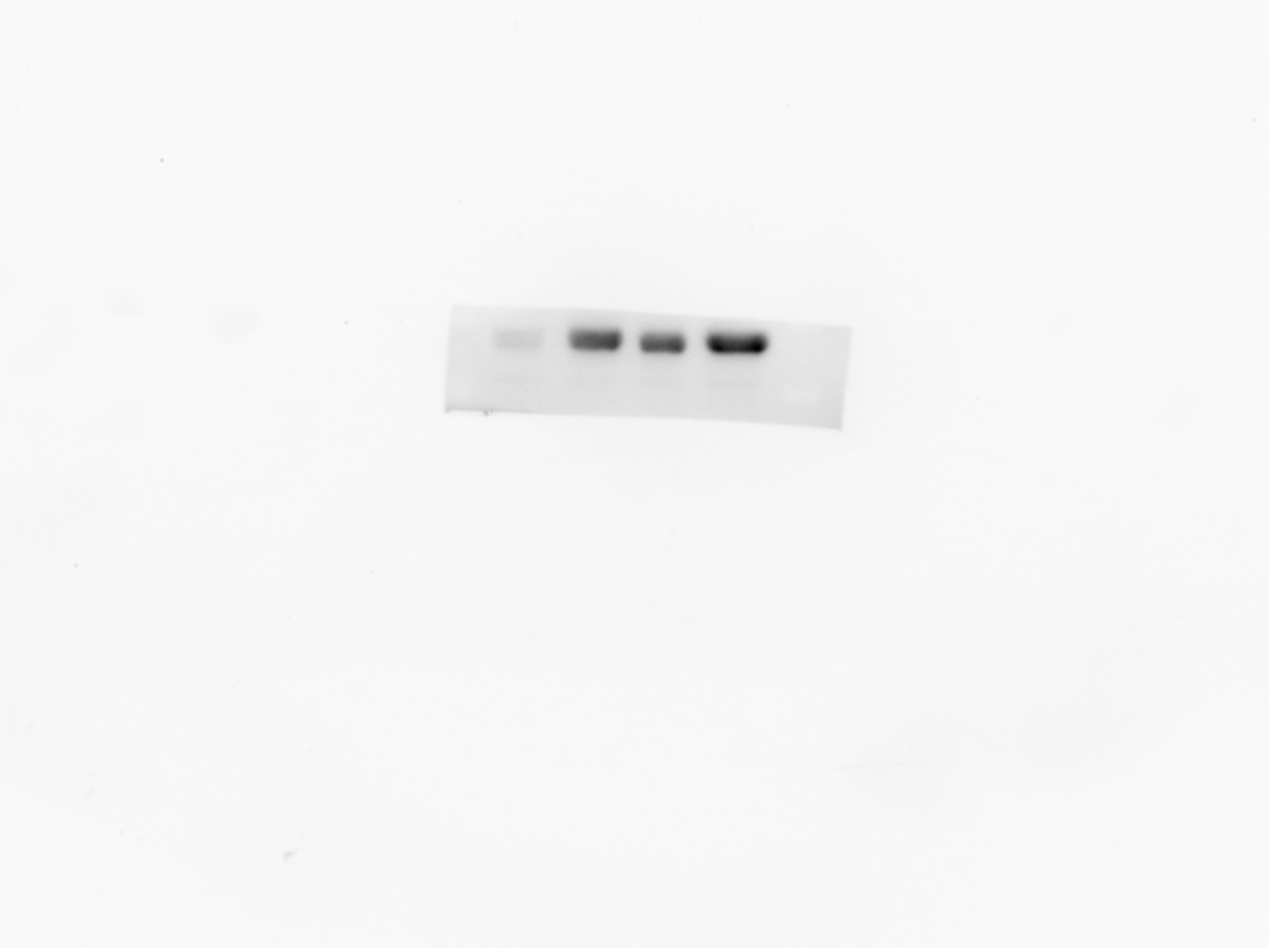
**

**Actin**

**
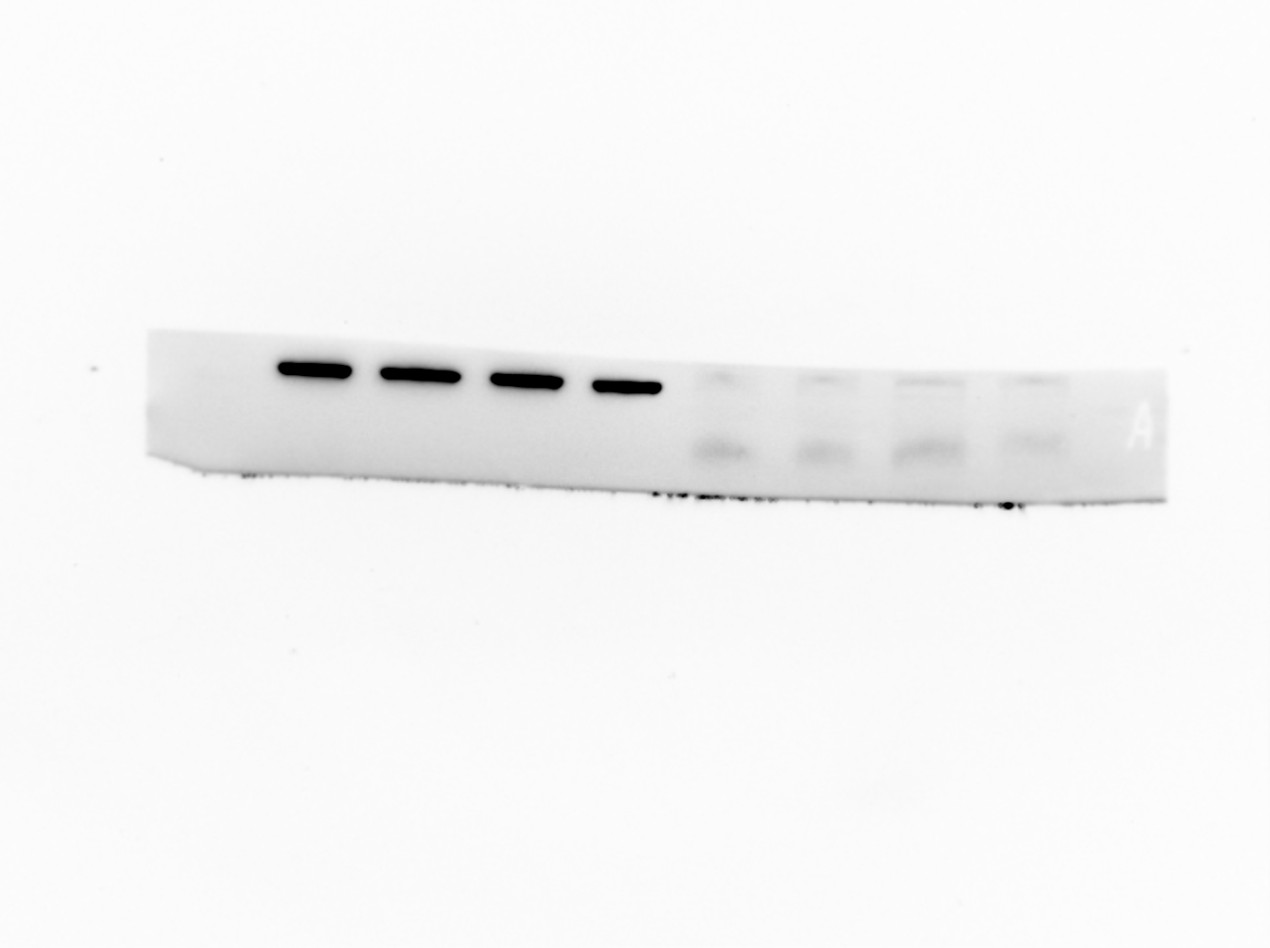
**

**Fig4.D**

**ATF4**

**
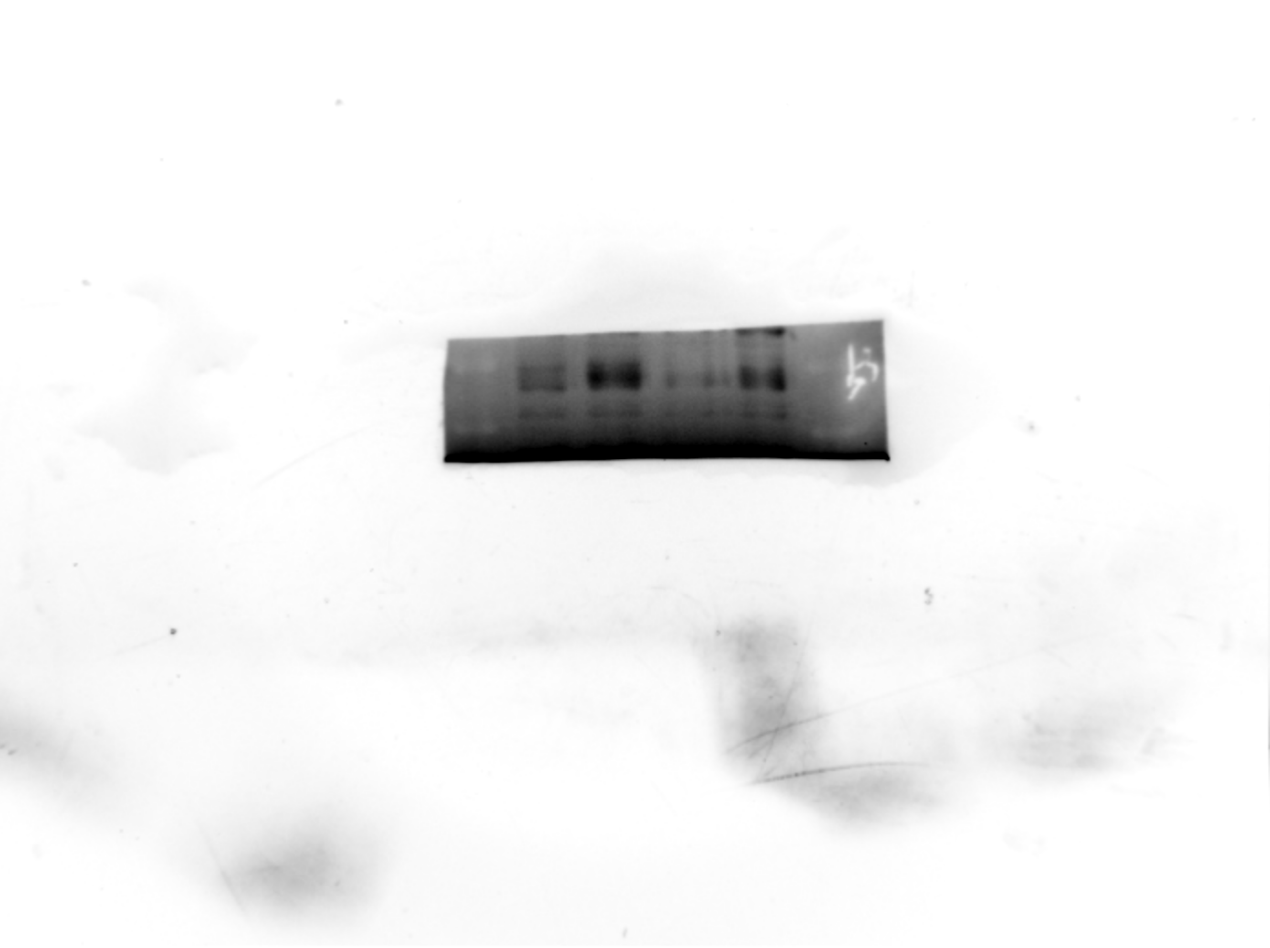
**

**SPHK1（U87MG-LN229）**

**
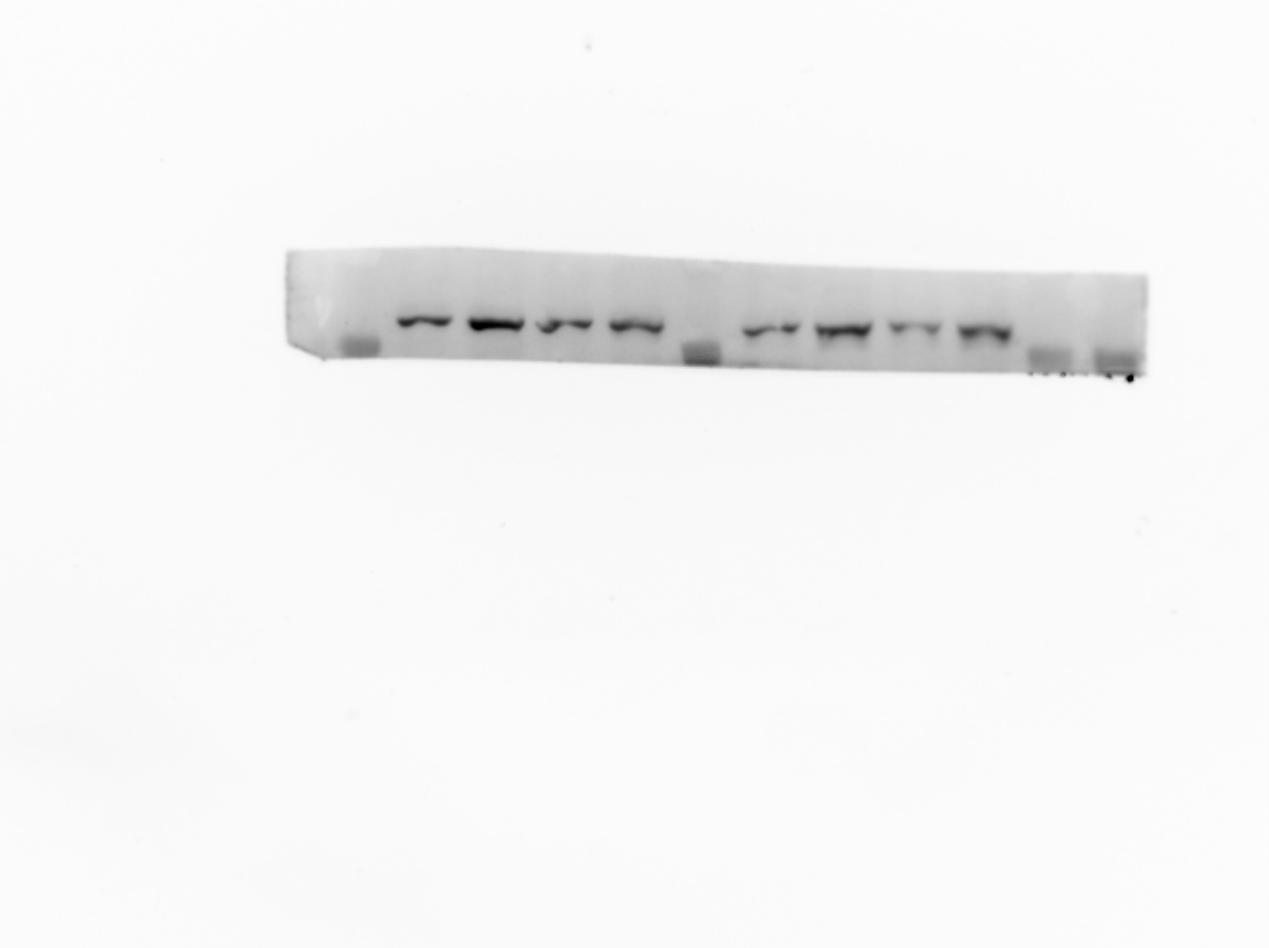
**

**β-Actin**

**
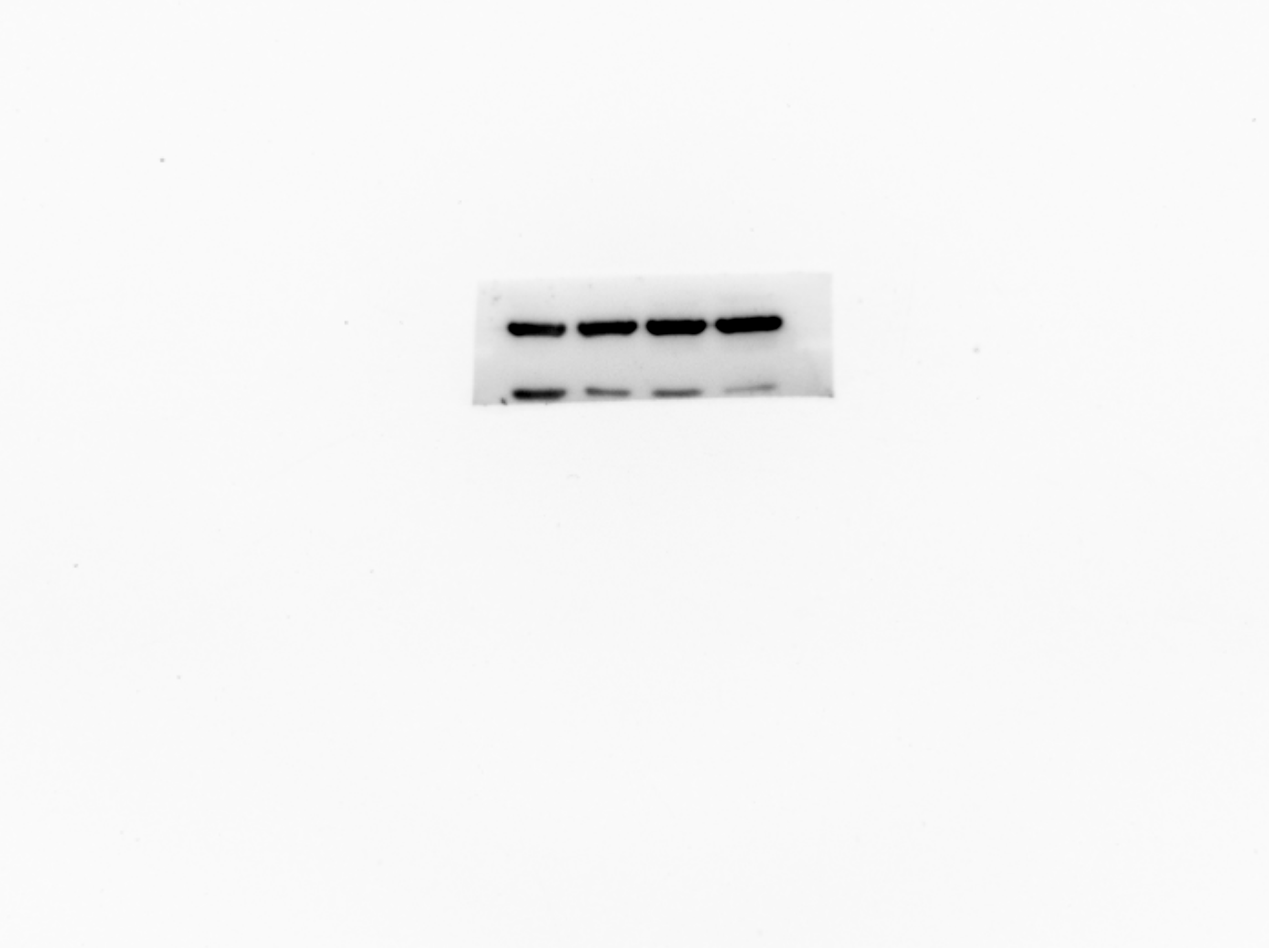
**

**Fig5.A**

**SPHK1 （LN229）**

**
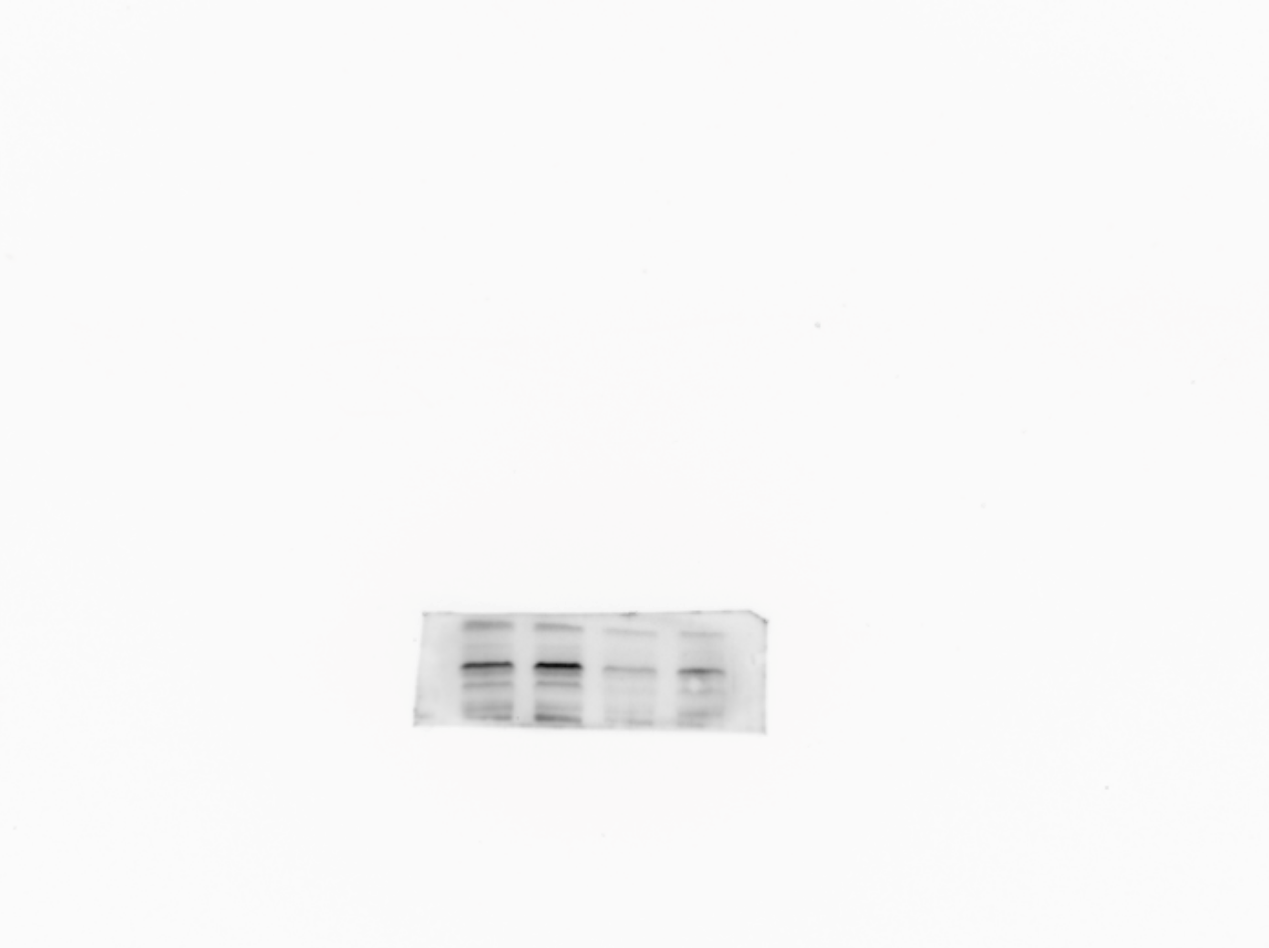
**

**β-Actin LN229（second）**

**
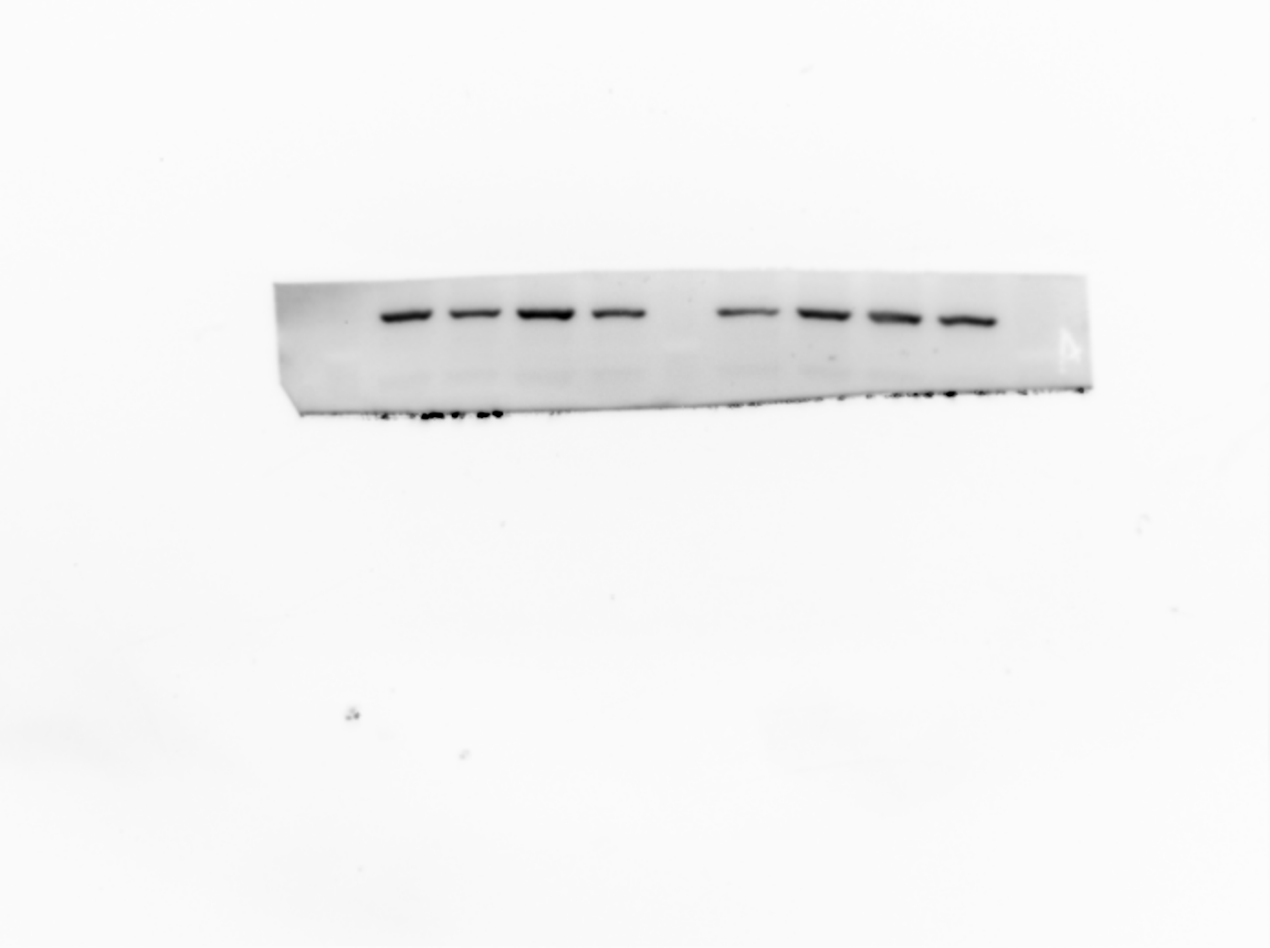
**

**SPHK1（U87MG）**

**
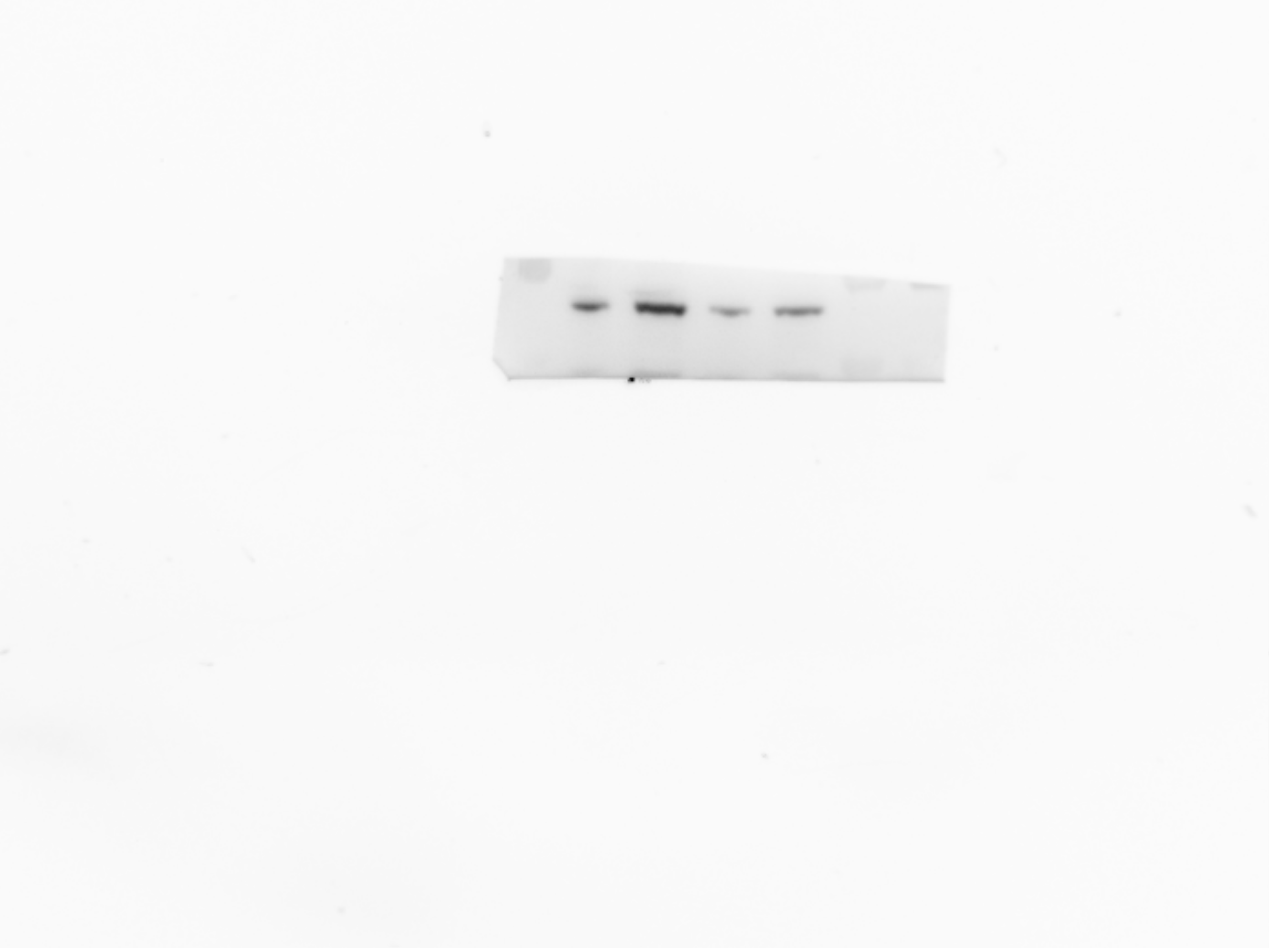
**

**Actin（U87MG）**

**
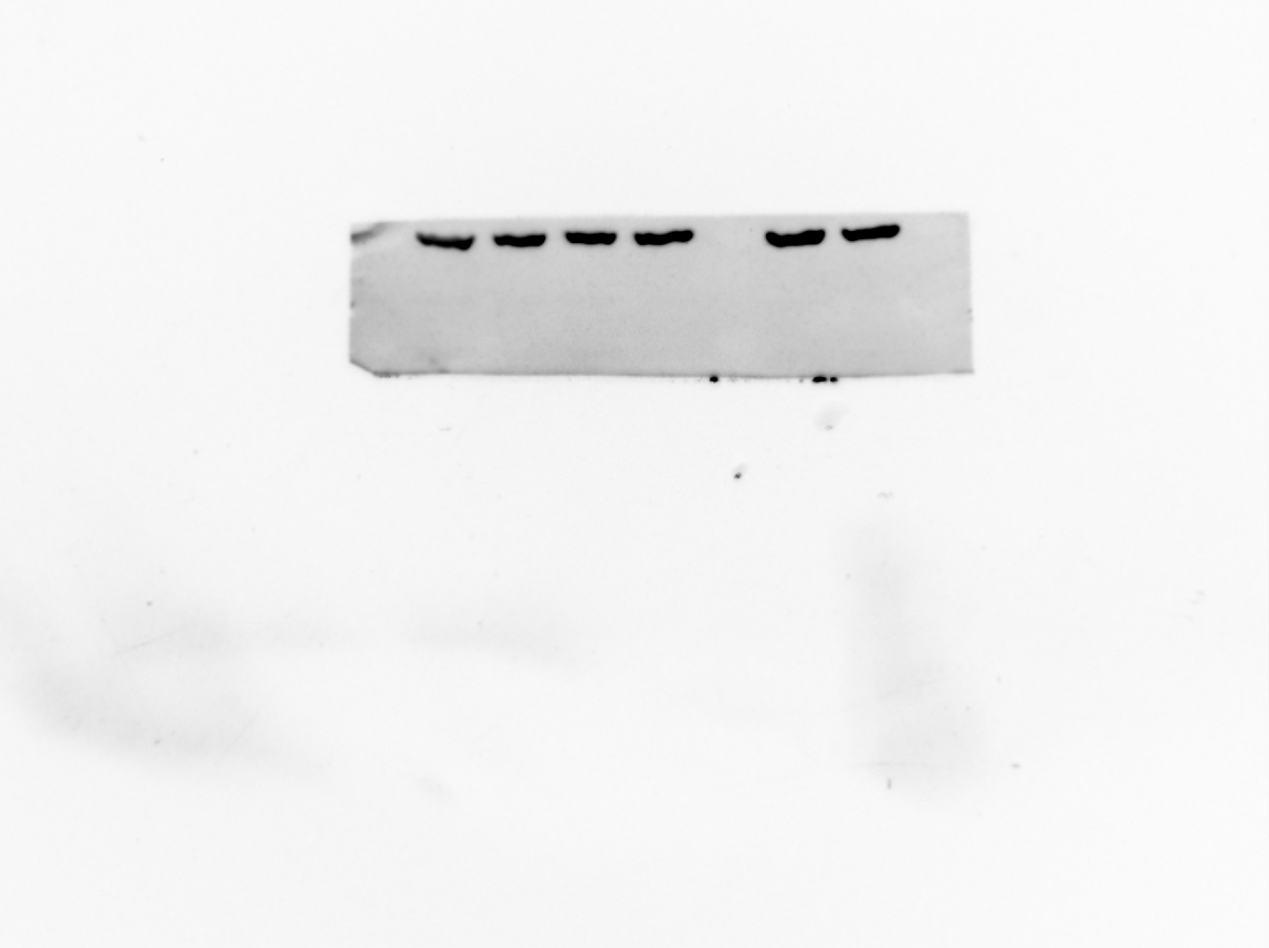
**

**Fig6.D**

**ATF4**

**
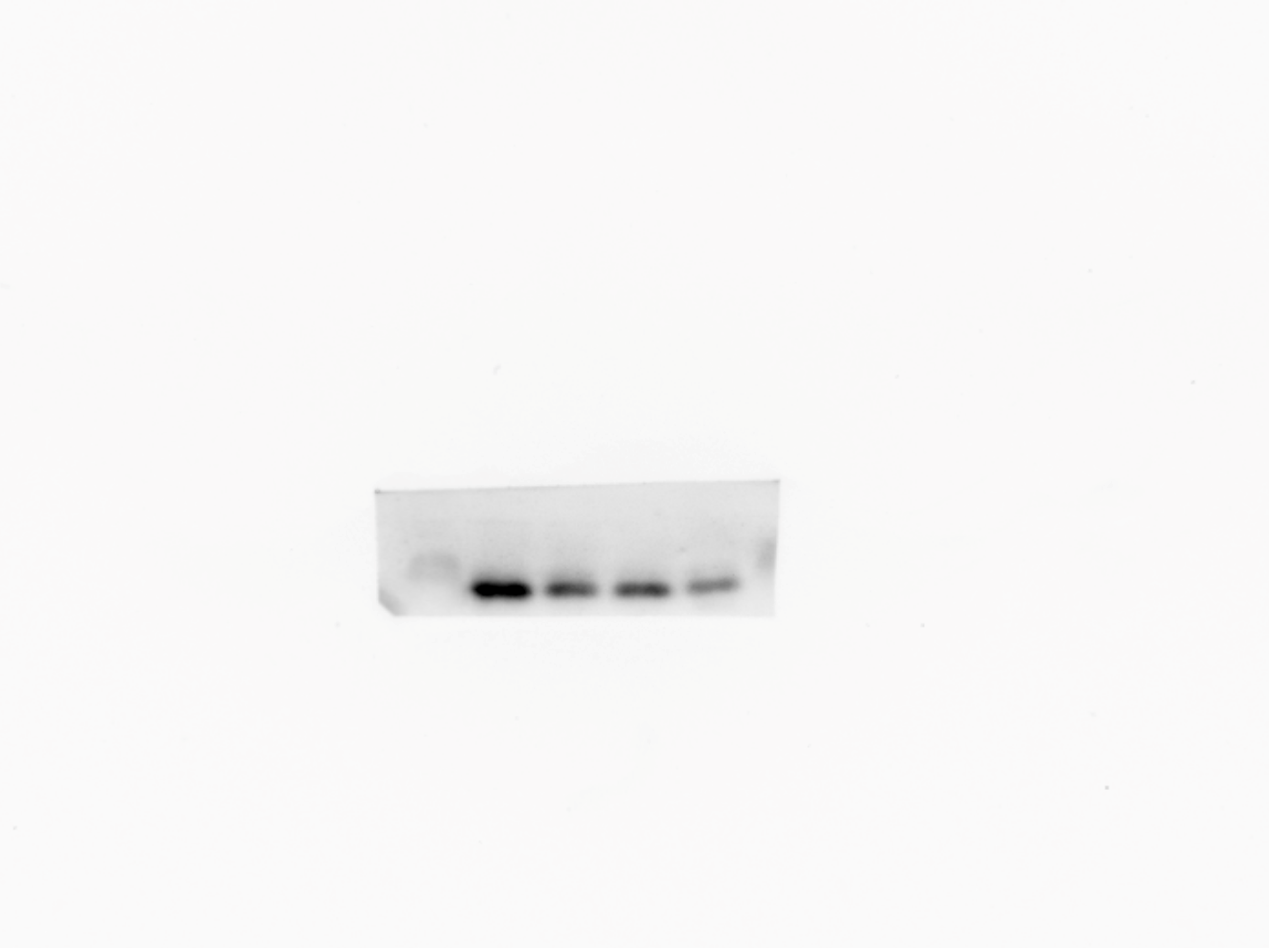
**

**SPHK1**

**
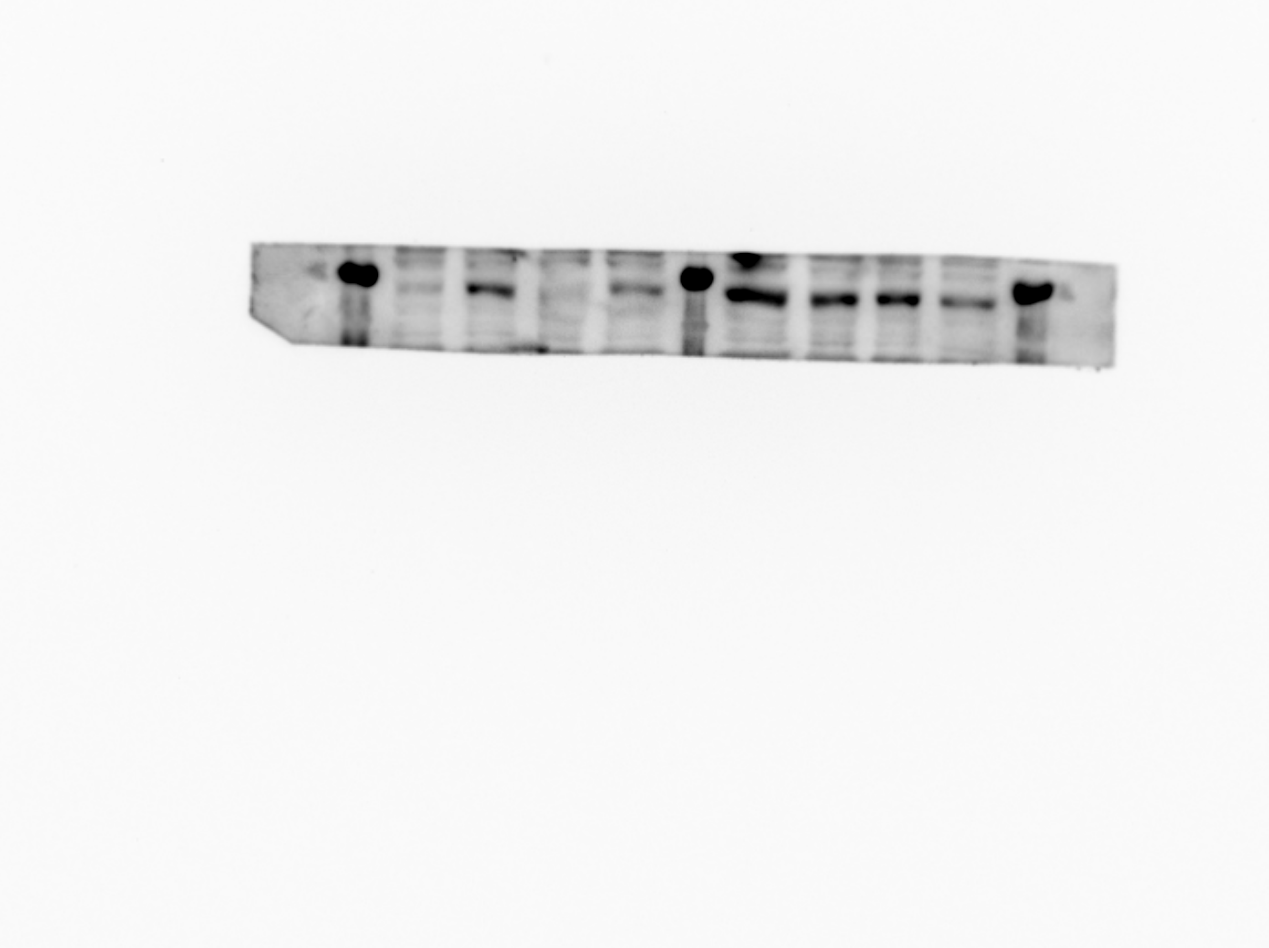
**

**Snail2**

**
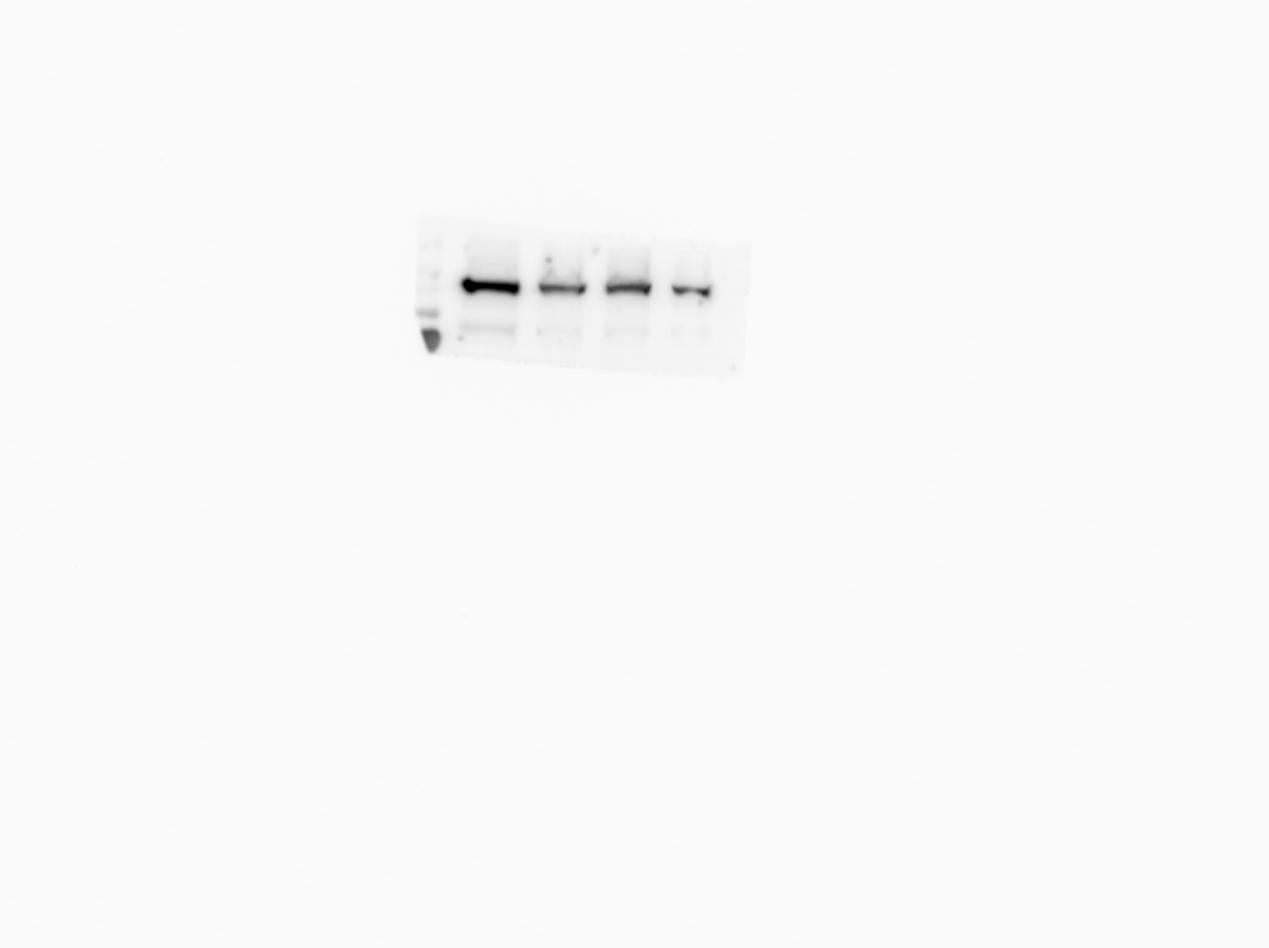
**

**E-cadherin**

**
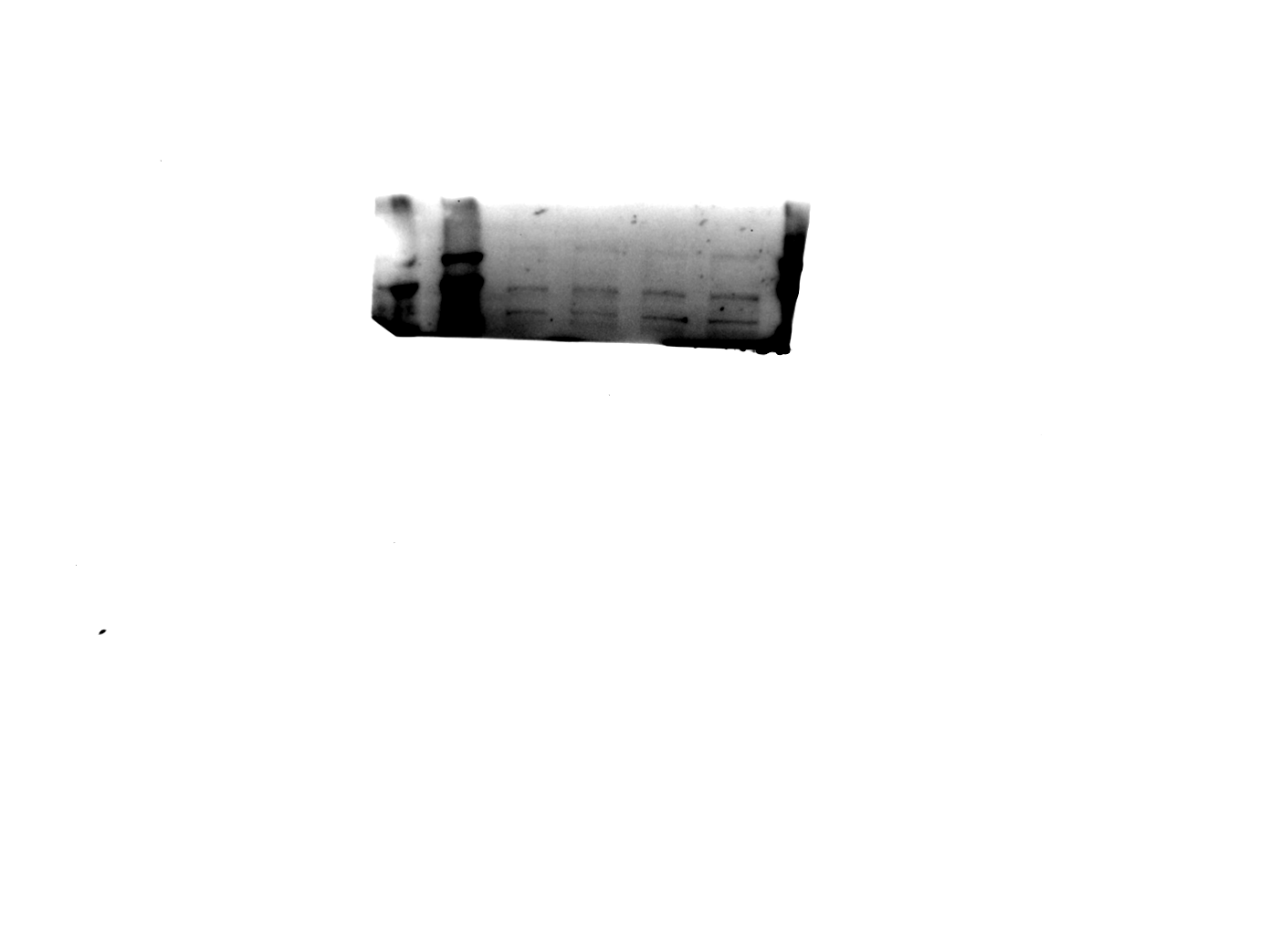
**

**N-cadherin**

**
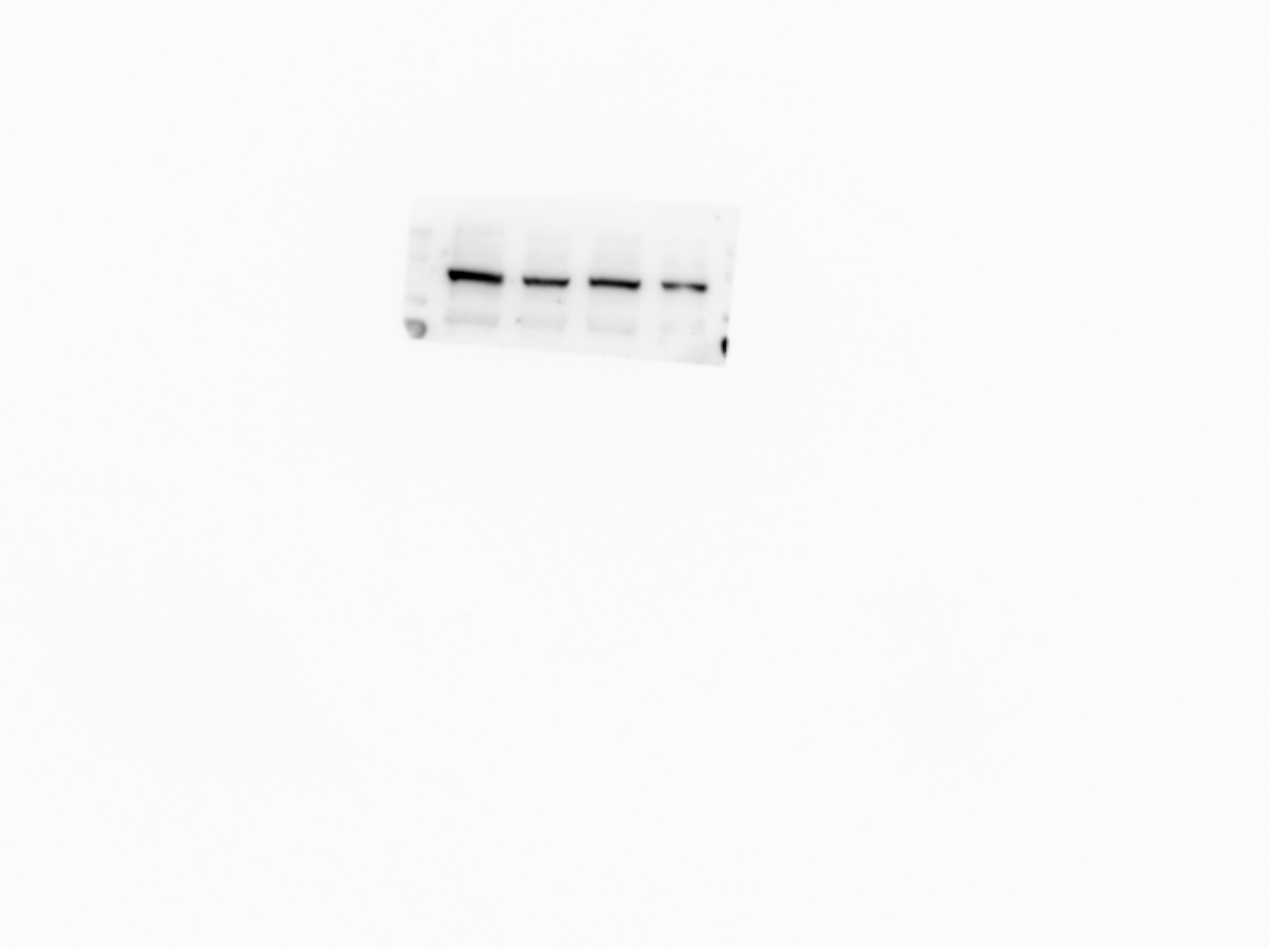
**

**Vimentin-U87MG-LN229**

**
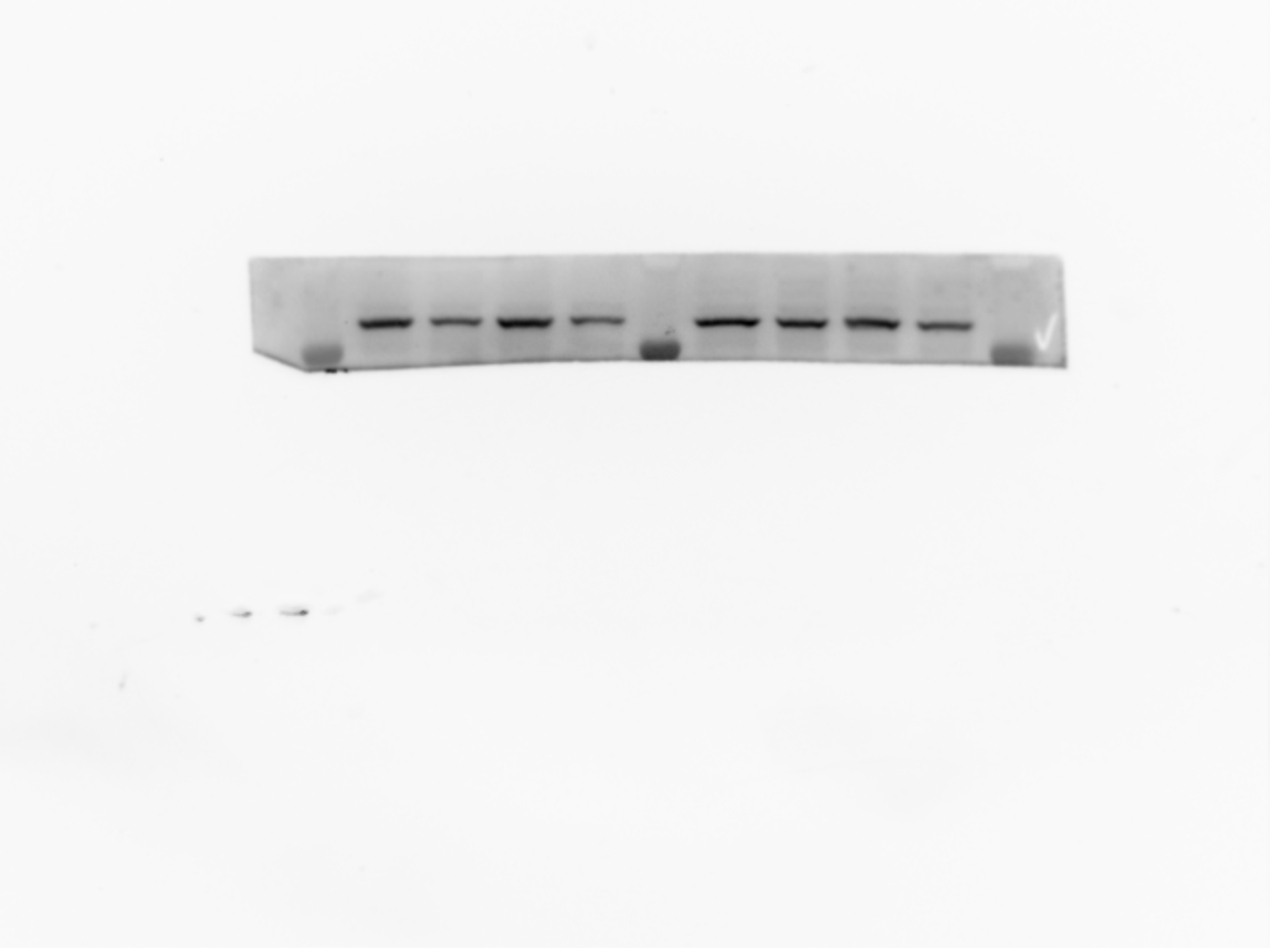
**

**β-Actin-U87MG-LN229**

**
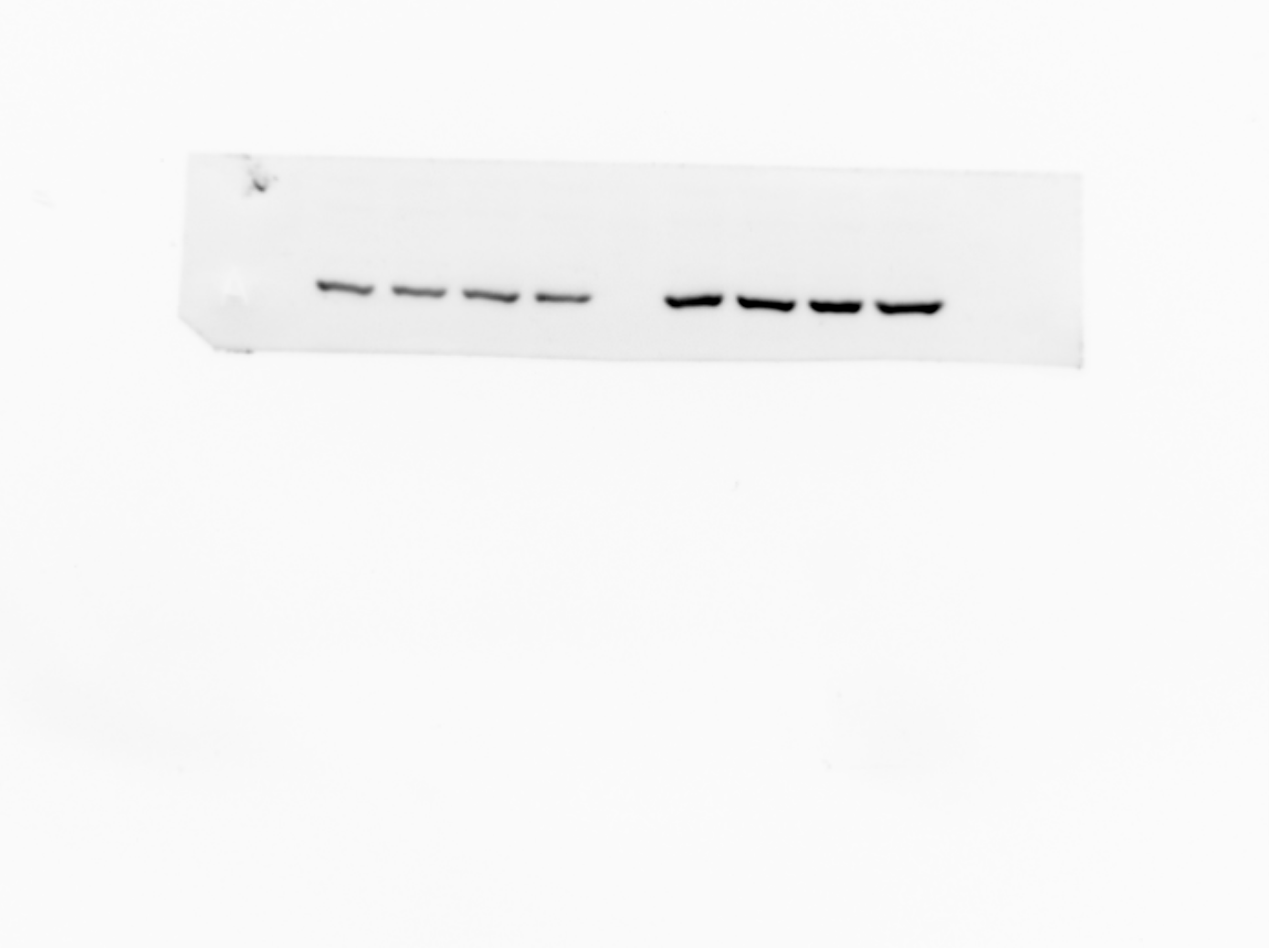
**

**Fig7.C**

**ATF4**





**SPHK1**





**β-Actin**


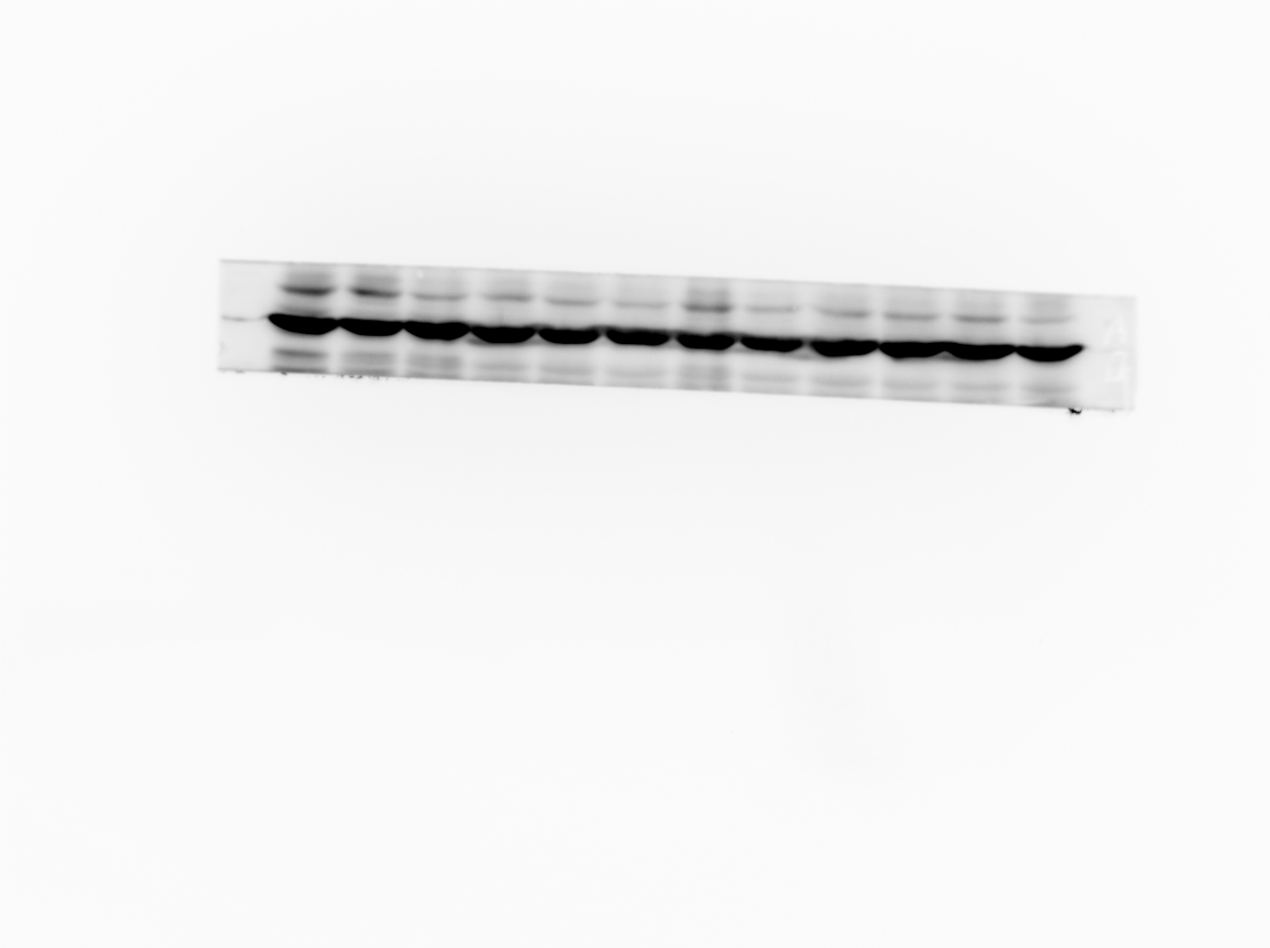


**Supplemental file**

**FigS2.A**

**Bip-U87MG**

**
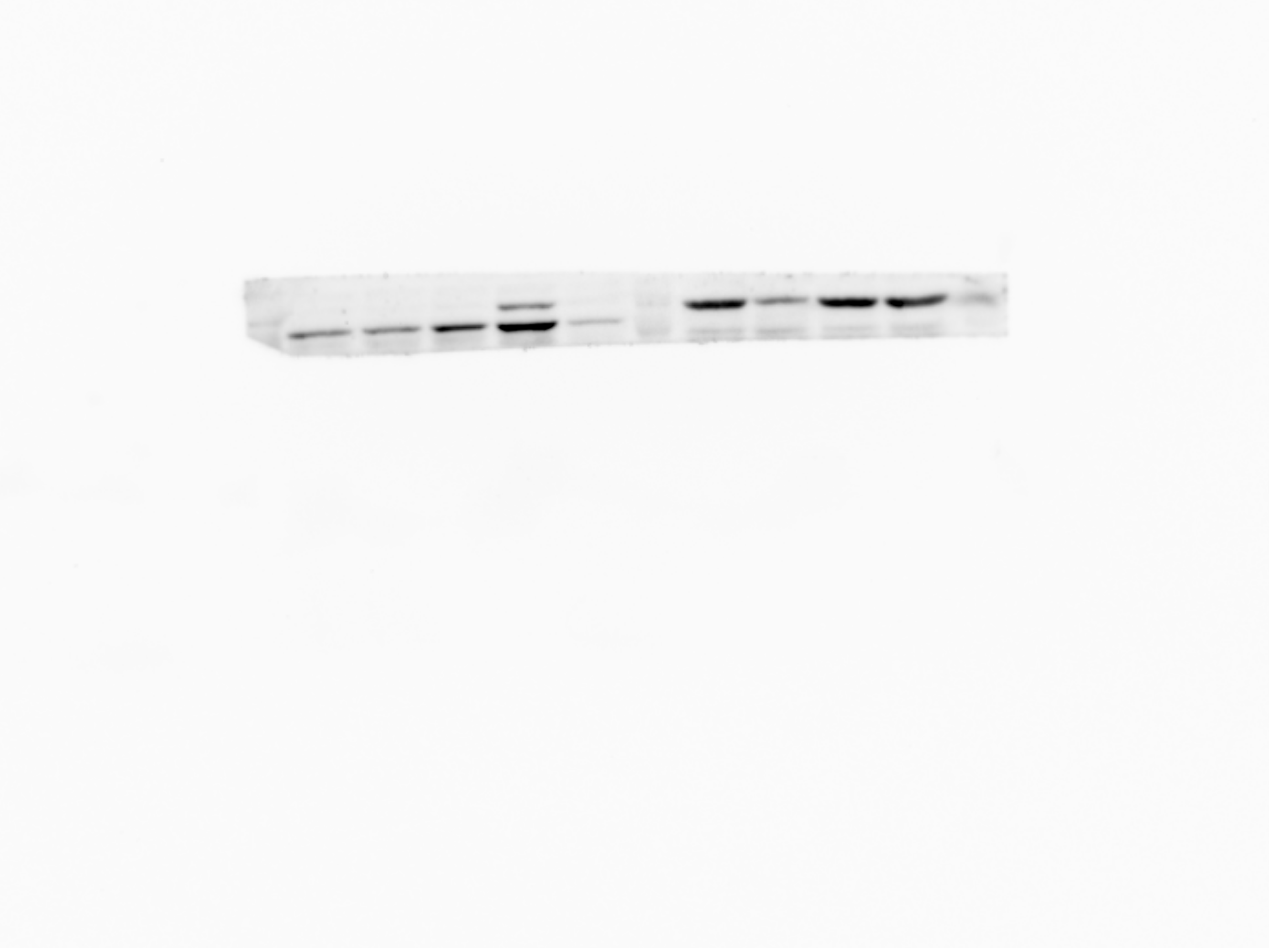
**

**p-PERK**

**
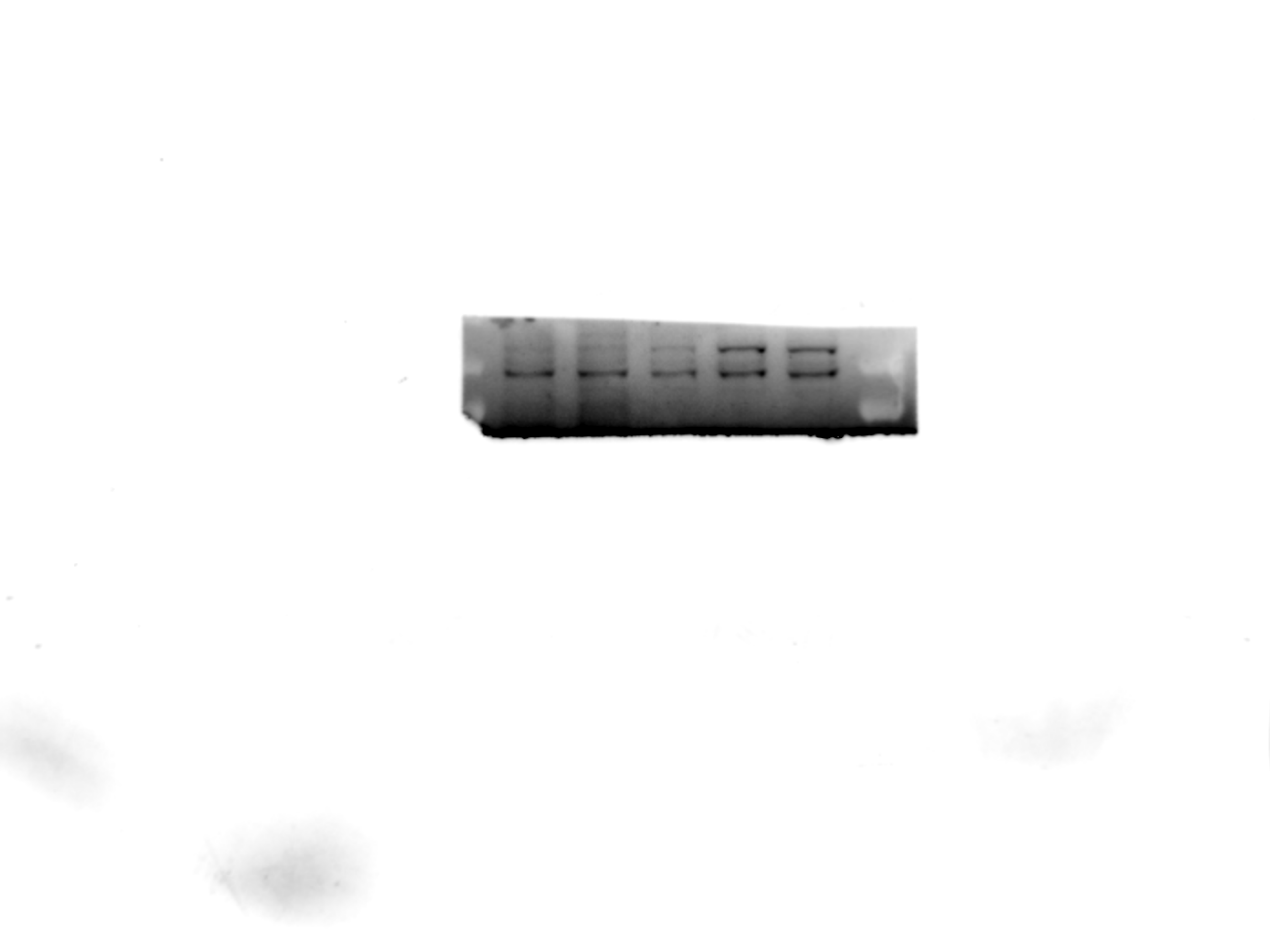
**

**p-eIF2α**

**
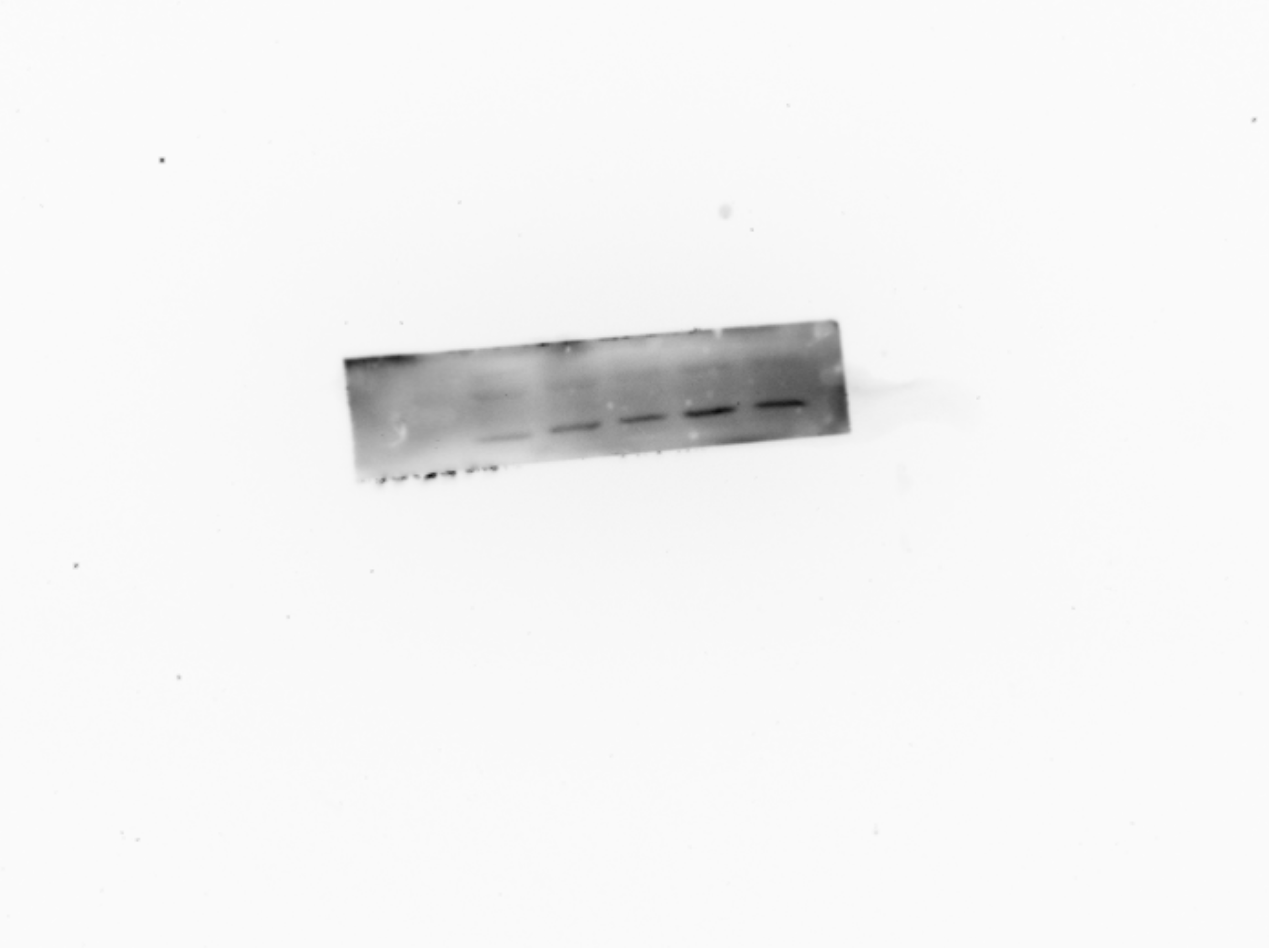
**

**eIF2α
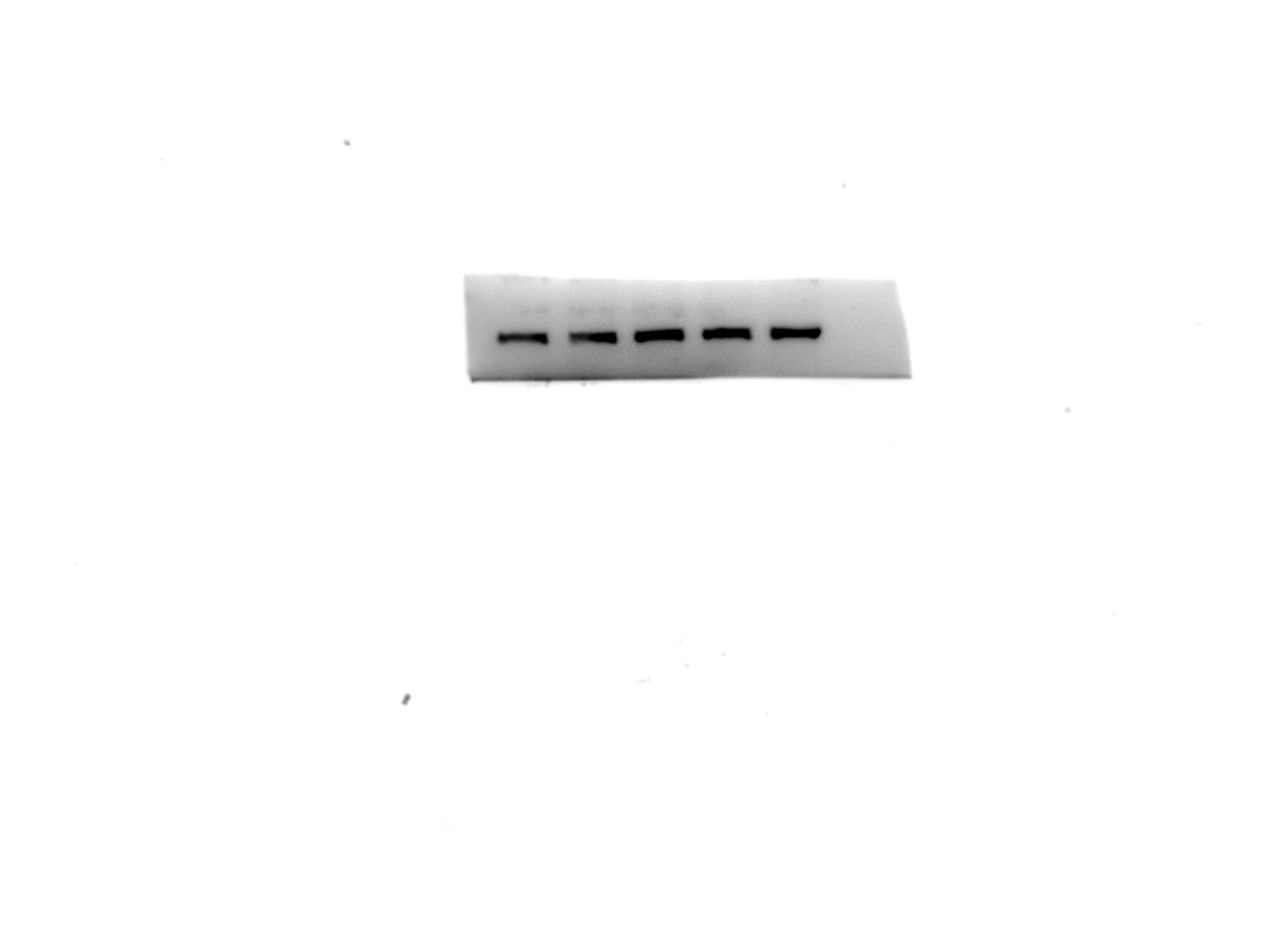
**

**ATF4**

**
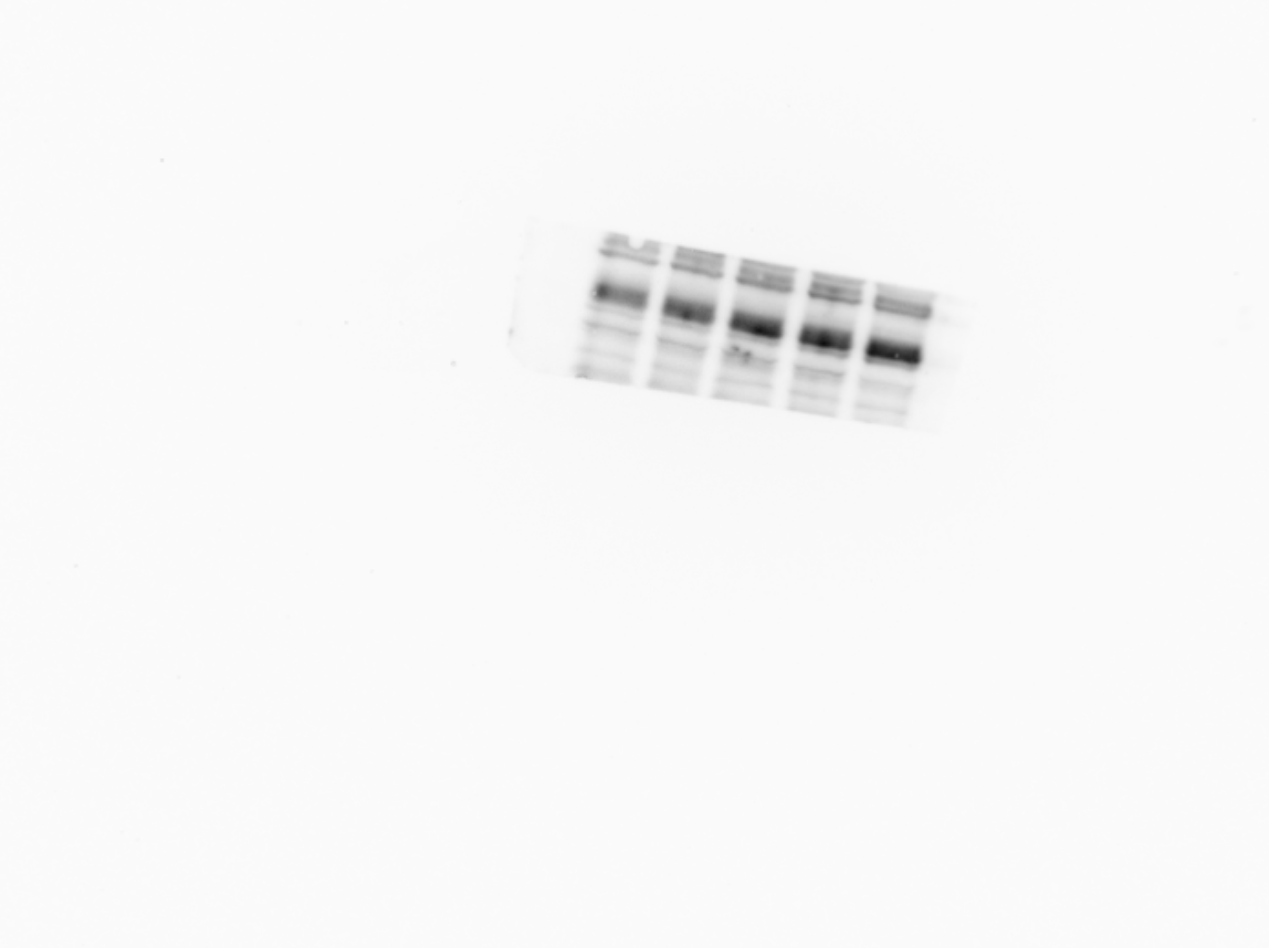
**

**β-Actin**

**
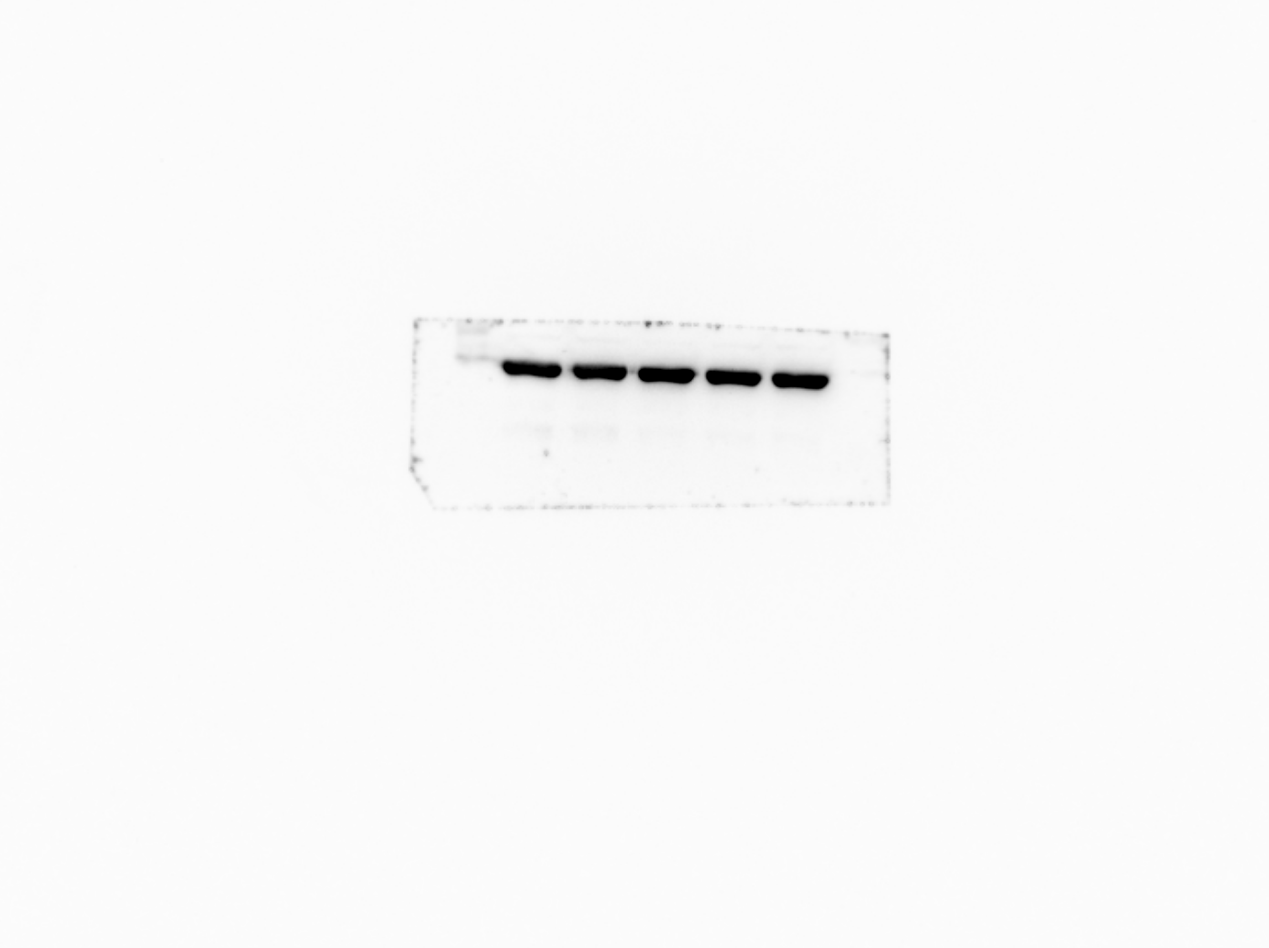
**

**FigS2.D**

**p-eIF2α**

**
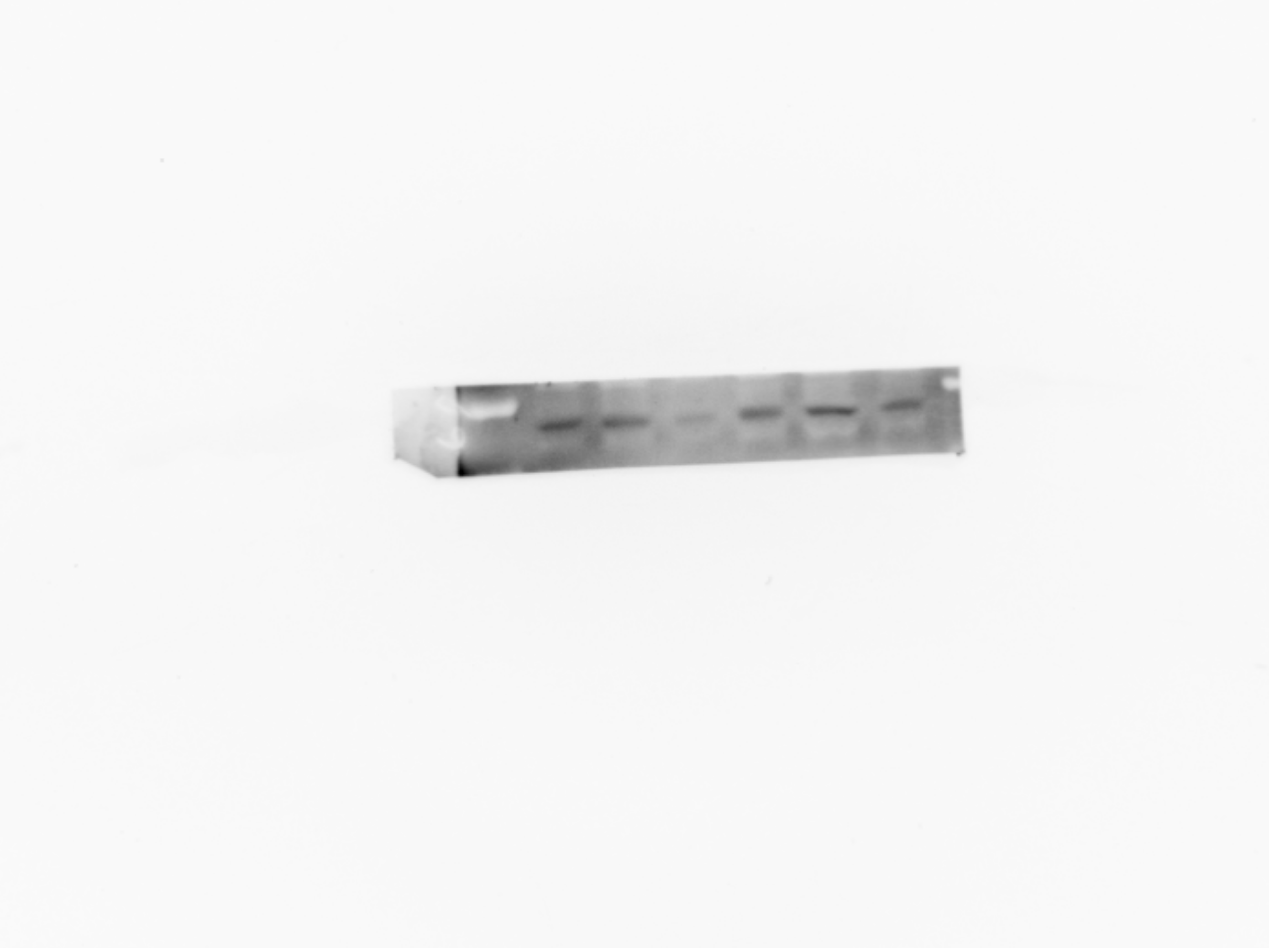
**

**eIF2α**

**
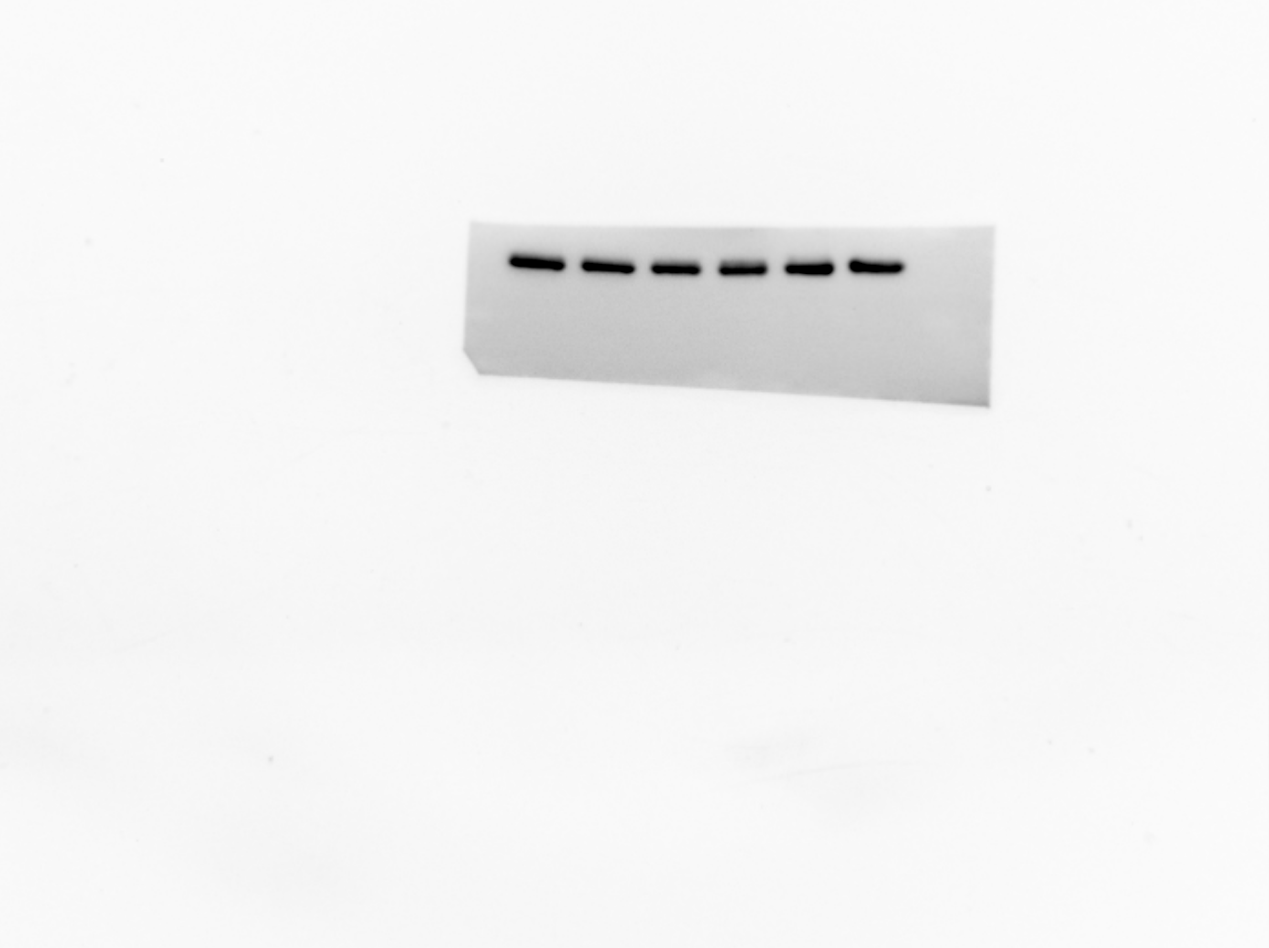
**

**ATF4**

**
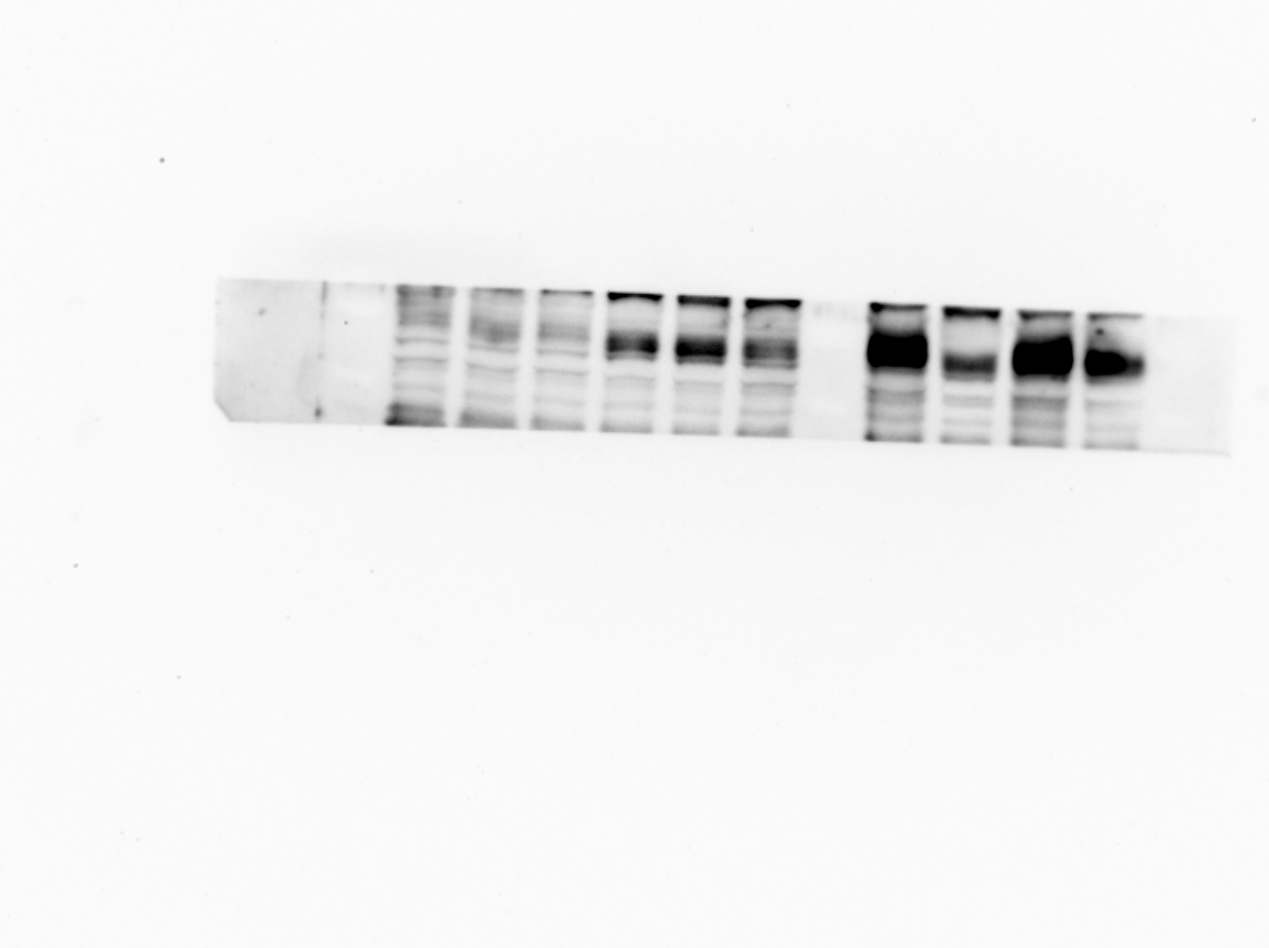
**

**Parp1**

**
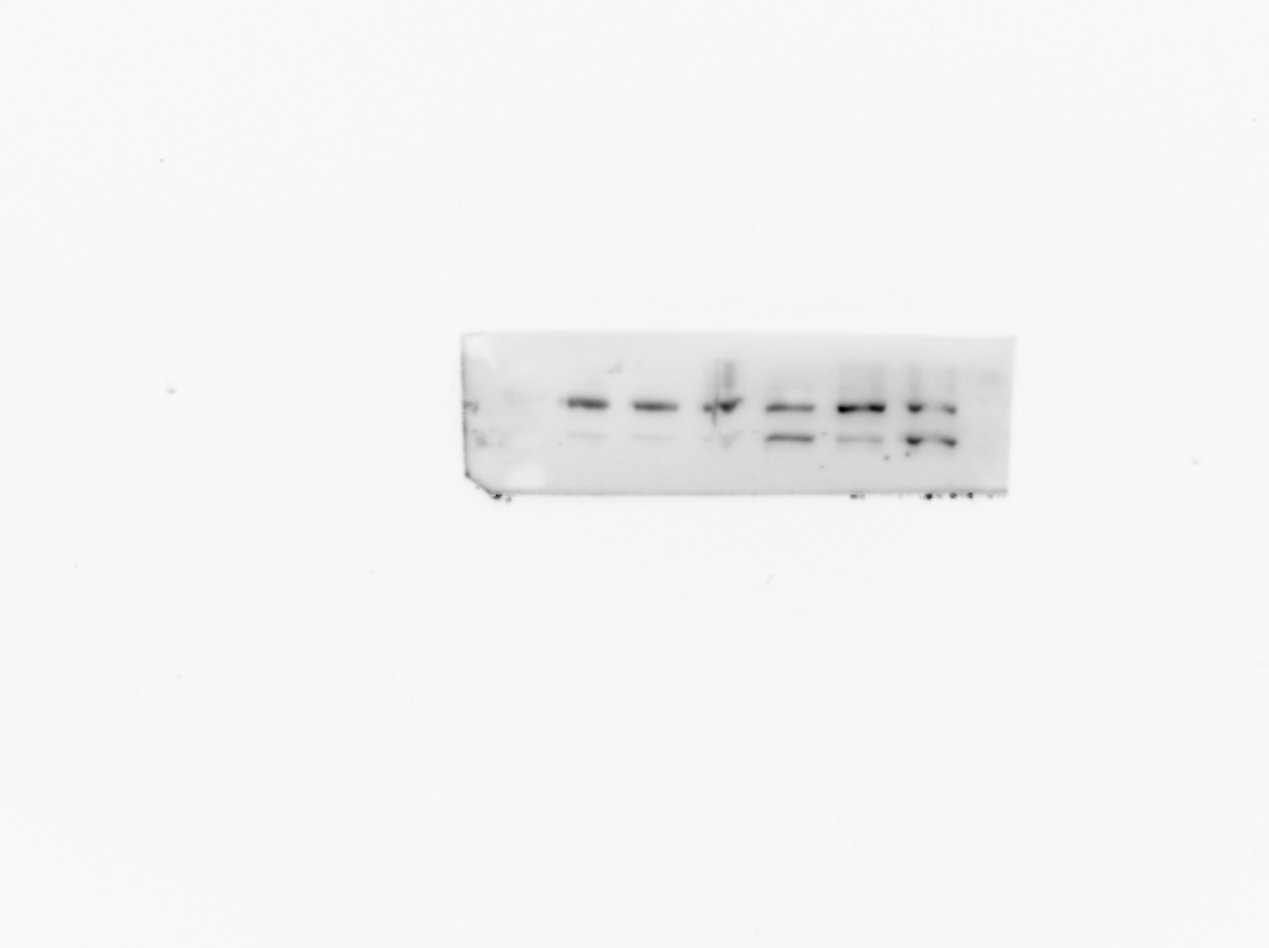
**

**Actin**

**
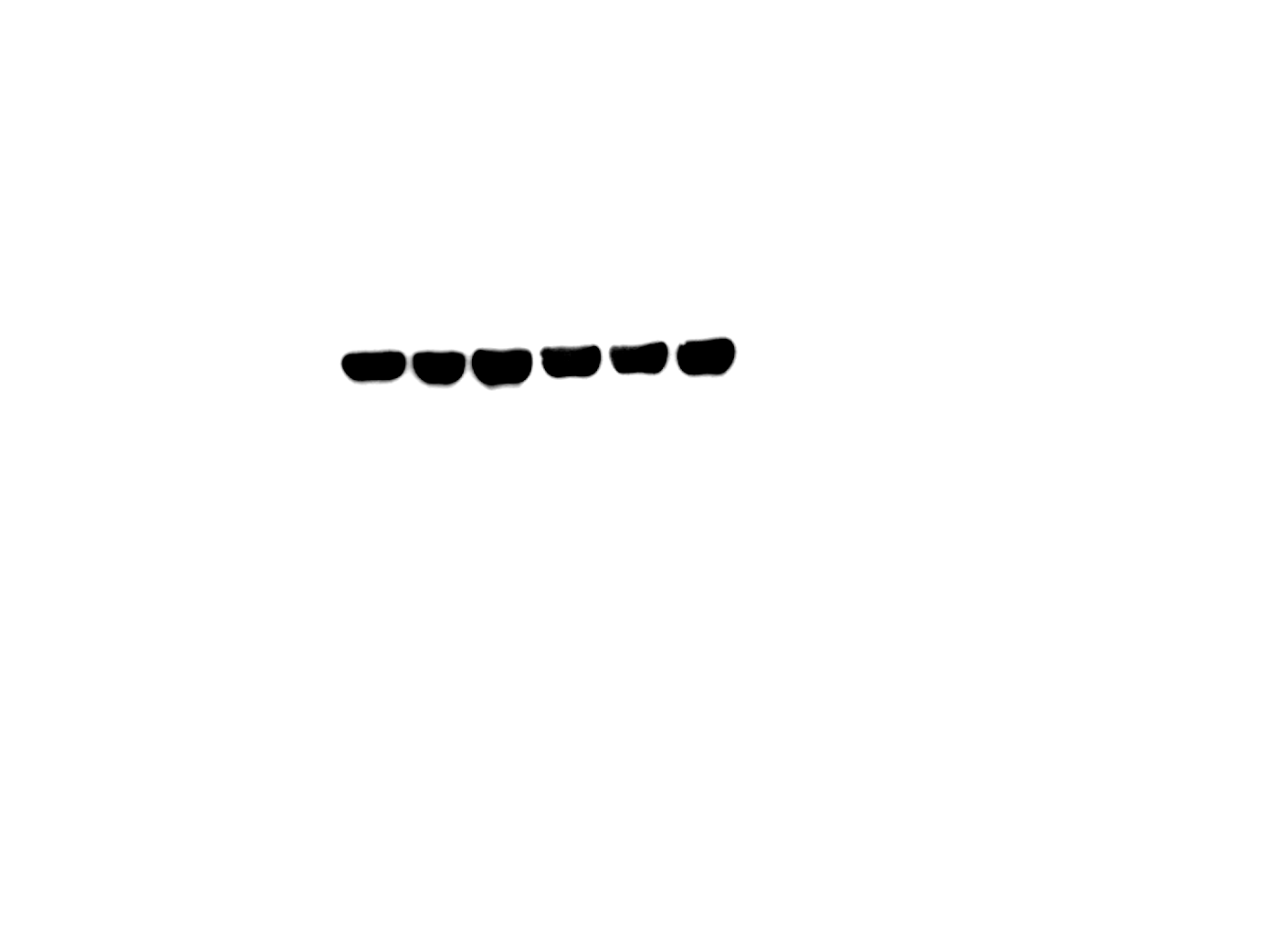
**

**FigS3.A**

**ATF4-LN229**

**

**

**Actin-LN229**

**
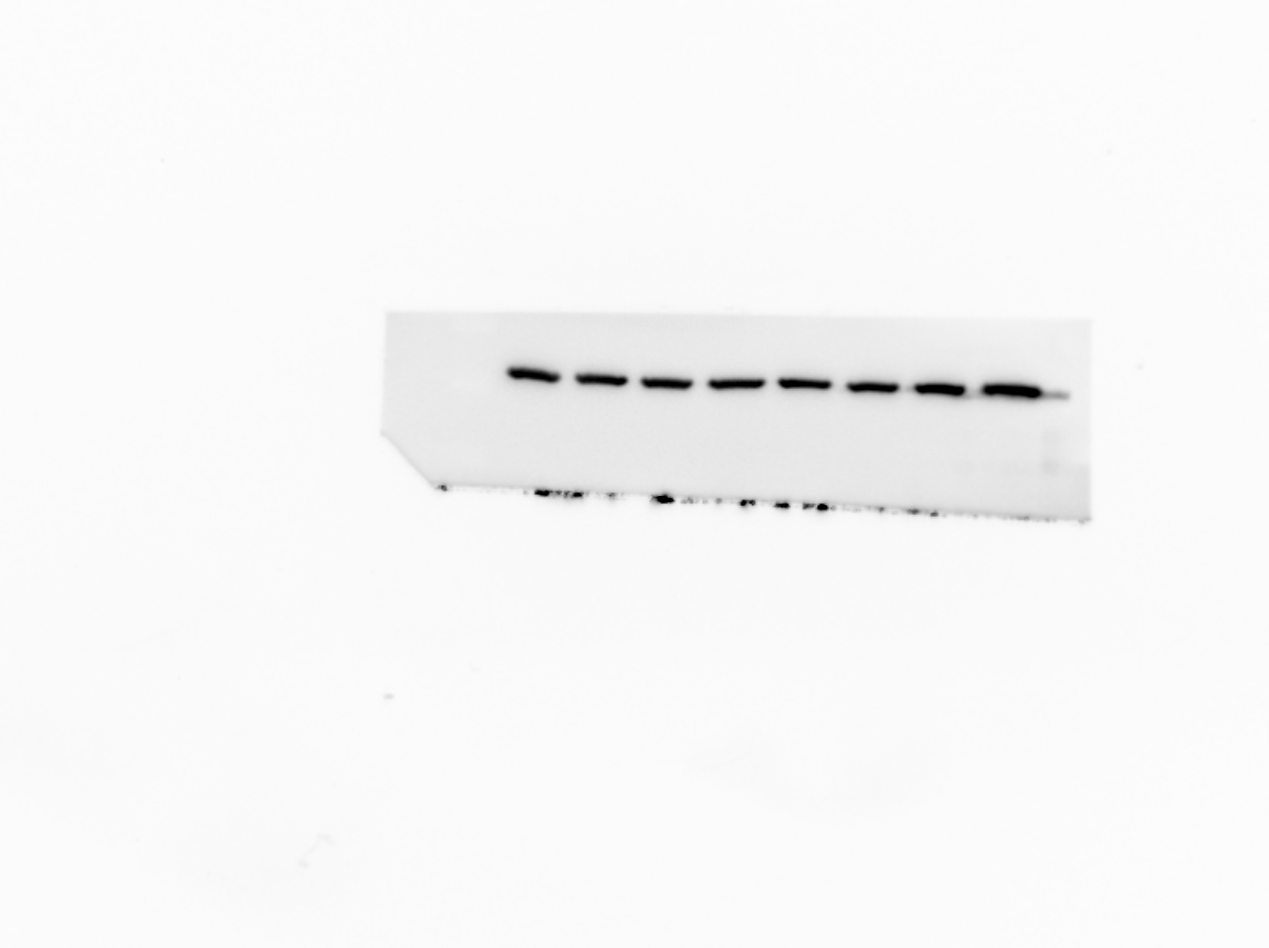
**

**ATF4-U87MG**

**
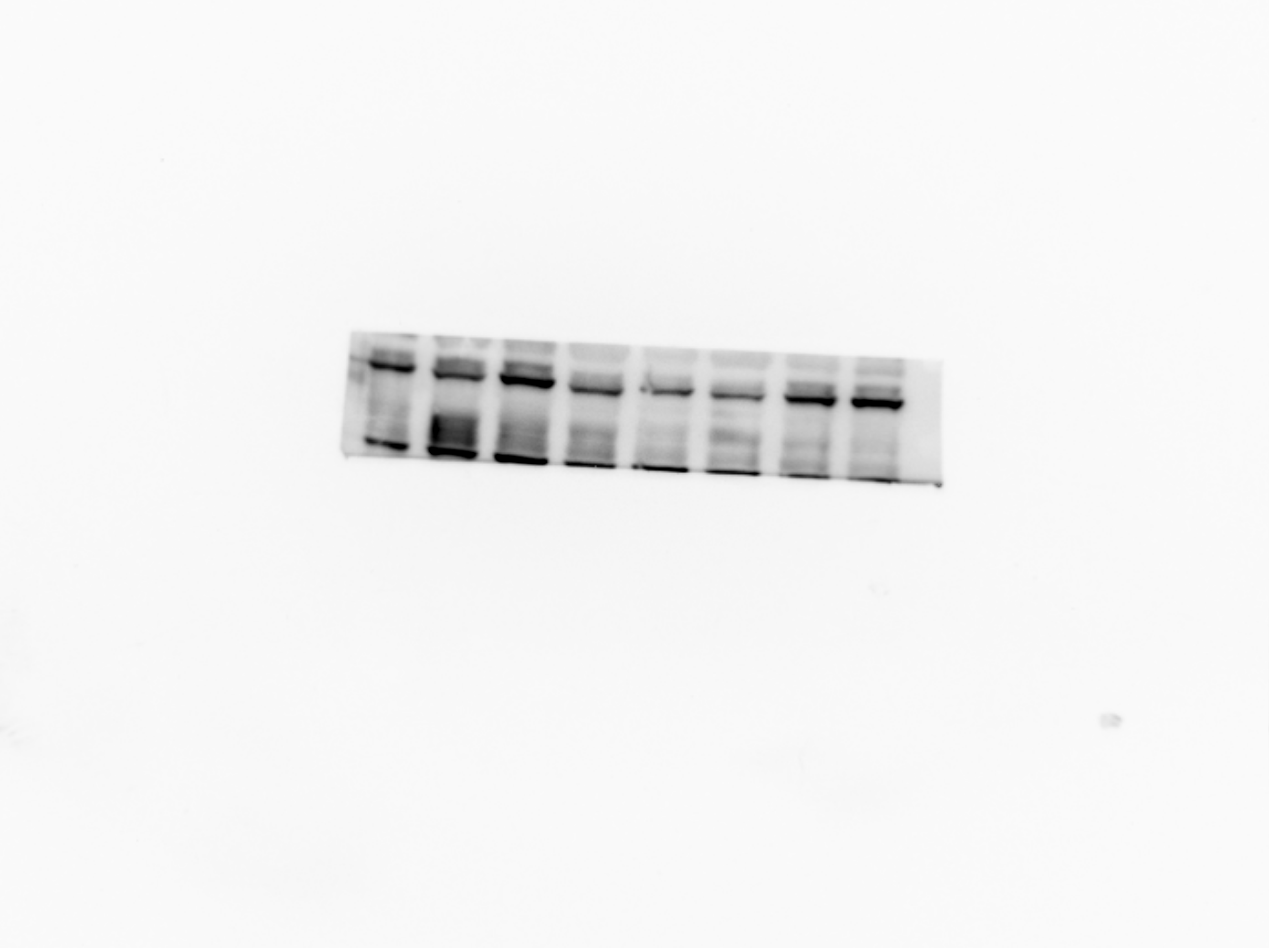
**

**β-actin-U87MG**

**
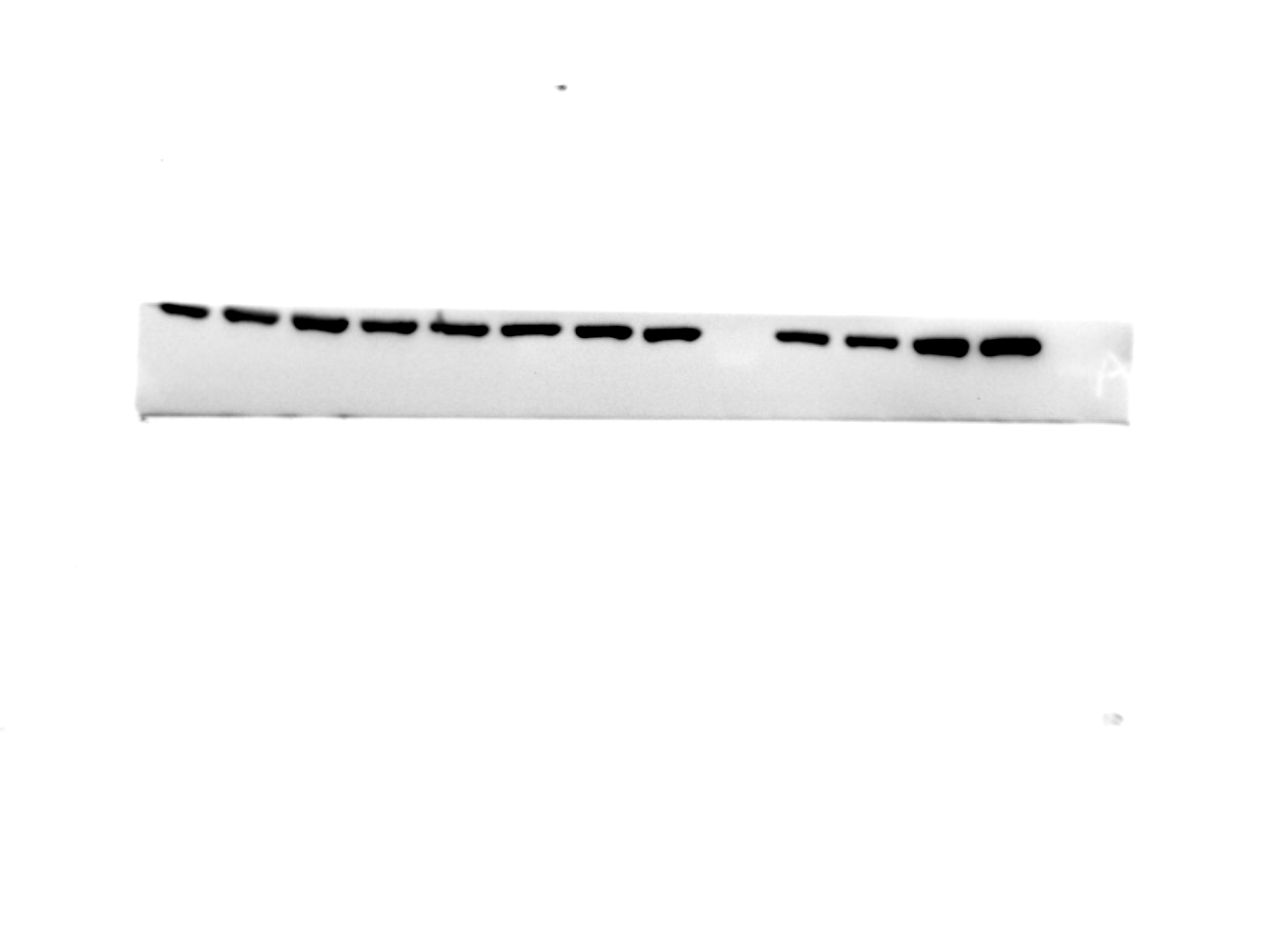
**

**FigS5.B**

**ATF4-U87MG**

**
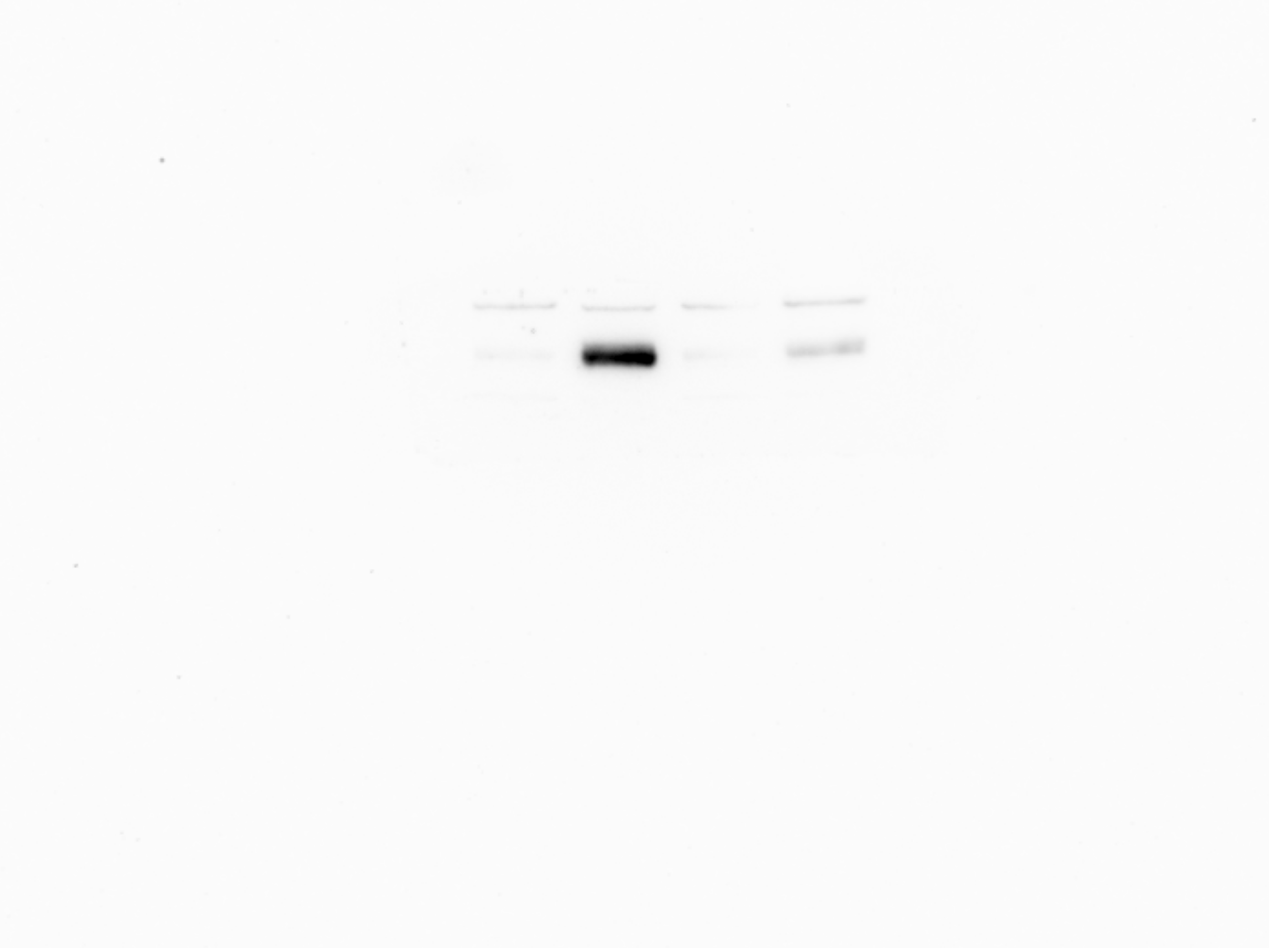
**

**SPHK1-U87MG-LN229**

**
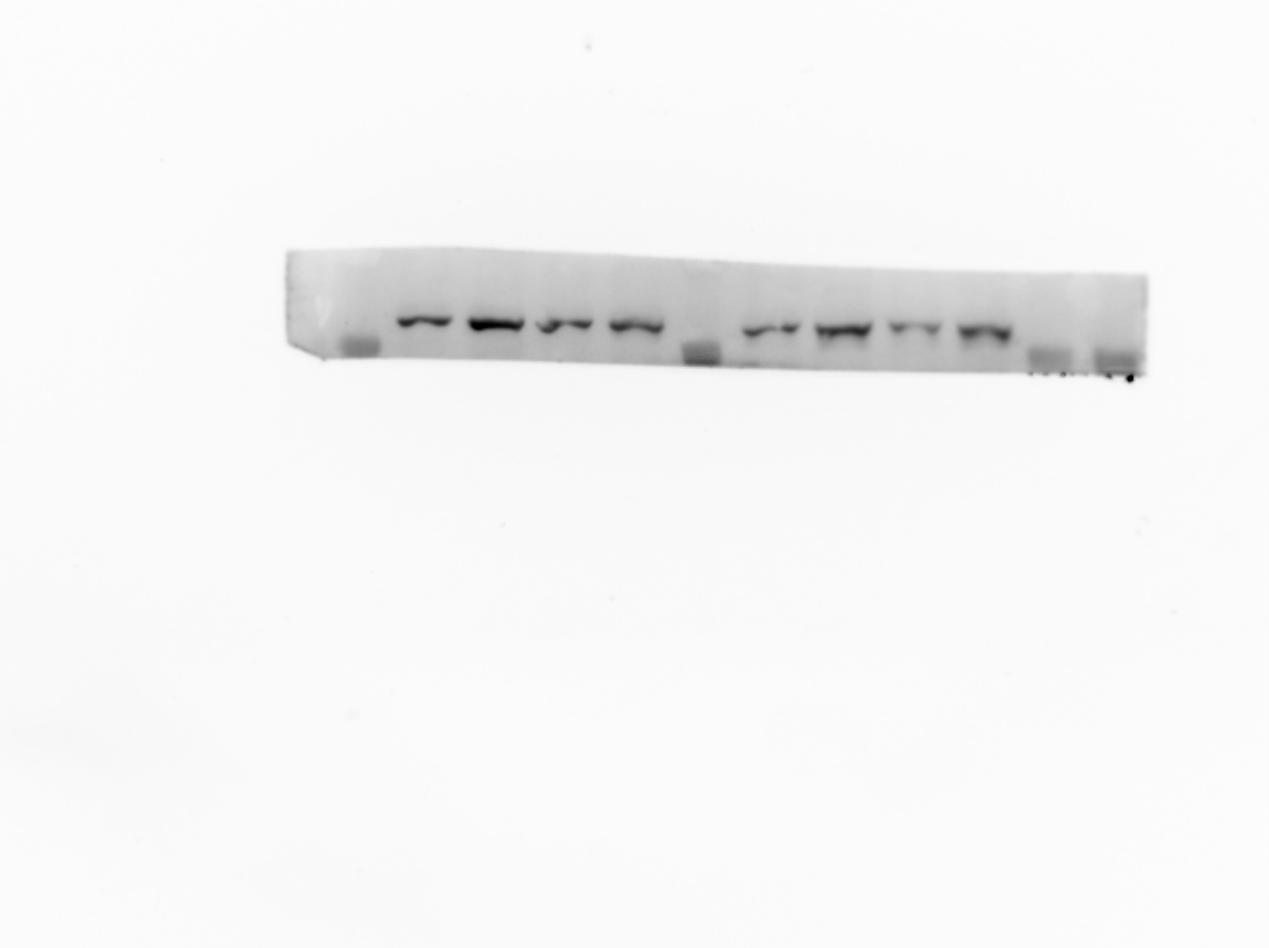
**

**Actin-U87MG**

**
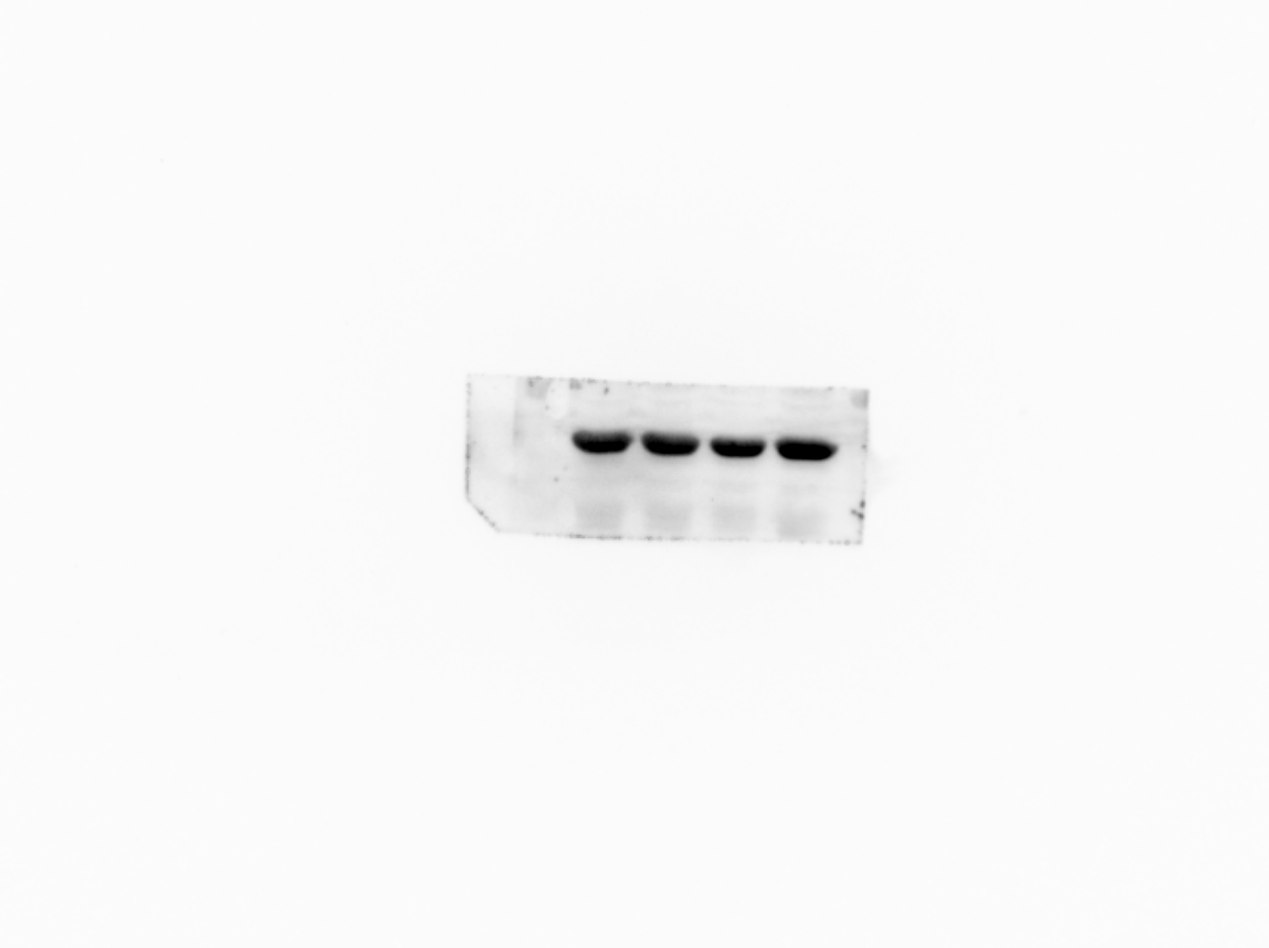
**

**FigS7.A**

**Snail2**


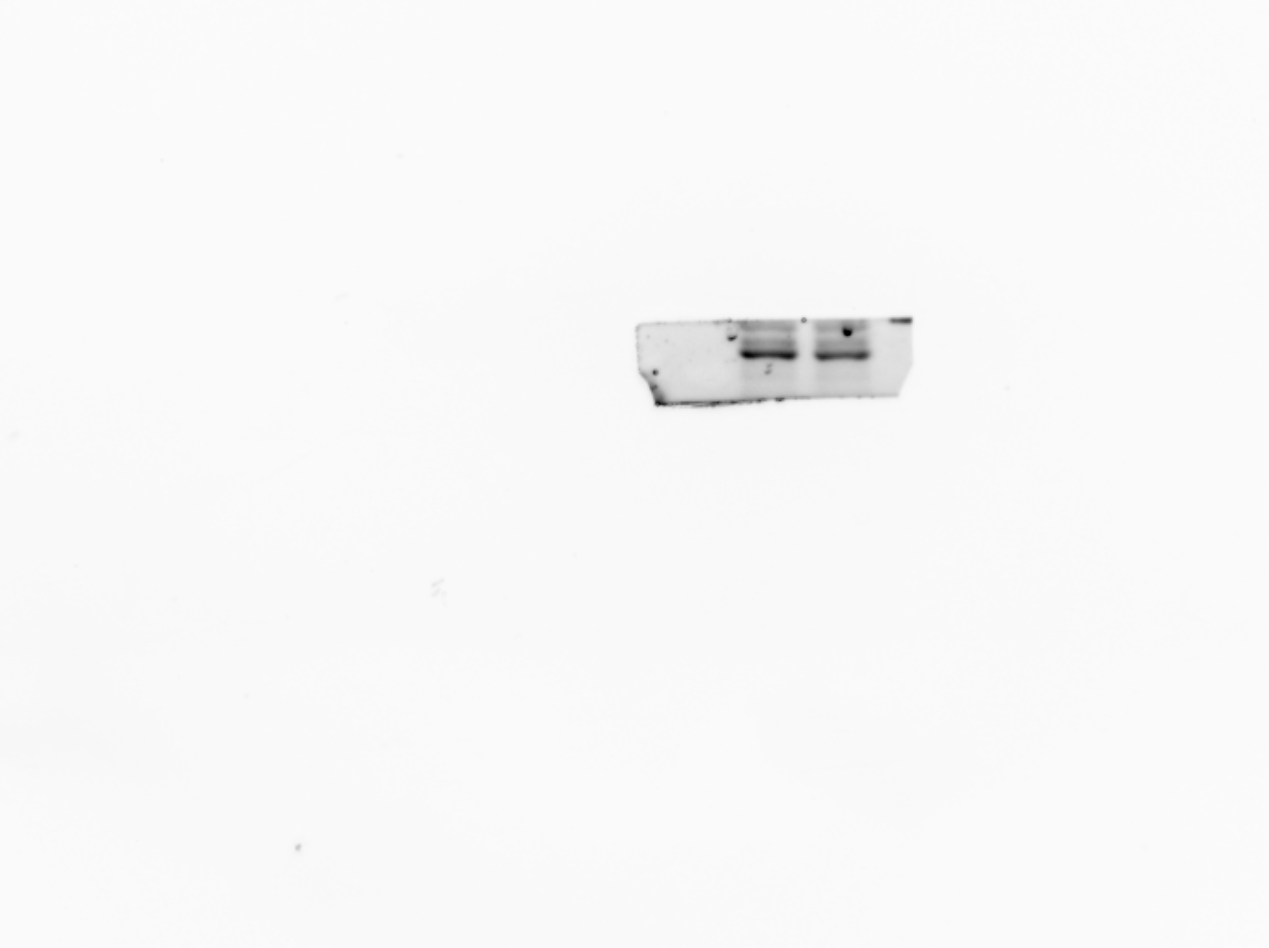


**E-cadherin**


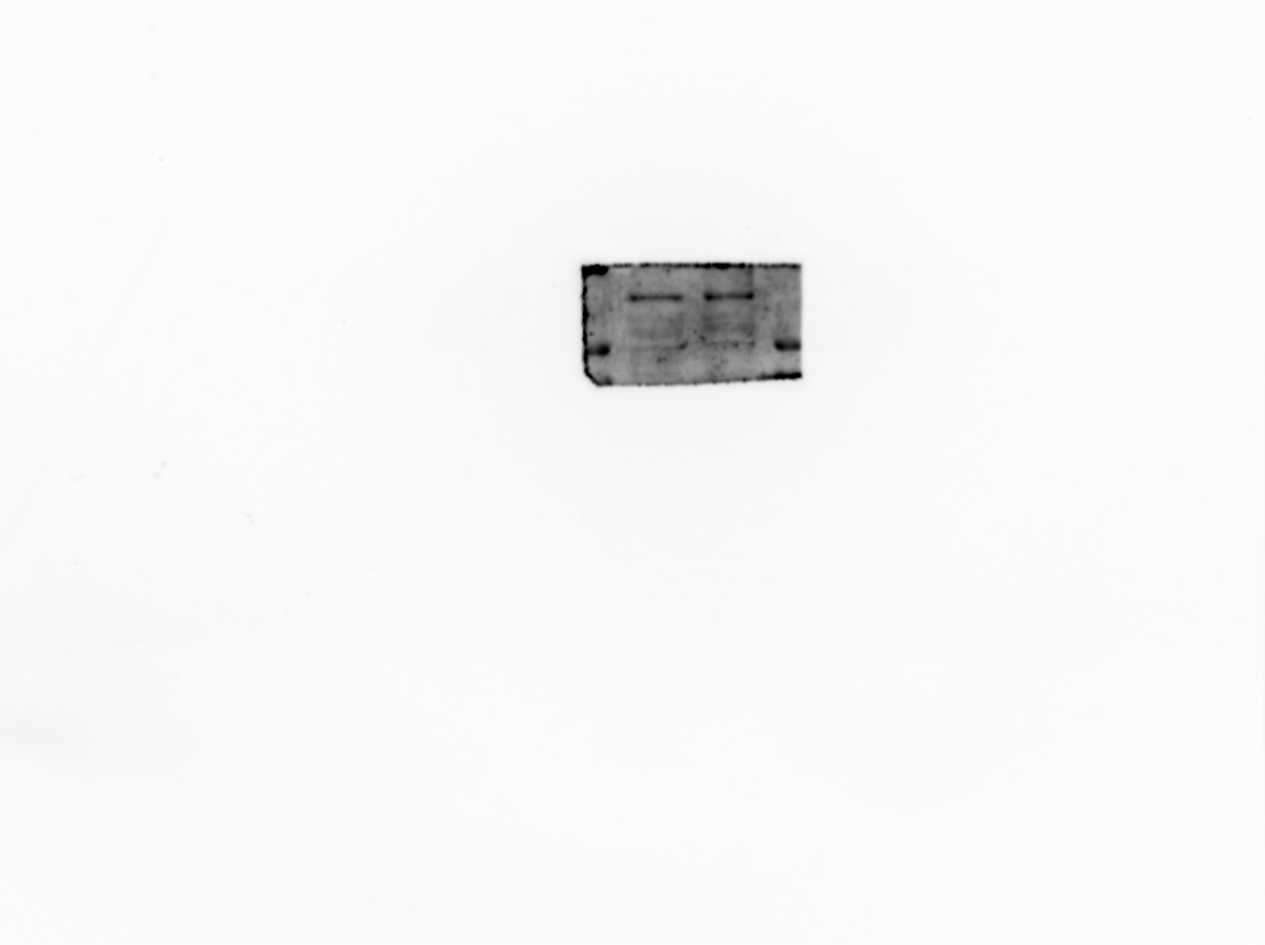


**N-cadherin**


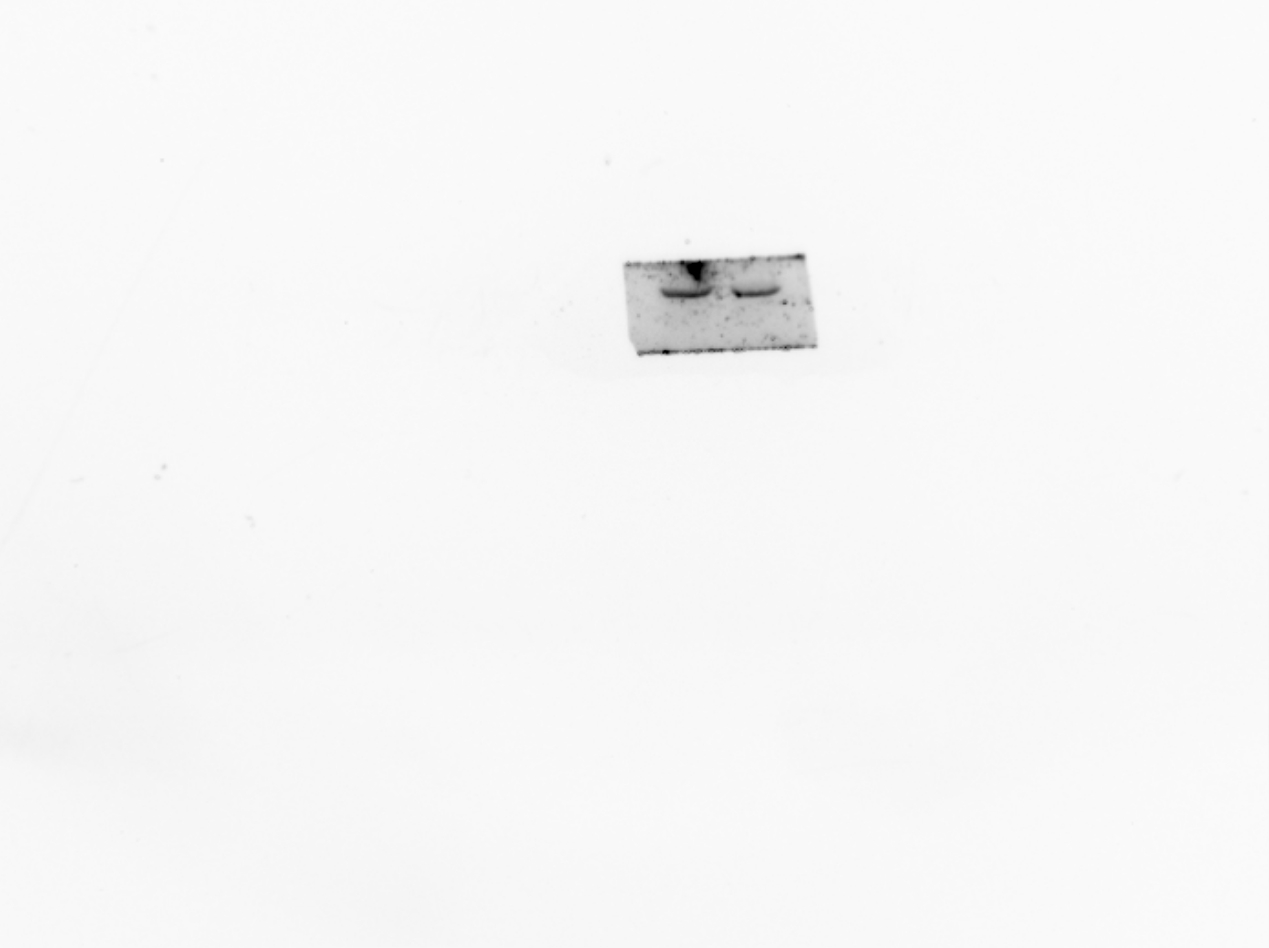


**Vimentin**


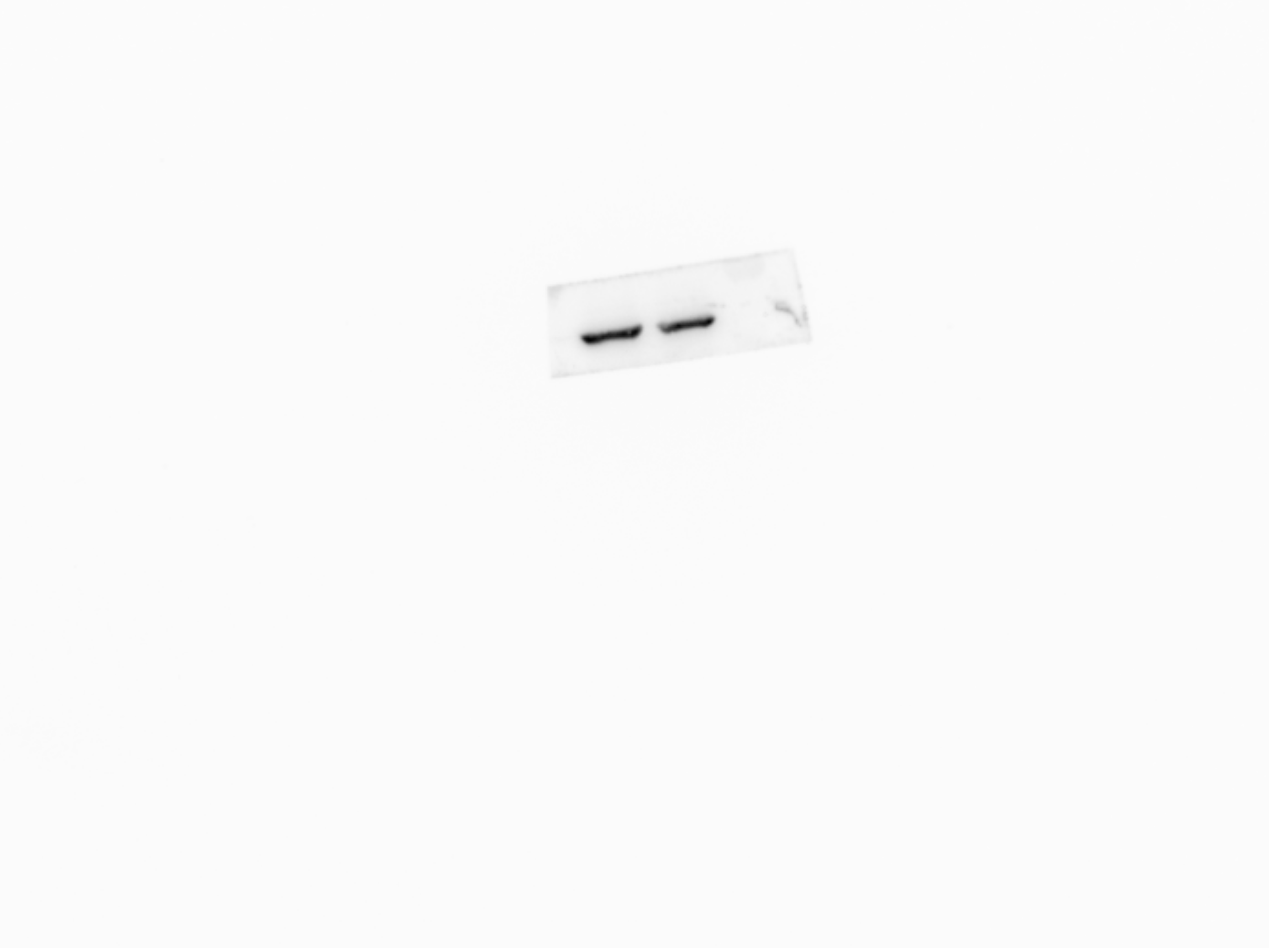


**β-Actin**


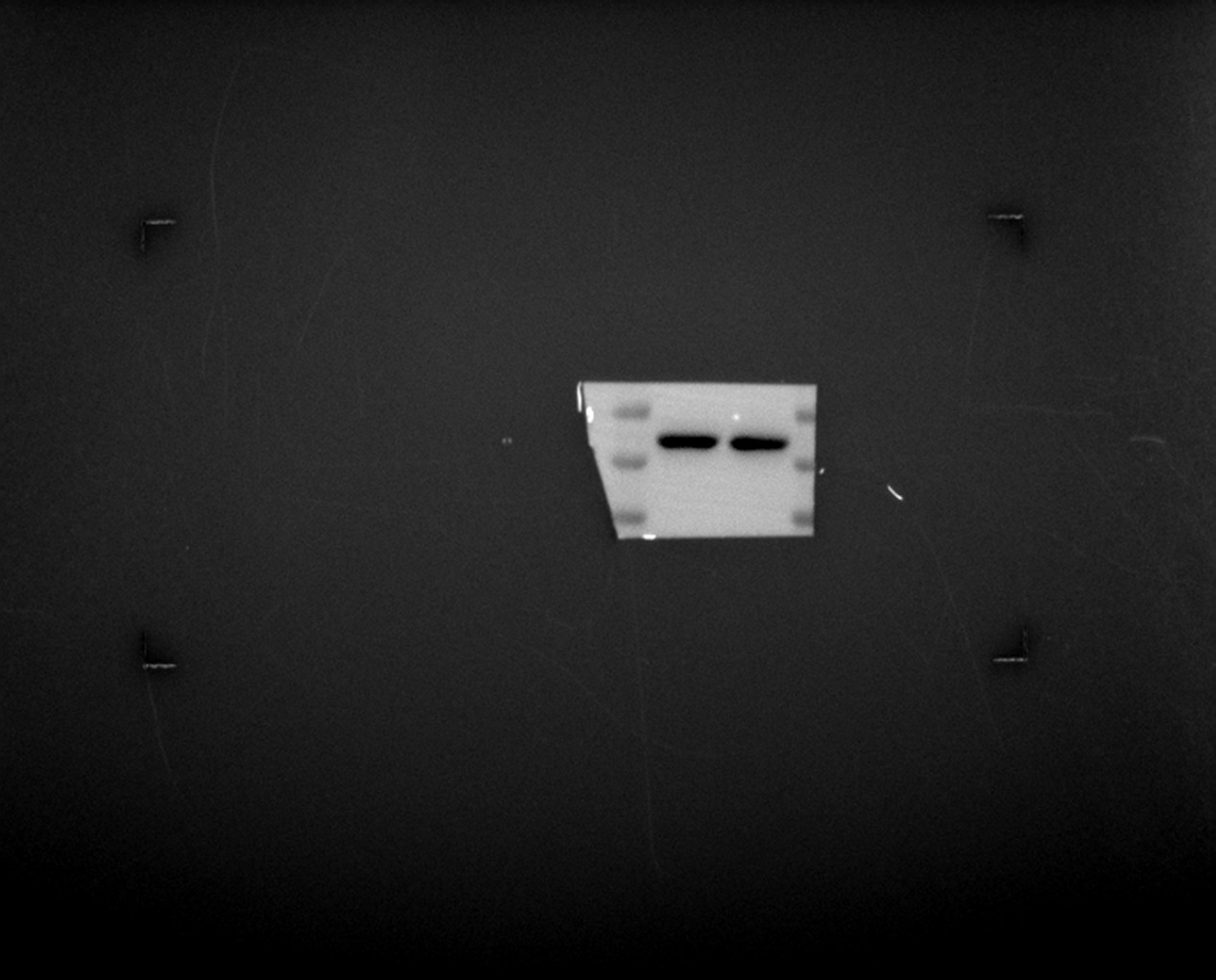

Supplement: Supplementary file 2 — Full and uncropped western blots [file 41419_2024_6936_MOESM2_ESM.docx]
